# Photocatalytic Aerobic Dehydrogenation of N-Heterocycles with Ir(III) Photosensitizers Bearing the 2(2′-Pyridyl)benzimidazole Scaffold

**DOI:** 10.1021/acs.inorgchem.2c00358

**Published:** 2022-04-08

**Authors:** Igor Echevarría, Mónica Vaquero, Blanca R. Manzano, Félix A. Jalón, Roberto Quesada, Gustavo Espino

**Affiliations:** † Departamento de Química, Facultad de Ciencias, Universidad de Burgos, Plaza Misael Bañuelos s/n, 09001 Burgos, Spain; ‡ Departamento de Química Inorgánica, Orgánica y Bioquímica, Facultad de Ciencias y Tecnologías Químicas, Universidad de Castilla-La Mancha, Avda. Camilo J. Cela 10, 13071 Ciudad Real, Spain

## Abstract

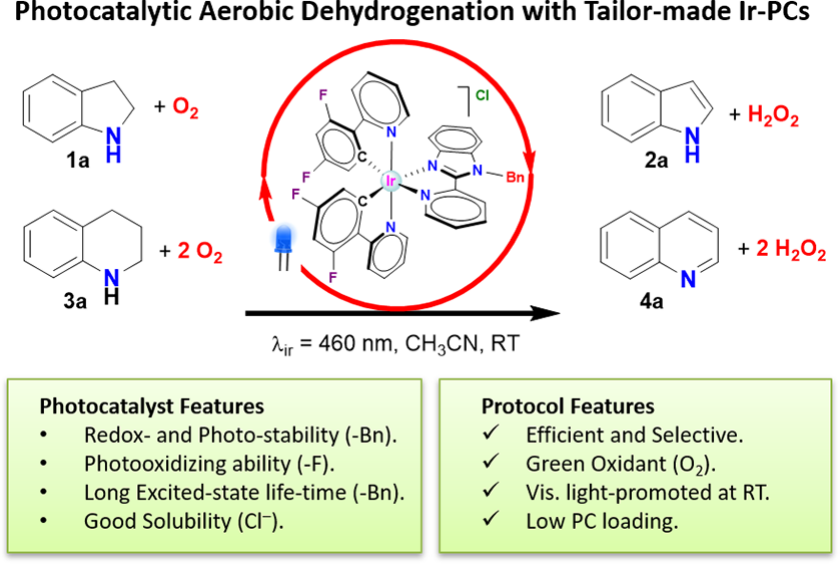

Photoredox catalysis constitutes a very powerful tool in organic synthesis, due to its versatility, efficiency, and the mild conditions required by photoinduced transformations. In this paper, we present an efficient and selective photocatalytic procedure for the aerobic oxidative dehydrogenation of partially saturated N-heterocycles to afford the respective N-heteroarenes (indoles, quinolines, acridines, and quinoxalines). The protocol involves the use of new Ir(III) biscyclometalated photocatalysts of the general formula [Ir(C^N)_2_(N^N′)]Cl, where the C^N ligand is 2-(2,4-difluorophenyl)pyridinate, and N^N′ are different ligands based on the 2-(2′-pyridyl)benzimidazole scaffold. In-depth electrochemical and photophysical studies as well as DFT calculations have allowed us to establish structure–activity relationships, which provide insights for the rational design of efficient metal-based dyes in photocatalytic oxidation reactions. In addition, we have formulated a dual mechanism, mediated by the radical anion superoxide, for the above-mentioned transformations.

## Abstract 2

This work describes the synthesis of a family of new Ir(III) biscyclometalated photosensitizers and their application as photocatalysts in the dehydrogenation of indolines, tetrahydroquinolines, etc., using visible light under mild conditions. A dual mechanism is rationally proposed, and an in-depth study of their electrochemical and photophysical properties provides insights for the rational design of efficient metal-based dyes in photocatalytic oxidation reactions.

## Introduction

N-heterocycles are pivotal scaffolds in the pharmaceutical industry due to their biological activity and medicinal applications.^1^ In particular, indoles,^2^ quinolines,^3,4^ acridines,^5,6^ and quinoxalines^7^ display anticancer, antibiotic, antibacterial, antifungal, and anti-inflammatory properties. Moreover, the redox couples formed by 1,2,3,4-tetrahydroquinolines (THQ) and the corresponding quinolines have been proposed as potential hydrogen-storage material systems for fuel cell applications, since the catalytic hydrogenation of quinolines takes place under mild reaction conditions and can be reverted through catalytic dehydrogenation protocols.^8^

Traditional procedures for preparing N-containing aromatic molecules from partially saturated N-heterocycles involve harsh reaction conditions (high temperatures), the use of stoichiometric toxic or corrosive oxidants (2,3-dichloro-5,6-dicyano-1,4-benzoquinone (DDQ), sulfur, or metal oxides), as well as the generation of undesirable waste.^9^

More recently, several groups have described methodologies to prepare different aromatic N-heterocycles (N-heteroarenes) from partially saturated precursors through either catalytic dehydrogenation^10^ or catalytic acceptorless dehydrogenation.^11,12^ Nevertheless, both strategies require high temperatures and/or harsh reaction conditions and, in some cases, harmful solvents and high catalyst loadings.

The synthesis of N-heteroarenes can also be accomplished through photocatalytic approaches such as the acceptorless dehydrogenation (ADH) of THQs, indolines and similar heterocycles. Different photocatalytic systems have been successfully used to prove this methodology, namely, combinations of a Ru-photocatalyst (PC) and a Co catalyst,^8,13^ or an acridinium PC and a Pd metal catalyst,^14^ and also heterogeneous PCs, that is, hexagonal boron carbon nitride nanosheets^15^ or Rh-photodeposited TiO_2_ nanoparticles.^16^ This transformation produces molecular hydrogen as the only byproduct, but it must be managed through expensive procedures when operating at high scale.

Alternatively, it is possible to access N-heteroarenes through oxidative dehydrogenation (ODH) of partially saturated precursors under aerobic photocatalytic conditions, which implies the use of O_2_ as the hydrogen acceptor (green oxidant), visible light, and a photosensitizer. In particular, the synthesis of a variety of N-heteroarenes (quinolines, quinoxalines, quinazolines, acridines, and indoles) has been performed using this type of strategy in the presence of different photocatalytic systems: [Ru(bpy)_3_]Cl_2_,^17^ Rose Bengal,^9^ TiO_2_ grafted with Ni(II) ions in the presence of 4-amino-TEMPO,^18^ and a cobalt-phthalocyanine photoredox catalyst in a biphasic medium.^19^ Nevertheless, there is still scope to explore new photosensitizers with the goal of increasing product yields, reducing reaction times and employing solvents with low boiling points. What is more, additional studies should be done for a better understanding of the reaction mechanism entailed in this type of transformations.

In a previous work, we have designed a family of new Ir(III) biscyclometalated complexes with β-carbolines as efficient photocatalysts for the one-pot oxidative thiocyanation of indolines, which produces the respective 3-thiocyanate indoles.^20^ We have also reported on a protocol to prepare α-amino nitriles through the Ru-photosensitized oxidative cyanation of amines.^21^

In this work, we present the synthesis of new Ir(III) biscyclometalated complexes of general formula [Ir(C^N)_2_(N^N′)]Cl, where C^N = 2-(2,4-difluorophenyl)pyridinate (dfppy) and N^N′ stands for different N,N-donor ligands containing the 2-(2′-pyridyl)benzimidazole scaffold. The ligand dfppy was chosen to obtain enhanced photoluminescent quantum yields and excited-state lifetimes, since this behavior is usually expected from the presence of electron-withdrawing groups, such as the −F atoms on the C^N ligands in this type of complexes.^22,23^ 2-(2′-pyridyl)benzimidazole was selected as the scaffold for the N^N′ ligands due to both its commercial availability and the presence of the imidazole N–H, which allows easy functionalization with a variety of alkyl groups. This, in turn, allows to explore the impact of different functional groups on the photophysical and photocatalytic properties of the resulting complexes (see below). In addition, we describe the evaluation of these complexes as photosensitizers in dehydrogenation processes. Furthermore, relationships between the photosensitizing abilities of these complexes and their electrochemical and photophysical properties are established. In particular, the effect of using dfppy as the C^N ligand and the influence of the different functional groups of the N^N′ ligands on the photocatalytic performance of our dyes are emphasized.

## Results and Discussion

### Synthesis of Ligands and Iridium(III) Complexes

**Figure 1:**
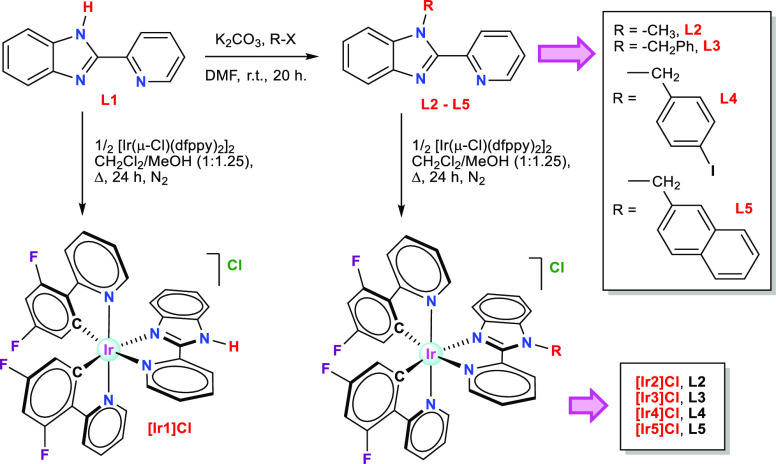
Synthesis route and molecular structures of ligands **L1**–**L5** and complexes [**Ir1**]**Cl–**[**Ir5**]**Cl**. Complexes were obtained as racemic mixtures but only Λ enantiomers are shown.

We have synthesized a family of Ir(III) biscyclometalated compounds of general formula *rac*-[Ir(C^N)_2_(N^N′)]Cl with the aim of developing new efficient photocatalysts. In this series of compounds, we have furnished the iridium center with two units of the anionic C^N donor 2-(2,4-difluorophenyl)pyridinate (dfppy) and five different N^N′ ligands based on the 2-(2′-pyridyl)benzimidazole scaffold (Hpybim = **L1**). The ligand 2-(2′-pyridyl)benzimidazole (**L1**) is commercially available, and its N-functionalized derivatives (**L2**–**L5**) were prepared by reacting **L1** with MeI, for **L2**, or the appropriate alkyl bromide (R-Br), for **L3**–**L5**, at room temperature in the presence of K_2_CO_3_, using DMF as solvent (see Figure 1).^24−26^ The incorporation of diverse alkyl groups into the N^N′ ligand aimed to reduce intermolecular interactions and to assess different effects on the photophysical and photocatalytic properties of the resulting Ir derivatives. Thus, the methyl and benzyl groups (**L2**, **L3**, and **L4**) were chosen to protect the respective complexes from either self-quenching or N–H reactivity. The naphthalenylmethyl group (**L5**) was used to evaluate the potential beneficial effect of a π-extended system on the absorption profile of its Ir derivative.

The Ir(III) compounds [**Ir1**]**Cl**–[**Ir5**]**Cl** were obtained by refluxing the iridium dimer [Ir(μ-Cl)(dfppy)_2_]_2_ (dfppy = 2-(2,4-difluorophenyl)pyridinate) in the presence of ligands **L1**–**L5** (1:2 molar ratio) in a dichloromethane-methanol mixture (1:1.25; v/v) (Figure 1). The products were isolated in the form of bright yellow solids, as chloride salts of racemic mixtures corresponding to the Δ and Λ cationic complexes (helical chirality).

The synthesis of the PF_6_^–^ salts of complexes **[Ir1**]^**+**^ and [**Ir2**]^**+**^ has been previously described, but to the best of our knowledge, their photocatalytic activity has not been studied so far.^27,28^

### Characterization of the Ir(III) Complexes

The iridium derivatives were unequivocally characterized by multinuclear NMR, mass spectrometry, elemental analysis, and IR spectroscopy. In addition, the crystal structures of **[Ir1]Cl** and the PF_6_^–^ salts of **[Ir3]**^**+**^, **[Ir4]**^**+**^, and **[Ir5]**^**+**^ were determined by X-ray diffraction.

The ^1^H and ^13^C{^1^H} spectra of complexes **[Ir1]Cl**–**[Ir5]Cl** were recorded in DMSO-*d*_6_ (Figures S1–S15) and show the following distinctive attributes: (1) The nonequivalent dfppy ligands exhibit two sets of peaks, due to the asymmetry of the complexes (*C*_1_ symmetry group); (2) **[Ir1]Cl** displays a strongly deshielded broad singlet (δ = 15.91 ppm) due to the N–H proton which is likely involved in a hydrogen bond with the Cl^–^ counterion,^29^ while **[Ir2]Cl** features a narrow singlet at 4.48 ppm belonging to the N-Me group; (3) for complexes **[Ir3]Cl**–**[Ir5]Cl**, two reciprocally coupled doublets (^2^*J*_H–H_ ≈ 18 Hz), emerging as an AB pseudoquartet, were found in the range 6.52–6.24 ppm and are attributed to the diastereotopic protons of the −CH_2_ groups as a result of the helical chirality typical of tris-chelate octahedral complexes, while for the achiral free ligands **L3**–**L5**, the −CH_2_ group appears as a singlet integrating for 2 H; (4) complexes **[Ir3]Cl**–**[Ir5]Cl** exhibit a singlet around 48 ppm for the −CH_2_ group in their ^13^C{^1^H} NMR spectra.

The ^19^F NMR spectra of all the derivatives feature two quartets in the range between −106.5 and −107 ppm (F^11^ and F^11’^) and two triplets at about −109 ppm (F^9^ and F^9’^), for the two nonequivalent dfppy (see atom numbering in the Supporting Information (SI)).

The HR-MS (ESI+) spectra of the Ir(III) complexes present peaks where the *m*/*z* values and the isotopic patterns match unambiguously with those calculated for the monocationic species of general formula [Ir(dfppy)_2_(N^N′)]^+^ (N^N′ = **L1–L5**). We also detected for all the compounds a peak corresponding to the monocationic fragment [C_22_H_12_F_4_IrN_2_]^+^ = [Ir(dfppy)_2_]^+^, which corresponds to the loss of the N^N′ ligand.

### Crystal Structure by X-ray Diffraction

**Figure 2:**
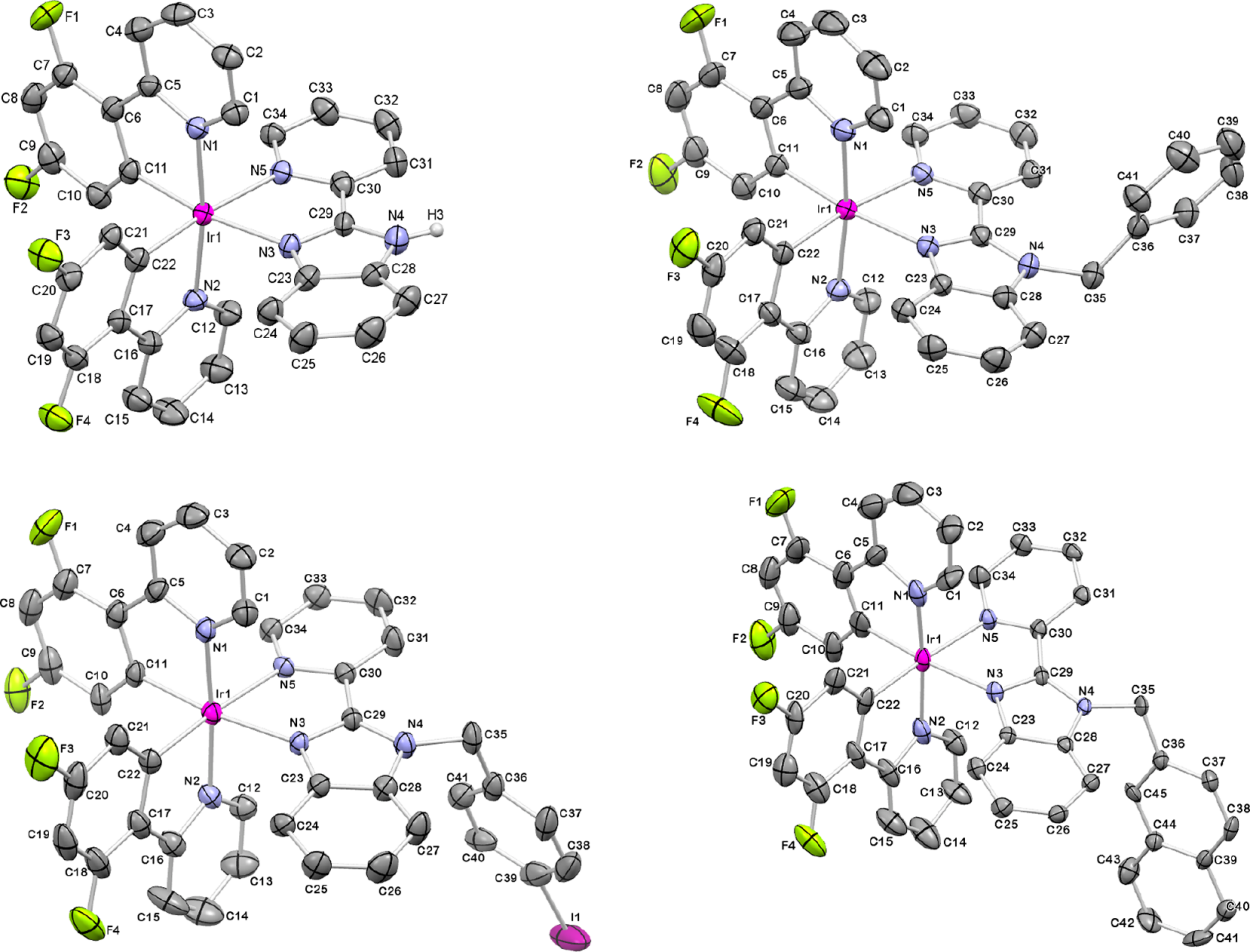
ORTEP diagrams for the molecular structures of (Λ)-[**Ir1**]^+^, (Λ)-[**Ir3**]^+^, (Λ)-[**Ir4**]^+^ and (Λ)-[**Ir5**]^+^ obtained by X-ray diffraction. Thermal ellipsoids are shown at the 30% probability level. The Δ enantiomers, the H atoms, the Cl^–^ or PF_6_^–^ counterions, and the solvent molecules (MeOH for *rac*-**[Ir1]Cl**) have been omitted for the sake of clarity.

The crystal structures of *rac***-[Ir1]Cl**, *rac***-[Ir3]PF**_**6**_, *rac***-[Ir4]PF**_**6**_, and *rac***-[Ir5]PF**_**6**_ were resolved by single-crystal X-ray diffraction. Single crystals were isolated either by slow evaporation of a methanolic solution of **[Ir1]Cl** or by slow diffusion of a saturated NH_4_PF_6_ aqueous solution into solutions of **[Ir3]Cl**, **[Ir4]Cl**, and **[Ir5]Cl** in methanol/dichloromethane. The complexes crystallize in either the monoclinic *P*2_1/*c*_ or *P*2_1/*n*_, or triclinic *P*-1 space groups. The ORTEP diagrams for the corresponding Λ enantiomers are shown in Figure 2. Selected bond distances and angles along with standard deviations are collected in Table 1, and relevant crystallographic parameters are included in Table S1.

**Table 1:** Selected Bond Lengths (Å) for **[Ir1]Cl·MeOH**, **[Ir3]PF**_**6**_, **[Ir4]PF**_**6**_, and **[Ir5]PF**_**6**_

**[Ir1]Cl·MeOH**		**[Ir3]PF_6_**		**[Ir4]PF_6_**		**[Ir5]PF_6_**			
Ir(1)–N(1)	2.054(5)	Ir(1)–N(1)	2.042(4)	Ir(1)–N(1)	2.059(7)	Ir(1)–N(1)	1.963(12)	Ir(2)–N(7)	2.046(8)
Ir(1)–N(2)	2.058(5)	Ir(1)–N(2)	2.054(4)	Ir(1)–N(2)	2.043(7)	Ir(1)–N(2)	2.074(12)	Ir(2)–N(6)	2.032(8)
Ir(1)–N(3)	2.138(5)	Ir(1)–N(3)	2.142(4)	Ir(1)–N(3)	2.119(6)	Ir(1)–N(3)	2.131(8)	Ir(2)–N(9)	2.119(8)
Ir(1)–N(5)	2.179(5)	Ir(1)–N(5)	2.153(4)	Ir(1)–N(5)	2.158(6)	Ir(1)–N(5)	2.163(8)	Ir(2)–N(8)	2.181(7)
Ir(1)–C(11)	2.020(6)	Ir(1)–C(11)	2.019(5)	Ir(1)–C(11)	2.013(8)	Ir(1)–C(11)	1.995(13)	Ir(2)–C(67)	2.000(10)
Ir(1)–C(22)	2.018(6)	Ir(1)–C(22)	2.005(5)	Ir(1)–C(22)	2.002(7)	Ir(1)–C(22)	2.015(11)	Ir(2)–C(56)	2.003(9)

The molecular structures of these complexes display a pseudo-octahedral geometry with the well-known *trans*-N,N and *cis*-C,C arrangement for the C^N ligands (Figure 2). In all the derivatives, the Ir–N bond distances for the C^N ligands (1.963(12)–2.074(12) Å) are shorter than for the N^N′ ligands (2.119(6)–2.181(7) Å) as a consequence of the strong *trans* influence exerted by the coordinated phenyl rings.^30−33^ Besides, the Ir–N_bim_ length is shorter than the Ir–N_py_ in the N^N′ ligand of every complex, likely due to the bigger π-electron density on the benzimidazole (bim) fragment relative to the pyridine (py) ring and therefore the higher π-donor ability of bim versus py. The Ir–C bond distances are standard (1.995(13)–2.020(6) Å).^34,35^ The torsion angles for the C^N and the N^N′ ligands, C–C–C–N (−0.02 to −6.22°) and N–C–C–N (1.57 to −18.99°), are small, which in practice underlines the coplanarity of the metallacycles.

### Photostability Experiments

In order to verify the photostability in solution of the new Ir(III)-complexes and the standard photocatalysts [Ir(ppy)_2_(bpy)]Cl and [Ir(dfppy)_2_(bpy)]Cl (denoted as **[1]Cl** and **[2]Cl**), we monitored their evolution in acetonitrile under air by ^1^H NMR spectroscopy (1.4 × 10^–2^ M, CD_3_CN) over a period of 24 h under irradiation with blue light (λ_ir_ = 460 nm, 24 W) at room temperature (Figures S17–S22). All the complexes including **[1]Cl** and **[2]Cl** are remarkably stable over the 24 h irradiation period. Indeed, no degradation was observed for **[Ir1]Cl–[Ir4]Cl**, and just 3% photodegradation was experimentally determined for **[Ir5]Cl**. An in-depth analysis of the spectrum, recorded for this PC upon 24 h under light exposure, allowed us to speculate that it undergoes photocleavage of the −CH_2_-naphthyl group.

### Theoretical Calculations

Density functional theory (DFT) calculations were performed on the cation complexes **[Ir1]**^**+**^**–[Ir5]**^**+**^ and also on the reference photosensitizers [Ir(ppy)_2_(bpy)]^+^, **[1]**^**+**^, and [Ir(dfppy)_2_(bpy)]^+^, **[2]**^**+**^, for a deeper comprehension of the photophysical and electrochemical properties of the synthesized compounds and to rationalize the observed trends among them and relative to **[1]**^**+**^ and **[2]**^**+**^. Calculations were executed at the B3LYP/(6-31GDP+LANL2DZ) level including solvent effects (CH_3_CN) (see procedure in the SI and Tables S2a, S2b, and S3).

**Figure 3:**
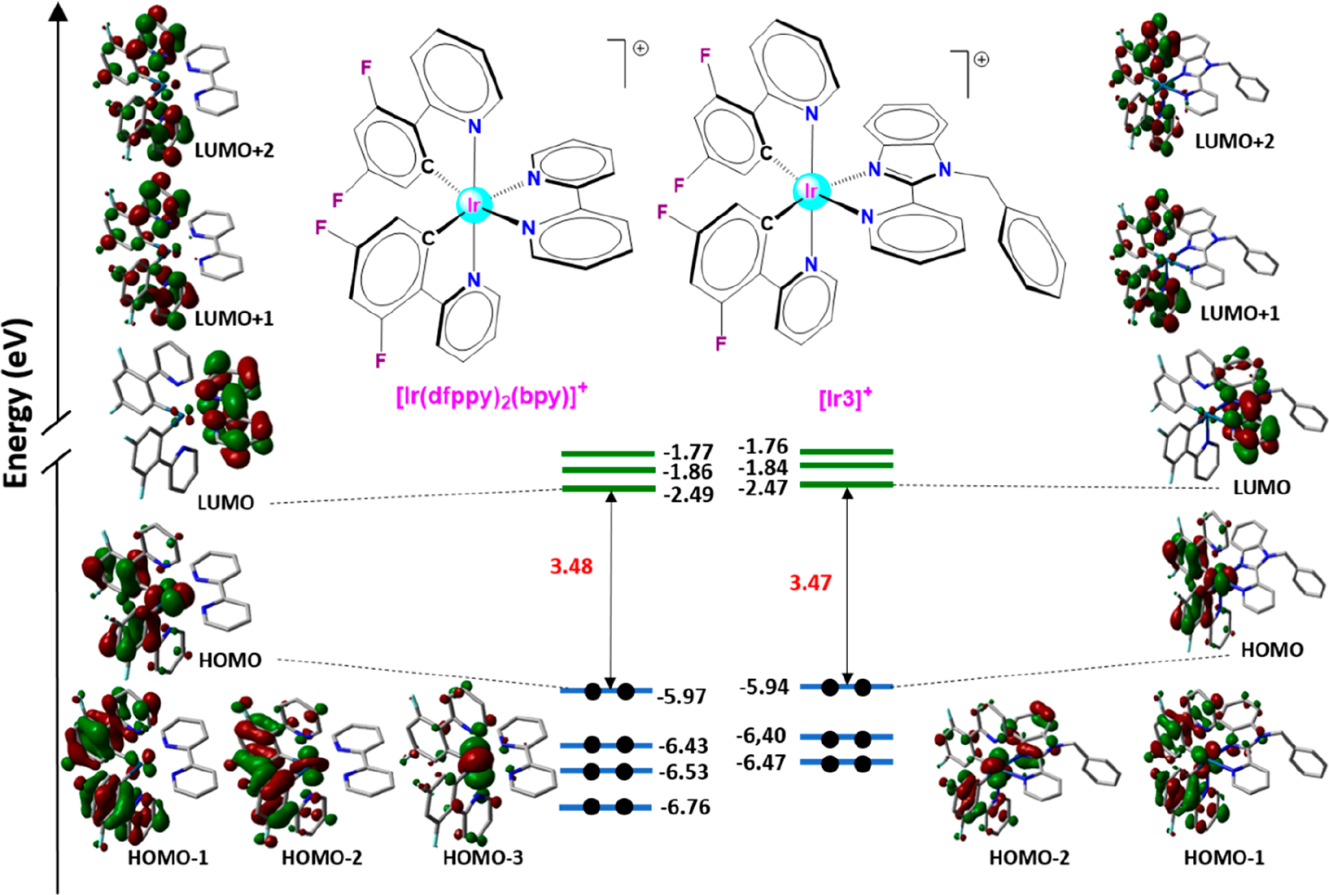
Schematic representation of the energies and the isovalue contour pictures calculated for the frontier molecular orbitals of [Ir(dfppy)_2_(bpy)]^+^, **[2]**^**+**^, and **[Ir3]**^**+**^.

In agreement with the molecular structure determined by X-ray diffraction for **[Ir1]Cl**, **[Ir3]PF**_**6**_, **[Ir4]PF**_**6**_, and **[Ir5]PF**_**6**_, our calculations provide structures with a pseudo-octahedral geometry for **[Ir1]**^**+**^–**[Ir5]**^**+**^, **[1]**^**+**^, and **[2]**^**+**^ in their ground electronic state (S_0_). Figure 3 shows the isovalue contour pictures for the molecular orbitals, from HOMO–2 (or HOMO–3) to LUMO+2, of **[Ir3]**^**+**^ and **[2]**^**+**^ at their electronic ground state (S_0_). A similar sketch is shown in Figure S23b for **[Ir3]**^**+**^ and **[1]**^**+**^. The topologies of the MOs for **[Ir1]**^**+**^, **[Ir2]**^**+**^ and **[Ir4]**^**+**^ are very similar to those of **[Ir3]**^**+**^. By contrast, the MO of **[Ir5]**^**+**^ exhibit some differences that will be discussed later. The MOs of all the compounds are gathered in Tables S2a and S2b.

The HOMOs calculated for **[1]**^**+**^, **[2]**^**+**^, and the new derivatives are formed by a combination of Ir orbitals (d_π_) and C^N orbitals (π of ppy^–^ or dfppy^–^) as described elsewhere for this type of complexes.^20,36,37^ Hence, the HOMOs are located on the Ir metal center and the phenyl rings of the C^N ligands, although they exhibit a π-antibonding nature at the Ir–C_phenyl_ interfaces. On the contrary, the LUMOs are distributed mainly over the N^N′ ligands (bpy or 2-(2′-pyridyl)benzimidazole scaffold of **L1**–**L5**) in all the cases,^37,38^ with a very small contribution of the Ir d_π_ orbitals. Interestingly, the alkyl substituents installed on the N^N′ ligands of **[Ir2]**^**+**^–**[Ir4]**^**+**^ do not participate in the respective frontier orbitals, while the naphthyl group of **[Ir5]**^**+**^ contributes predominantly to HOMO–1 and LUMO+4. Hence, we assume that the alkyl groups of **[Ir2]**^**+**^–**[Ir4]**^**+**^ are not involved in photophysical processes, that is, absorption or emission of photons, and therefore these moieties act as protecting shields for the PC emitting excited states against decay processes.^39^

The energies calculated for the HOMOs of **[Ir1]**^**+**^–**[Ir5]**^**+**^ and **[2]**^**+**^ are in a very small range (from −5.93 to −5.97 eV, Figure 3 and Figure S23a), but are noticeably lower than the energy obtained for the HOMO of **[1]**^**+**^ (−5.65 eV). This effect is ascribed to the electron-withdrawing ability of the −F atoms in dfppy, which leads to a remarkable stabilization of the HOMO in **[Ir1]**^**+**^–**[Ir5]**^**+**^ and **[2]**^**+**^ relative to **[1]**^**+**^.^28,40^

The energies calculated for the LUMOs of **[Ir1]**^**+**^–**[Ir5]**^**+**^ and **[2]**^**+**^ are also very similar (from −2.45 to −2.49 eV, Figure 3 and Figure S23) and slightly lower than that estimated for the LUMO of **[1]**^**+**^ (−2.41 eV). These energies show a negligible influence of either the replacement of a pyridine ring with a benzimidazole unit or the installation of different substituents on the N atom of **L1**–**L5**. Consequently, the HOMO–LUMO band gaps for **[Ir1]**^**+**^–**[Ir5]**^**+**^ and **[2]**^**+**^ (3.45–3.49 eV) are in a narrow range, but they are significantly higher than that corresponding to **[1]**^**+**^ (3.23 eV). These tendencies are in agreement with those observed experimentally for the electrochemical band gaps (*vide infra*).

**Figure 4:**
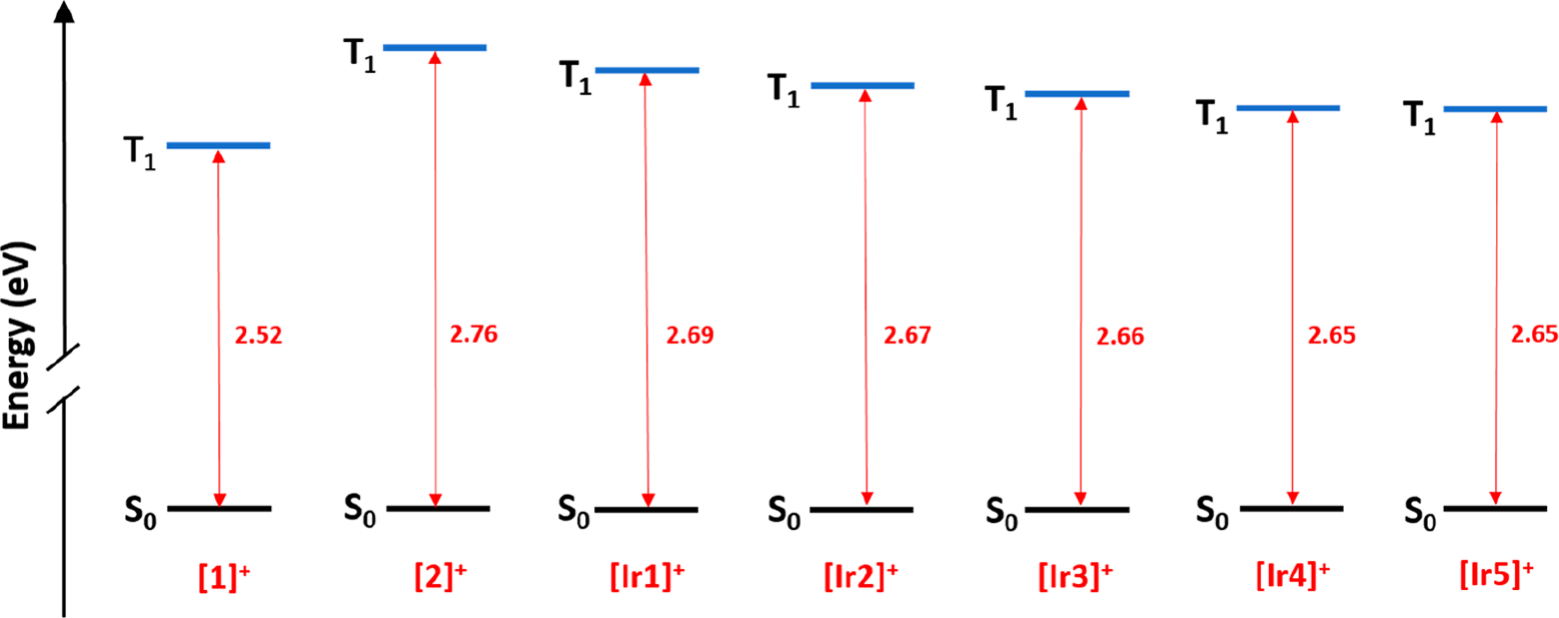
Energy diagram showing the calculated energy difference values between the lowest triplet excited state (T_1_) and the singlet ground state, keeping the geometry of the respective triplet (S_0_) for complexes **[1]**^**+**^, **[2]**^**+**^, and **[Ir1]**^**+**^–**[Ir5]**^**+**^.

The nature of the emitting excited states and the emission energies for the new compounds (T_1_ – S_0_) were calculated using the time-dependent DFT (TD-DFT) method (Figure 4). The obtained values predict very similar emission λ_max_ for **[Ir1]**^**+**^–**[Ir5]**^**+**^ and **[2]**^**+**^, although a blue-shift relative to the respective λ_max_ for **[1]**^**+**^ is also predicted. All these estimations are consistent with the emission energies determined experimentally (see next section) and establish that the presence of the electron-withdrawing −F atoms on the C^N ligands is the main factor affecting the emission energies.

### Photophysical Properties

#### UV–vis Absorption Spectra

**Figure 5:**
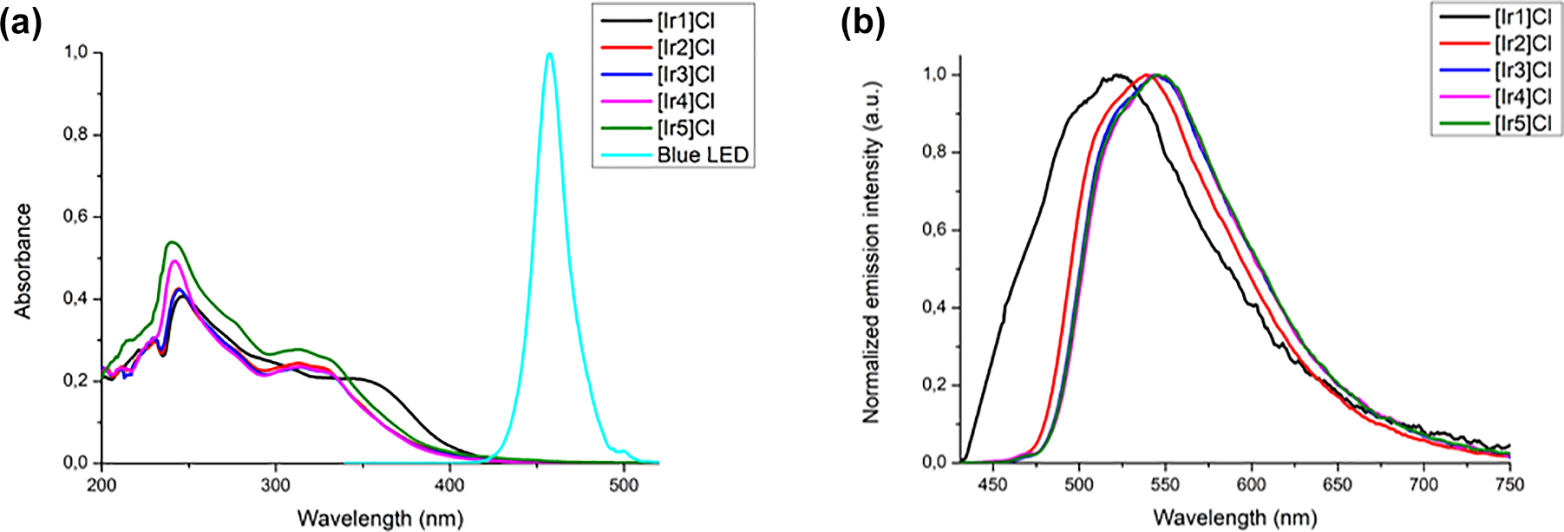
(a) Overlaid UV–vis absorption spectra of **[Ir1]Cl**–**[Ir5]Cl** (10^–5^ M) in CH_3_CN at 25 °C along with the emission spectrum of the blue light used in the photocatalytic assays (left). (b) Overlaid emission spectra of of **[Ir1]Cl**–**[Ir5]Cl** (10^–5^ M) in deoxygenated CH_3_CN at 25 °C upon excitation with λ_ex_ = 405 nm (right).

The UV–vis spectra of complexes **[Ir1]Cl**–**[Ir5]Cl** were recorded in acetonitrile solutions (10^–5^ M) at 25 °C (Figure 5a). The absorption spectra of complexes **[Ir1]Cl**–**[Ir5]Cl** show one intense absorption band centered at around 250 nm, which corresponds to singlet spin-allowed ligand centered transitions (^1^LC, π → π*) occurring in both types of ligands, the C^N (dfppy) and the N^N′. Additional bands are observed at around 313 nm for **[Ir2]Cl**–**[Ir5]Cl** and 348 nm for **[Ir1]Cl**. These bands are attributed to mixed spin-allowed ^1^MLCT and ^1^LLCT transitions. The weak absorption tails entering in the visible region come from spin-forbidden ^3^MLCT and ^3^LC transitions.^41−43^ In general, the absorption bands of **[Ir5]Cl** are more intense and are more extended in the range between 420 and 500 nm, and hence overlap better with the emission band of the light source used in photocatalytic assays (Figure 5a). This is likely due to the higher π-conjugation of the naphthyl group.

#### Emission Spectra

The emission spectra of complexes **[Ir1]Cl**–**[Ir5]Cl** were recorded in solutions of dry and deoxygenated acetonitrile (10^–5^ M) at 25 °C under excitation at 405 nm (see Figure 5b). All the spectra are alike, featuring a broad unstructured emission band, typical of high charge-transfer character.^40^ These bands have an absolute maximum between 522 and 546 nm for **[Ir1]Cl**–**[Ir5]Cl** (Table 2), which resembles the value reported for **[2]PF**_**6**_ (λ_em_ = 534 nm). Nevertheless, the emission of all these complexes is blue-shifted relative to that of the archetypal photosensitizer **[1]PF**_**6**_ (602 nm), as anticipated by DFT calculations.

**Table 2:** Photophysical Properties for Complexes **[Ir1]Cl**–**[Ir5]Cl** (10^–5^ M) in deaerated CH_3_CN at 25 °C under λ_ex_ = 405 nm^a^

complex	λ_abs_ (nm)	ε (M^–1^ cm^–1^)	λ_em_ (nm)	Φ_PL_	τ (ns)	*k*_r_ (s^–1^)^b^	*k*_nr_ (s^–1^)^c^
**[Ir(ppy)**_**2**_**(bpy)]**^**+**^	265, 310, 375, 420	–	602	0.093	275	3.38 × 10^5^	33 × 10^5^
**[Ir(dfppy)**_**2**_**(bpy)]**^**+**^	250, 305	–	534	0.18	1500	1.20 × 10^5^	5.47 × 10^5^
**[Ir1]Cl**	233, 246, 348	0.303, 0.408, 0.206	522	0.78	59	132 × 10^5^	37.3 × 10^5^
**[Ir2]Cl**	238, 244, 319, 331	0.348, 0.428, 0.239, 0.226	539	0.36	2066	1.74 × 10^5^	3.10 × 10^5^
**[Ir3]Cl**	237, 244, 320, 333	0.328, 0.426, 0.229, 0.215	544	0.63	1321	4.77 × 10^5^	2.80 × 10^5^
**[Ir4]Cl**	244, 322, 334	0.487, 0.228, 0.213	544	0.46	1510	3.05 × 10^5^	3.58 × 10^5^
**[Ir5]Cl**	244, 321, 333	0.533, 0.268, 0.249	546	0.09	1012	0.89 × 10^5^	8.99 × 10^5^

a Data for **[1]PF**_**6**_ and **[2]PF**_**6**_ reported by E. Zysman-Colman^47,49^ and De Cola,^48^ respectively.

b Radiative deactivation rate constant: *k*_r_ = φ_PL_ × τ^–1^.

c Nonradiative deactivation rate constant: *k*_nr_ = τ^–1^ – *k*_r_ (assuming unitary intersystem crossing efficiency).

The photoluminescence quantum yields (PLQY, Φ_PL_) were also determined in deoxygenated acetonitrile solutions (10^–5^ M). **[Ir1]Cl** and **[Ir3]Cl** display very good quantum yields of 0.78 and 0.63, respectively (Table 2). On the other hand, **[Ir2]Cl** and **[Ir4]Cl** feature moderate quantum yields of 0.36 and 0.46, respectively, while **[Ir5]Cl** shows a low quantum yield (0.09) similar to that for **[1]PF**_**6**_ (0.093) and lower than that for **[2]PF**_**6**_ (0.18). We speculate that the lower Φ_PL_ values determined for **[Ir2]Cl**–**[Ir5]Cl** versus **[Ir1]Cl** are mainly due to the intramolecular rotation of the *N*-alkyl groups in solution, which favors the dissipation of energy by nonradiative channels for these complexes.^44,45^ In addition, the very low PLQY of **[Ir5]Cl** could be the result of an extra factor, that is, the thermal population of a ligand-centered (^3^LC, π_L5_ → π*_L5_) excited state, (T_2_, 2.70 eV) close in energy to the emissive lowest excited state (T_1_, 2.65 eV) (Table S3). This feature provides a nonradiative decay pathway to **[Ir5]Cl**, since the nonparticipation of the metal center in T_2_ hampers the intersystem crossing process, and hence a low PLQY is observed.^46^ Therefore, we conclude that the functional group on the N^N′ ligand exerts an important influence in the efficiency of the emission process.

The excited-state lifetimes (τ) are excellent for the substituted derivatives **[Ir2]Cl**–**[Ir5]Cl**, between 1012 and 2066 ns and much longer than that for **[1]PF**_**6**_, whereas for the nonfunctionalized compound, **[Ir1]Cl**, τ is much shorter, 59 ns (Table 2). Hence, the functionalization of the imidazolyl nitrogen has also an important effect on the lifetimes of the excited states. In particular, we speculate that the presence of the N–H group in **[Ir1]Cl** could accelerate the radiative deactivation of the excited state relative to its functionalized counterparts **[Ir2]Cl**–**[Ir5]Cl**. The rationale for this could be that the ground state (S_0_) of **[Ir1]**^**+**^ is stabilized in acetonitrile solution through N–H—Cl^–^ or N–H—N≡C–Me hydrogen-bonding interactions. By contrast, in the excited state, which exhibits partial ^3^MLCT nature, the charge transfer from the metal center to the π* orbital of the N^N′ ligand decreases the polarization of the N–H bond and therefore the strength of the interaction with either the Cl^–^ counterion or the solvent molecules, shortening the lifetime of the triplet excited state (T_1_). In **[Ir2]Cl**–**[Ir5]Cl**, the presence of bulky apolar alkyl groups impedes hydrogen-bonding interactions and therefore avoids the differential stabilization of S_0_ relative to T_1_. This would explain the longer lifetimes observed for the excited states of **[Ir2]Cl**–**[Ir5]Cl** vs **[Ir1]Cl**.

The radiative and nonradiative deactivation rate constants, *k*_r_ and *k*_nr_, were calculated from Φ_PL_ and τ and are summarized in Table 2. It is worth noting that **[Ir1]Cl** has a *k*_r_ < *k*_nr_, while **[Ir2]Cl**–**[Ir4]Cl** exhibit similar values for *k*_r_ and *k*_nr_ and **[Ir5]Cl** features a *k*_nr_ 1 order of magnitude higher than *k*_r_.

Overall, the photophysical properties of our photosensitizers are in general superior to those reported for **[1]PF**_**6**_(47) and **[2]PF**_**6**_, and *a priori* the long lifetimes of **[Ir2]Cl**–**[Ir5]Cl** could favor their interaction with O_2_ to generate ROS.

### Electrochemical Properties

The redox potentials of **[Ir1]Cl–[Ir5]Cl** were experimentally ascertained by cyclic voltammetry (CV) in deoxygenated CH_3_CN solutions (5 × 10^–4^ M), in order to establish the oxidative and reductive abilities of the corresponding ground and excited states, as well as the redox stability of our complexes. Potentials are referred to the ferrocenium/ferrocene (Fc^+^/Fc) couple.

**Figure 6:**
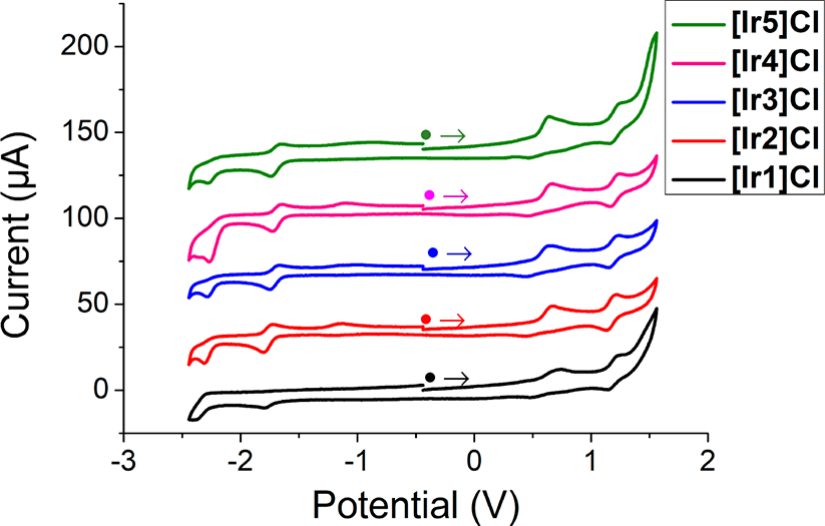
Cyclic voltammograms of complexes **[Ir1]Cl**–**[Ir5]Cl** in acetonitrile solution (5 × 10^–4^ M), using 0.1 M [*n*Bu_4_N][PF_6_] as supporting electrolyte and recorded with scan rate of 0.10 V·s^–1^.

The cyclic voltammograms (CV) of these compounds are presented in Figure 6. The anodic region of every CV shows two peaks: (a) an irreversible peak between +0.56 and +0.63 V (Table 3 and Figure 6) attributed to the oxidation of the chloride counteranion (2 Cl^–^ → Cl_2_ + 2 e^–^) and (b) a reversible one-electron oxidation peak in the range +1.19 to +1.22 V, ascribed to an oxidation process affecting the Ir(III) center along with the difluorophenyl rings of C^N ligands,^28^ as disclosed by the topology of the respective HOMO.

**Table 3:** Redox Potentials for Complexes **[Ir1]Cl**–**[Ir5]Cl** Referenced to Fc^+^/Fc and recorded by CV in Acetonitrile Solution^a^

complex	*E*_1/2_^ox1^ (V)	*E*_1/2_^ox2^ (V)	*E*_1/2_^red1^ (V)	*E*_1/2_^red2^ (V)	Δ*E*_1/2_ (V)^b^	*E*_1/2_(Ir^IV^/Ir^III^*)^d^	*E*_1/2_(Ir^III^*/Ir^II^)^d^
**[1]PF**_**6**_^c^	–	+0.87	–1.78 (qr)	–	2.65	–1.19	+0.28
**[2]PF**_**6**_^c^	–	+1.22	–1.65	–	2.87	–1.10	+0.67
**[Ir1]Cl**	+0.63 (ir)	+1.22 (qr)	–1.79 (ir)	–2.33 (ir)	3.01	–1.16	+0.59
**[Ir2]Cl**	+0.58 (ir)	+1.19 (qr)	–1.76 (qr)	–2.27 (ir)	2.95	–1.11	+0.54
**[Ir3]Cl**	+0.56 (ir)	+1.21 (qr)	–1.69 (qr)	–2.22 (ir)	2.90	–1.07	+0.59
**[Ir4]Cl**	+0.59 (ir)	+1.22 (qr)	–1.67 (qr)	–2.21 (ir)	2.89	–1.06	+0.61
**[Ir5]Cl**	+0.59 (ir)	+1.22 (qr)	–1.69 (qr)	–2.23 (ir)	2.91	–1.05	+0.58

a Data for **[1]PF**_**6**_ and **[2]PF**_**6**_ reported by McCusker^53^ and Ko,^54^ respectively. Voltammograms recorded in acetonitrile solution (5 × 10^–4^ M), using 0.1 M [*n*Bu_4_N][PF_6_] as supporting electrolyte with scan rate of 0.10 V·s^–1^ and referenced to Fc^+^/Fc (qr = quasi-reversible, ir = irreversible)

b Δ*E*_1/2_ = *E*_1/2_^ox2^ – *E*_1/2_^red1^.

c Data for **[1]PF**_**6**_ and **[2]PF**_**6**_ are given in acetonitrile vs Fc^+^/Fc (calculated from the original works using the equation: *V*(Fc^+^/Fc) = *V*(SCE) – 0.404).

d *E*_1/2_(Ir^IV^/Ir^III*^) and *E*_1/2_(Ir^III*^/Ir^II^) are calculated as explained in Table S4.

**Figure 7:**
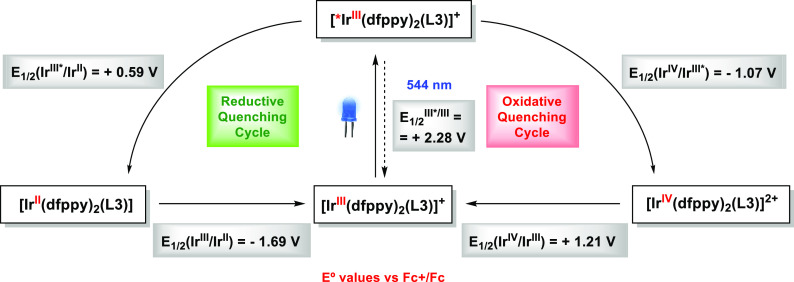
Latimer diagram for **[Ir3]Cl**, with redox potentials determined by CV and the emission energy calculated from the photoluminescence spectrum. The redox potentials for **[Ir3]**^**+**^ and its excited state [Ir3*]^**+**^ are given in V versus Fc^+^/Fc. *E*_1/2_(Ir^IV^/Ir^III^*) = *E*_1/2_(Ir^IV^/Ir^III^) – *E*_1/2_(Ir^III^*/Ir^III^) and *E*_1/2_(Ir^III^*/Ir^II^) = *E*_1/2_(Ir^III^/Ir^II^) + *E*_1/2_(Ir^III^*/Ir^III^). All the potential values are given as reduction potentials regardless the sense of the arrows for the quenching cycles.

In the cathodic region, **[Ir1]Cl** exhibits two irreversible peaks (*E*_1/2_^red1^ = −1.79 V, *E*_1/2_^red2^ = −2.33 V). Nonetheless, complexes **[Ir2]Cl**–**[Ir5]Cl** display one pseudoreversible one-electron peak and one irreversible one-electron peak in the ranges from −1.67 to −1.76 V (*E*_1/2_^red1^) and from −2.21 to −2.27 V (*E*_1/2_^red2^), respectively. These waves are attributed to stepwise reductions centered in the respective N^N′ ligands, as suggested by the topology of the calculated LUMO for these compounds. Interestingly, the pseudoreversible nature of *E*_1/2_^red1^ observed for **[Ir2]Cl**–**[Ir5]Cl**, compared to the irreversible character of *E*_1/2_^red1^ obtained for **[Ir1]Cl**, underlines the stabilizing effect of the alkyl groups attached to the N^N′ ligands on the redox behavior of these dyes. Moreover, a low intensity irreversible wave is observed for complexes **[Ir2]Cl**–**[Ir4]Cl** between −1.00 and −1.11. This peak is imputed to the oxidation of a species formed *in situ* by chemical decomposition during the CV experiment, as it can only be seen in the return scan. The experimental electrochemical band gaps have been calculated as the difference between *E*_1/2_^ox2^ and *E*_1/2_^red1^. They are in a very narrow range for complexes **[Ir1]Cl**–**[Ir5]Cl** and are very similar to the value reported for **[2]PF**_**6**_, although are higher than the respective value for **[1]PF**_**6**_ in agreement with the trends predicted theoretically for the HOMO–LUMO band gaps. Paradoxically, the excited states of this type of Ir(III) derivatives exhibit a versatile and outstanding redox behavior.^50−52^ Indeed, our dyes show a high excited-state redox power as oxidants, *E*_1/2_(Ir^III*^/Ir^II^) ranges from +0.54 to +0.61 V, and also as reductants, *E*_1/2_(Ir^IV^/Ir^III*^) ranges from −1.16 to −1.05 V (Figure 7 and Table S4), and they are meaningfully better excited-state oxidants than the standard photosensitizer **[1](PF**_**6**_**)** (*E*_1/2_(Ir^III*^/Ir^II^) = +0.28 V).^53^ These facts underscore their potential as photocatalysts in single electron transfer (SET) processes.

### Photocatalytic Activity in the Oxidation of Heterocycles

The new iridium complexes were tested as photocatalysts in the dehydrogenation of different partially saturated heterocycles (indolines, quinolines, isoquinolines, etc.). First, we chose indoline (**1a**) as the model substrate and irradiated it with blue light (460 nm) in the presence of **[Ir1]Cl** (1 mol %) under O_2_ (1 atm, pure oxygen balloon) at room temperature for 24 h, using three different solvents, acetonitrile, dichloromethane, and ethanol.

Thus, we could determine that acetonitrile is the best solvent choice, since it provides a quantitative yield (>99%) for indole (**2a**), whereas lower yields were obtained using dichloromethane and ethanol (Table 4) under analogous conditions. It is noteworthy that the transformation is selective for **2a**, since no overoxidation products such as isatin were observed.^55,56^ Then, we performed a catalyst screening for the photooxidation of indoline (**1a**) using acetonitrile as solvent and a catalyst loading of 0.1% for **[Ir1]Cl**–**[Ir5]Cl** and also for **[1]Cl** and **[2]Cl**. Consequently, we found out that **[Ir3]Cl** is the most efficient catalyst for the oxidative dehydrogenation of indoline (**1a**), whereas **[Ir1]Cl** provided a very low yield for **2a** (entries 1 and 3, Table 5). We tentatively explain the poor yield obtained with the nonalkylated luminophore **[Ir1]Cl** owing to the irreversible nature of its reduction [Ir^III^] → [Ir^II^] and its short excited-state lifetime, as seen in Figure 6 and discussed in the Mechanism section.

**Table 4:**
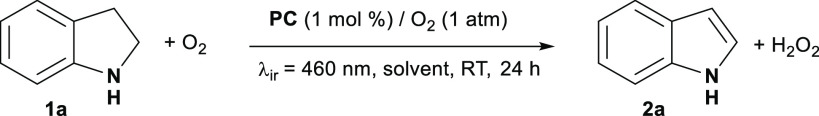
Solvent Screening in the Photooxidation of Indoline **1a**^a^

entry	solvent	yield (%)
1	**CH**_**3**_**CN**	100
2	**CH**_**2**_**Cl**_**2**_	65
3	**EtOH**	48

a Reaction conditions: Indoline **1a** (10 mM), **PC** (**[Ir1]Cl**, 1 mol %), solvent (0.5 mL), O_2_ (balloon, 1 atm), blue light (LED, λ_ir_ = 460 nm, 24 W), room temperature, for 24 h. Yields of **2a** were experimentally determined by ^1^H NMR integration of the corresponding reaction crudes. The yield values were calculated as the mean of three independent experiments.

**Table 5:**
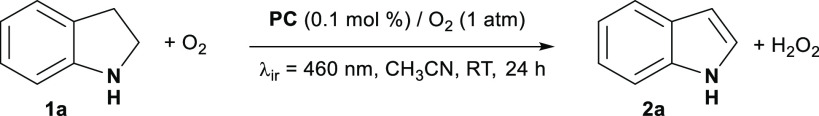
Photocatalysts Screening in the Photooxidation of Indoline **1a**^a^

entry	complex	yield (%)
1	**[1]Cl**	54
2	**[2]Cl**	55
3	**[Ir1]Cl**	20
4	**[Ir2]Cl**	42
5	**[Ir3]Cl**	62
6	**[Ir4]Cl**	58
7	**[Ir5]Cl**	57

a Reaction conditions: Indoline **1a** (10 mM), **PC** (0.1 mol %), acetonitrile (0.5 mL), O_2_ (balloon, 1 atm), blue light (LED, λ_ir_ = 460 nm, 24 W), room temperature, for 24 h. Yields of **2a** were experimentally determined by ^1^H NMR integration of the corresponding reaction crudes. The yield values were calculated as the mean of three independent experiments.

By contrast, it is worth remarking that the alkylated derivatives, **[Ir3]Cl**–**[Ir5]Cl**, provide better yields than the standard PSs, **[1]Cl** and **[2]Cl**, as a result of a balanced combination of favorable photophysical and electrochemical properties.

Next, we performed a collection of control experiments to verify the photocatalytic essence of this transformation and the role of O_2_. Indeed, we realized that in the absence of light, PC, or O_2_ (N_2_ atmosphere), the reaction did not proceed or was dramatically impeded, and thus we concluded that this transformation is light-driven in the presence of a PC and that oxygen is involved in the oxidation (Table 6). It is worth mentioning that the detection of a small percentage of **2a** under a N_2_ atmosphere (entry 4, Table 6) could be due to the presence of O_2_ traces in the solvent (incomplete deoxygenation). Moreover, we carried out additional control experiments in the presence of DABCO (^1^O_2_ quencher),^57^ TEMPO (radical scavenger),^58^ and 1,4-benzoquinone (BQ, O_2_^•–^ scavenger)^56,59^ to elucidate the actual oxidant. The presence of DABCO decreases the yield slightly (84%), while TEMPO and BQ cause a dramatic and significant drop of the yield, respectively. These results suggest that superoxide has a major contribution in this reaction, while singlet oxygen plays just a minor role (Table 6).

**Table 6:**
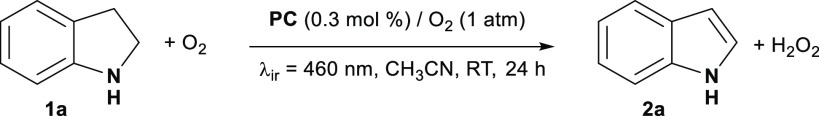
Control Experiments for the Photooxidation of Indoline **1a**^a^

entry	conditions	yield (%)
1	PC, O_2_, light	100
2	PC, O_2_, no light	0
3	no PC, O_2_, light	0
4	PC, N_2_, light	10
5	PC, O_2_, light, DABCO^b^	84
6	PC, O_2_, light, TEMPO^c^	15
7	PC, O_2_, light, BQ^d^	45

a Reaction conditions: Indoline **1a** (10 mM), PC (**[Ir3]Cl**, 0.3 mol %), CH_3_CN (0.5 mL) at room temperature, under a saturated atmosphere of either O_2_ or N_2_ (1 atm), and under irradiation with blue light (LED, λ_ir_ = 460 nm, 24 W) during 24 h in a septum-capped tube. Yields of **2a** were determined by ^1^H NMR integration of the corresponding reaction crudes.

b DABCO (3 equiv).

c TEMPO (3 equiv).

d BQ (3 equiv). The yield values were calculated as the mean of three independent experiments.

Then, we tested the substrate scope using the optimized conditions on a variety of indolines bearing different functional groups (Table 7). Most of the desired indoles were obtained in high yields and with excellent selectivities. However, the oxidation of 1-acetyl-5-bromoindoline (**1f**) was ineffective. This failure is likely due to the electron-withdrawing and steric effects attributed to the *N*-acetyl group, which inhibit the oxidation step.^8,16,18^ Indeed, according to our general mechanistic proposal, we presume that the reductive quenching of the triplet excited state of the PC, ^3^[Ir^III^]*, in the presence of **1f** would give rise to an unstable radical cation intermediate due to the remarkable electron-withdrawing effect attributed to the formyl substituent on the N atom. Moreover, the oxidative dehydrogenation of 5-nitroindoline (**1c**) and 6-nitroindoline (**1g**) were also precluded (0 and 20% of respective indoles), which is likely related to the strong electron-withdrawing ability of the −NO_2_ group.^18^ Indeed, it is well-known that electron-poor nitro-aromatic substrates can undergo a photoinduced electron donation from the triplet excited state of different photosensitizers, which competes with the photoinduced reductive quenching proposed as one of the steps in the mechanism of this reaction.^60,61^

**Table 7:**
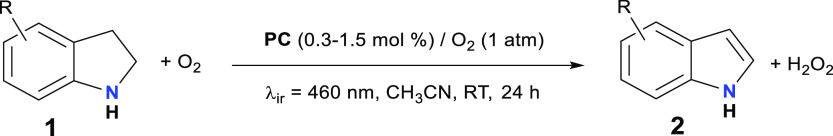

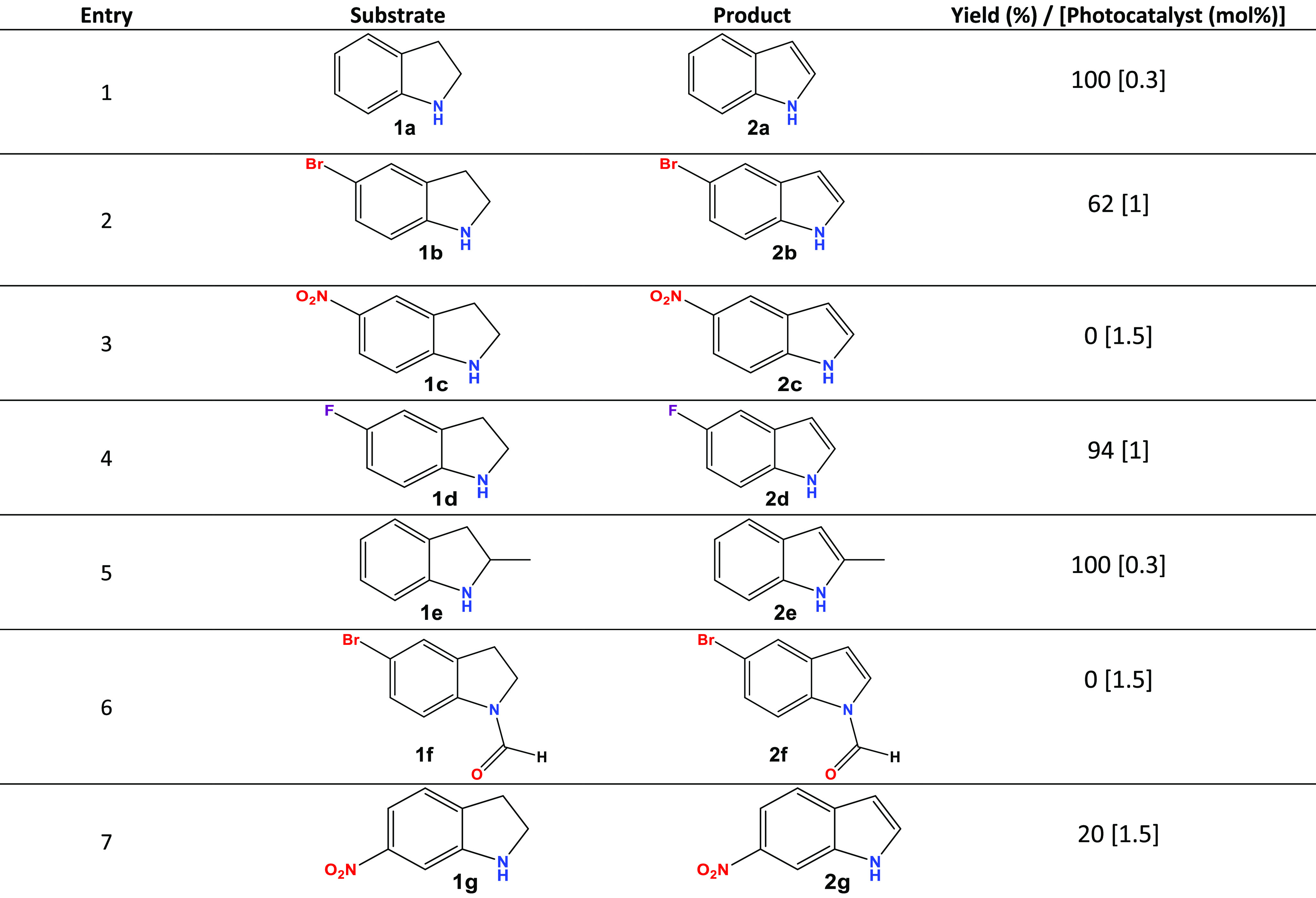
Substrate Scope for the Photooxidation of Indolines^a^

a Reaction conditions: Indoline (10 mM), PC (**[Ir3]Cl**; 0.3–1.5 mol %), CH_3_CN (0.5 mL) at room temperature, under a saturated atmosphere of O_2_ (1 atm) and under irradiation with blue light (LED, λ_ir_ = 460 nm, 24 W) during 24 h in a septum-capped tube. Yields were determined by ^1^H NMR integration of the corresponding reaction crudes. The yield values were calculated as the mean of three independent experiments.

To validate the applicability of this protocol, we decided to scale the reaction up to 1 g of indoline (**1a**) in the presence of **[Ir3]Cl** (0.3 mol %). Thus, it was possible to obtain **2a** in 95% yield by increasing the reaction time from 24 to 75 h. (see SI and ^1^H and ^13^C NMR of isolated products and characterization in Figures S19–S32).

Next, we assayed the stepwise oxidative dehydrogenation of 1,2,3,4-tetrahydroquinolines to produce the respective quinolines. First, we selected 1,2,3,4-tetrahydroquinoline (THQ, **3a**) as the model substrate and applied the standard conditions using **[Ir1]Cl** as the photocatalyst (1 mol %) for 24 h in three different solvents, that is, acetonitrile, dichloromethane, and ethanol (Table 8).

**Table 8:**
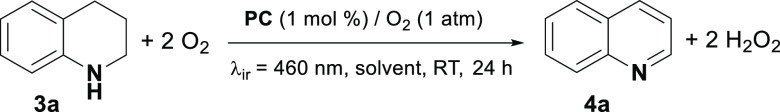
Solvent Screening in the Photooxidation of 1,2,3,4-Tetrahydroquinoline **3a**^a^

entry	solvent	yield (%)
1	CH_3_CN	20
2	CH_2_Cl_2_	13
3	EtOH	7

a Reaction conditions: 1,2,3,4-Tetrahydroquinoline **3a** (10 mM), PC (**[Ir1]Cl**, 1 mol %), solvent (0.5 mL), O_2_ (balloon, 1 atm), blue light (LED, λ_ir_ = 460 nm, 24 W), room temperature for 24 h. Yields of **4a** were experimentally determined by ^1^H NMR integration of the corresponding reaction crudes. The yield values were calculated as the mean of three independent experiments.

Again, acetonitrile provided the best yield for quinoline, **4a**, (20%) and was chosen as the solvent for additional experiments. Partial dehydrogenation products such as 3,4-dihydroquinoline were not detected, making this protocol selective.^62^ A PCs screening including complexes **[Ir1]Cl**–**[Ir5]Cl** and also **[1]Cl** and **[2]Cl** was performed using a catalyst loading of 1 mol %. Unlike **[Ir1]Cl**, the functionalized PCs, **[Ir2]Cl**–**[Ir5]Cl**, promoted full conversions of **3a** to **4a** under these conditions. Hence, in order to discriminate the most active PC, we examined the photocatalytic activity of these complexes one more time, decreasing the catalyst loading down to 0.1 mol % (Table 9). In short, **[Ir4]Cl** turned out to be the most efficient PC (70% yield of **4a**), and again, the nonalkylated complex, **[Ir1]Cl**, was by far the less efficient PC. Besides, complexes **[Ir3]Cl**–**[Ir5]Cl** exhibited better performances than the archetypal photosensitizer **[1]Cl**, and **[Ir4]Cl**–**[Ir5]Cl** are even more active than the fluorinated standard PS **[2]Cl**. We theorize that the good performance of **[Ir5]Cl**, despite its low Φ_PL_, could be ascribed to its better absorptivity in the visible range.

**Table 9:**
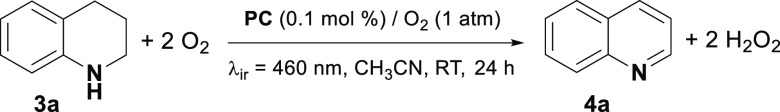
Photocatalysts Screening in the Photooxidation of 1,2,3,4-Tetrahydroquinoline **3a**^a^

entry	complex	yield (%)
1	**[1]Cl**	45
2	**[2]Cl**	62
3	**[Ir1]Cl**	0
4	**[Ir2]Cl**	27
5	**[Ir3]Cl**	56
6	**[Ir4]Cl**	70
7	**[Ir5]Cl**	68

a Reaction conditions: 1,2,3,4-Tetrahydroquinoline **3a** (10 mM), PC (0.1 mol %), acetonitrile (0.5 mL), O_2_ (balloon, 1 atm), blue light (LED, λ_ir_ = 460 nm, 24 W), room temperature for 24 h. Yields of **4a** were experimentally determined from ^1^H NMR integration of the corresponding reaction crudes. The yield values were calculated as the mean of three independent experiments.

The usual control experiments were done to gain insight into the mechanism of this transformation. In particular, we observed no conversion without light or PC as well as a drastic decrease in the yield in the absence of O_2_ (4% of **4a**, under a N_2_ atmosphere) (Table 10). The use of the ROS scavengers DABCO, TEMPO, and BQ revealed similar behaviors to those established for the photooxidation of indoline, that is, a slight drop in the yield of **4a** in the presence of DABCO (87%), but a severe inhibition of the transformation in the presence of TEMPO (7%) and BQ (17%) relative to the standard conditions (entries 1 and 5–7 in Table 10). In conclusion, we propose that both singlet oxygen and superoxide take part in the dehydrogenation reaction of 1,2,3,4-tetrahydroquinoline, although the main role would correspond to the radical anion superoxide (O_2_^•–^). To gain additional insight into the reaction mechanism, we performed emission quenching Stern–Volmer experiments. Thus, we could determine that phosphorescence of **[Ir4]Cl** was strongly quenched in the presence of increasing concentrations of **3a** under nitrogen, and consequently we proved that reductive quenching can be rationally proposed as the first step in the mechanism of this transformation. In other words, we concluded that **3a** can be efficiently oxidized by [Ir4]^*+^ with a quenching constant, *K*_sv_ = 29.728 × 10^3^ M^–1^.^63−65^ However, we also demonstrated that the emission of **[Ir4]Cl** is quenched upon exposure to open air (Figure S47). Hence, oxidative quenching of [Ir4]^*+^ mediated by O_2_ can operate as the first step in the mechanism of this reaction as well. See a detailed discussion below.

**Table 10:**
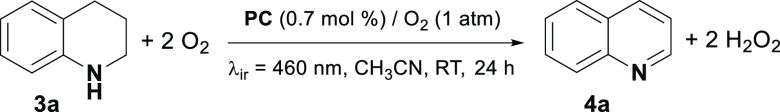
Control Experiments for the Photooxidation of 1,2,3,4-Tetrahydroquinoline **3a**^a^

entry	conditions	yield (%)
1	PC, O_2_, light	100
2	PC, O_2_, no light	0
3	no PC, O_2_, light	0
4	PC, N_2_, light	4
5	PC, O_2_, light, DABCO^b^	86
6	PC, O_2_, light, TEMPO^c^	7
7	PC, O_2_, light, BQ^d^	17

a Reaction conditions: 1,2,3,4-Tetrahydroquinoline **3a** (10 mM), PC (**[Ir4]Cl**; 0.7 mol %), CH_3_CN (0.5 mL) at room temperature, under a saturated atmosphere of either O_2_ or N_2_ (1 atm) and under irradiation with blue light (LED, λ_ir_= 460 nm, 24 W) during 24 h in a septum-capped tube. Yields of **4a** were determined by ^1^H NMR integration of the corresponding reaction crudes.

b DABCO (3 equiv).

c TEMPO (3 equiv).

d 1,4-Benzoquinone (3 equiv). The yield values were calculated as the mean of three independent experiments.

To complete this study, we extended the above-mentioned protocol to a selection of tetrahydroquinolines and analogues, such as 1,2,3,4-tetrahydroisoquinoline, 9,10-dihydroacridine and several 1,2,3,4-tetrahydroquinoxalines (Table 11).

**Table 11:**
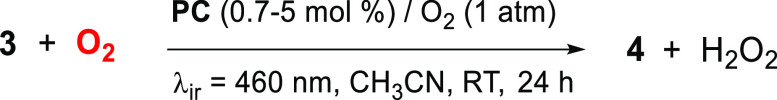

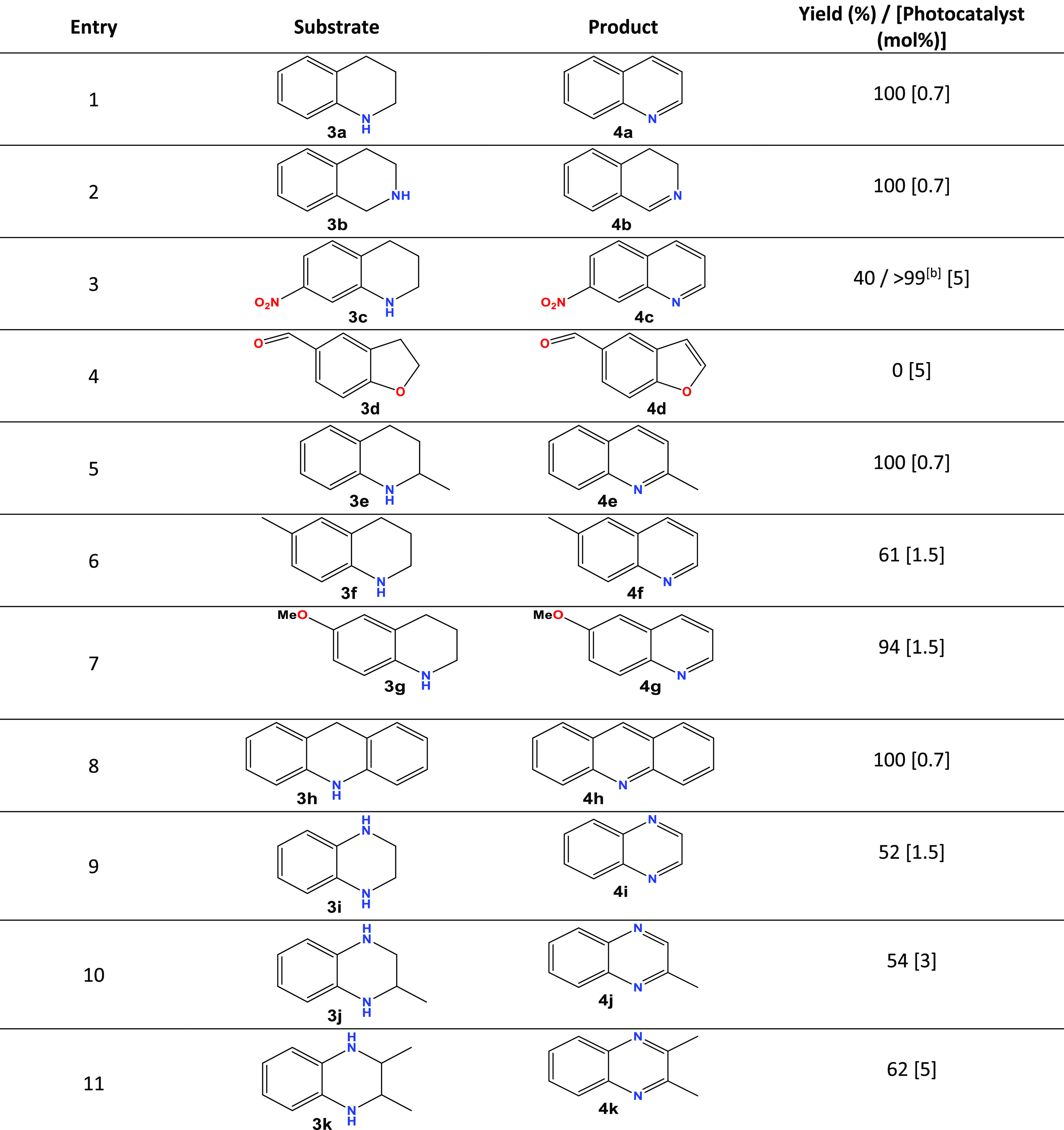
Substrate Scope for the Photooxidation of Tetrahydroquinolines^a^

a Reaction conditions: Substrate (10 mM), PC (**[Ir4]Cl**; 0.7–5 mol %), CH_3_CN (0.5 mL) at room temperature, under a saturated atmosphere of O_2_ (1 atm) and under irradiation with blue light (LED, λ_ir_ = 460 nm, 24 W) during 24 h in a septum-capped tube. Yields were determined by ^1^H NMR integration of the corresponding reaction crudes.

b Reaction time of 48 h. The yield values were calculated as the mean of three independent experiments.

In general, we obtained high yields and excellent selectivity for most of the expected products (**4b**, **4c**, and **4e**–**4h**). In a previous photocatalytic protocol, Bahnemann et al. obtained a mixture between the partially dehydrogenated product **4b** and the fully dehydrogenated product, when using the tetrahydroisoquinoline **3b**.^18^ However, the yields for the quinoxalines, **4i**–**4k**, and 6-methyl-quinoline, **4f**, were only moderate, in the range between 52 and 62%. On the other hand, 2,3-dihydrobenzofuran-5-carboxaldehyde (**3d**) was not oxidized to its dehydrogenated derivative. It is noteworthy that the oxidation of 7-nitro-1,2,3,4-tetrahydroquinoline (**3c**) was achieved albeit with a low yield, since, as aforementioned, the nitro substituent usually behaves as a quencher for the excited state of PCs. Moreover, the yield for **3c** could be improved by prolonging the reaction time and increasing the catalyst loading (>99% yield, with 5 mol % PC, 48 h).

After this, we successfully scaled our methodology up to 1 g of **3a** in the presence of **[Ir4]Cl** (0.7 mol %) to obtain **4a** with a yield of 88%, albeit it was necessary to extend the reaction time from 24 to 75 h (see ^1^H and ^13^C NMR spectra of isolated products in Figures S42 and S43).

### Mechanism

Based on the experimental results summarized in Table 12 along with the bibliographic background,^17^ we propose a dual mechanism for the aerobic photooxidative dehydrogenation of 1,2,3,4-tetrahydroquinoline based on both a reductive quenching cycle (pathway A) and simultaneously on an oxidative quenching cycle (pathway B). In both cases, the reaction is mediated by the radical anion superoxide (O_2_^•–^), and we postulate that both mechanisms could operate concurrently (Figure 9).

**Table 12:** Experimental Evidences Supporting the Reductive and Oxidative Quenching Cycles and the Participation of O_2_^•–^ in the Afore-Mentioned Photocatalytic Reactions

evidence	experiment
strong inhibition of photocatalytic oxidation in the presence of TEMPO and BQ (radical and O_2_^•–^ scavengers)	control experiments performed in Tables 6 and 10
low photocatalytic activity obtained for **[Ir1]Cl**, due to irreversible reductive quenching	screening of photocatalysts (Tables 5 and 9) and redox potentials (Table 3)
low dehydrogenation for **1c**, **1g**, and **3c**, due to the presence of – NO_2_ groups which induce oxidative quenching on PS and inhibit the photocatalytic quenching steps	substrate scope experiments (Tables 7 and 11)
suitable redox potentials for sustaining both a reductive quenching cycle and an oxidative quenching cycle	see text in this section and Tables 3 and S4
detection of H_2_O_2_	^1^H NMR of crudes and peroxide test sticks
evidence of both reductive quenching of *PC in the presence of THQ and oxidative quenching in the presence of O_2_	Stern–Volmer experiments in Figure 8 and Figure S47

**Figure 8:**
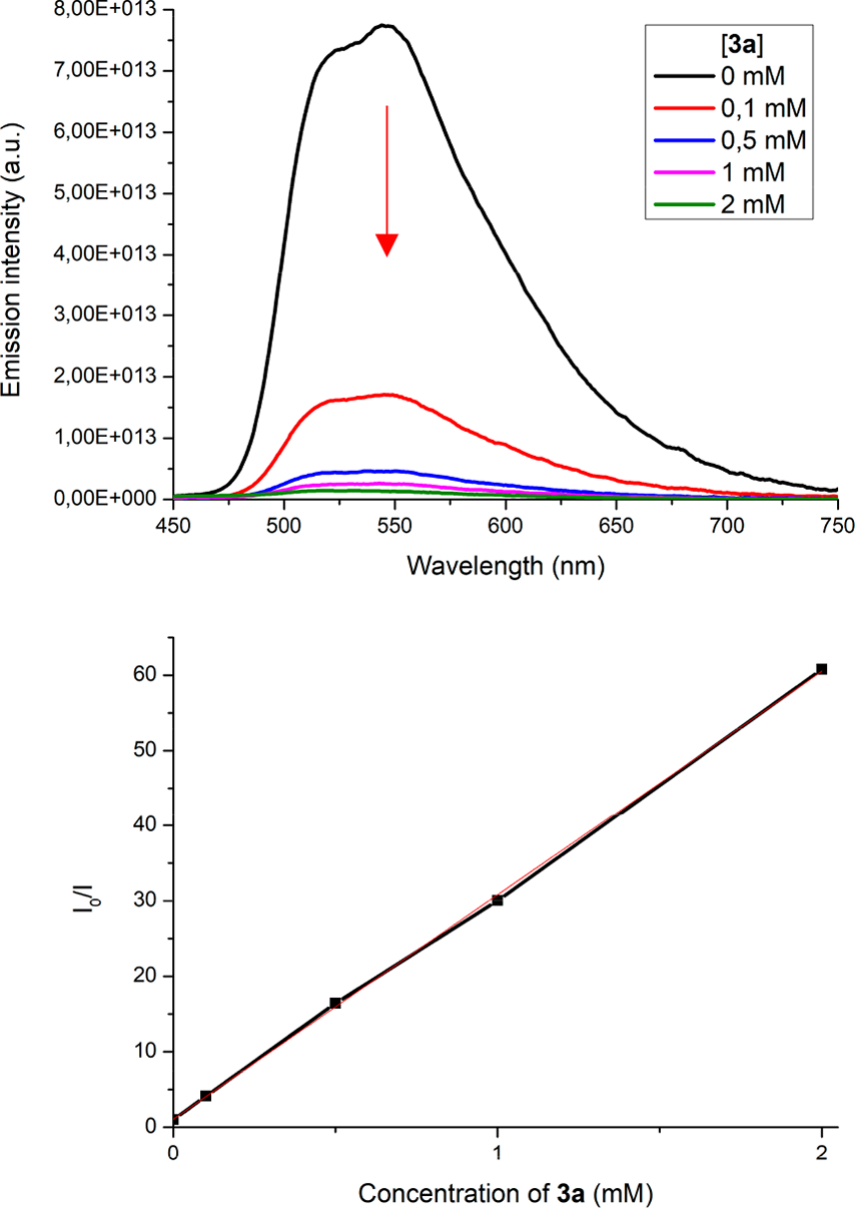
Stern–Volmer quenching experiments. (a) Emission quenching of **[Ir4]Cl** (0.07 mM in CH_3_CN, 25 °C) upon incremental addition of substrate **3a** (0.1–2 mM) under N_2_ and λ_ir_ = 405 nm. (b) Stern–Volmer quenching plot, where *I*_0_ = PL intensity of **[Ir4]Cl** at [**3a**] = 0 mM; *I* = PL intensity of **[Ir4]Cl** at different [**3a**]; *I*_0_/*I* = 29.728 × [**3a**] + 1.0544; *R*^2^ = 0.9996.

**Figure 9:**
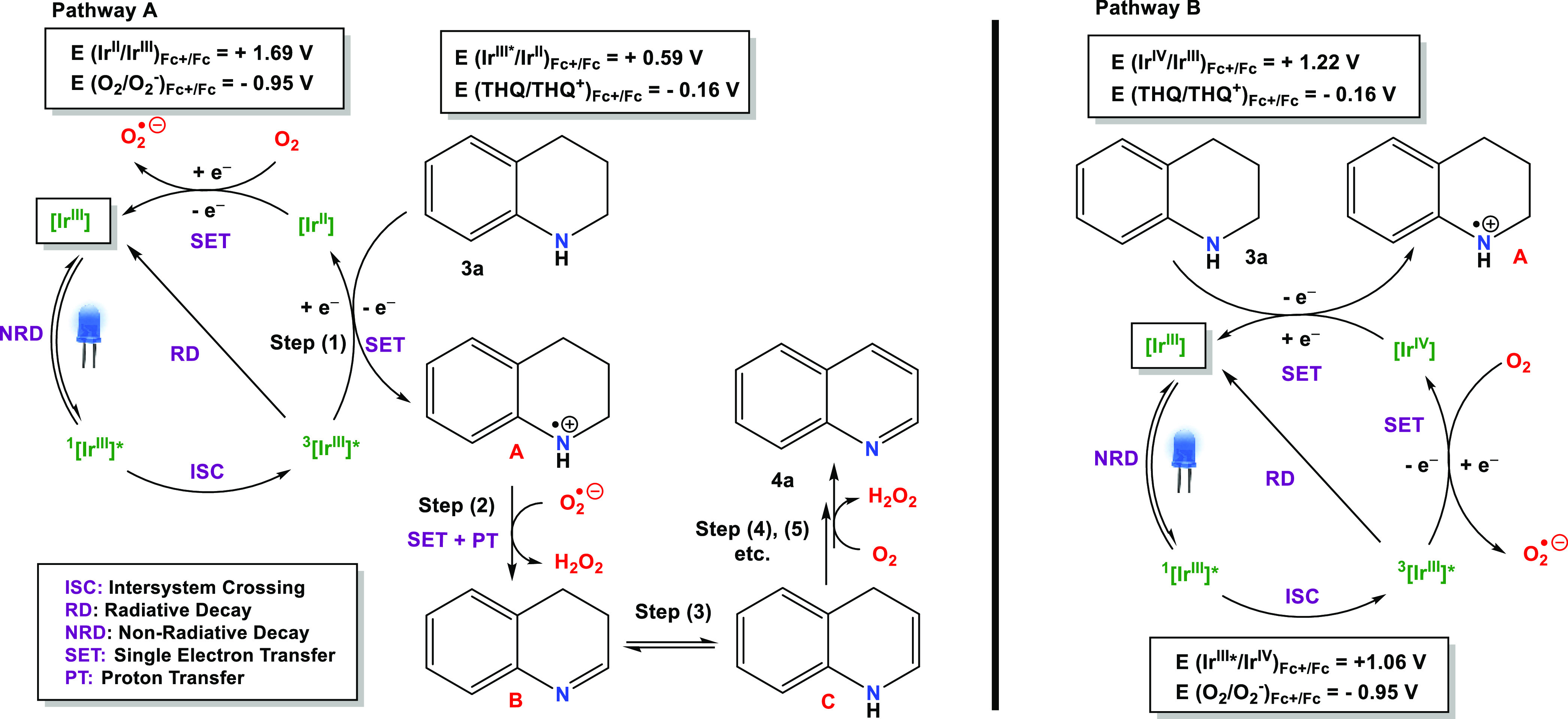
Pathways A and B for the oxidative dehydrogenation of **3a** in the presence of the new Ir(III) PCs. Steps (2), (3) and (4), (5) etc. from species **A** are common for both pathways.

#### Pathway A

First, the model Ir(III) photosensitizer, **[Ir3]Cl**, is promoted to the singlet excited state under irradiation with blue light and then is converted to the respective triplet excited state through intersystem crossing. This species, ^**3**^**[Ir**^**III**^]*, exhibits a high oxidation ability and therefore is capable of generating the radical cation intermediate species **A** (THQ^+^), through a SET, which entails a reductive quenching of the excited state. The redox potential of the couple THQ/THQ^+^ was determined by CV, *E*(THQ/THQ^+^) = −0.16 V vs Fc^+^/Fc (Figure S24) and compared to *E*(Ir^III*^/Ir^II^) = +0.59 V to demonstrate the feasibility of this step. Concurrently, the reduced form of the PC, **[Ir**^**II**^**]**, is formed, and then, **[Ir**^**II**^**]** reduces O_2_ to produce O_2_^•–^. Next, two protons and one additional electron are transferred from intermediate **A** to O_2_^•–^, resulting in intermediate **B** plus one molecule of hydrogen peroxide (H_2_O_2_). It is noteworthy that H_2_O_2_ has been detected by both ^1^H NMR and using Quantofix peroxide sticks in the crude solutions of photocatalytic assays (see SI). Subsequently, imine-enamine tautomerization facilitates the second oxidation process, yielding the aromatic heterocycle.

#### Pathway B

Alternatively, ^**3**^**[Ir**^**III**^]* can undergo oxidative quenching upon reaction with O_2_ to produce O_2_^•–^ and concomitantly the oxidized intermediate **[Ir**^**IV**^**]** (Figure 9). Both the emission quenching of **[Ir4]Cl** in the presence of O_2_ and the corresponding redox potentials, E(Ir^III*^/Ir^IV^) = 1.06 V and E(O_2_/O_2_^•–^) = −0.95 V versus Fc^+^/Fc support this step. Subsequently, **[Ir**^**IV**^**]** oxidizes THQ to generate the species **A** (THQ^+^), returning to its ground state **[Ir**^**III**^**]**. Then, O_2_^•–^ and species **A** react to give **B** and **C**, as explained above for pathway A. A similar mechanism could operate for the photocatalytic aerobic dehydrogenation of indolines, etc.

## Conclusions

In conclusion, we have designed and prepared a new family of Ir(III) photosensitizers of the general formula [Ir(C^N)_2_(N^N′)]Cl, where C^N = 2-(2,4-difluorophenyl)pyridinate and N^N′ = 2-(2′-pyridyl)benzimidazole (**L1**) or its N-alkylated derivatives **L2**-**L5**. We have ascertained that these complexes are notably stable under irradiation with blue light for a period of 24 h. Moreover, we have demonstrated that they absorb weakly in the visible light region and can be excited with blue light. Indeed, all of them are emissive in the range between 522 and 546 nm (λ_ex_ = 405 nm). In particular, the N-functionalized derivatives, **[Ir2]Cl**–**[Ir5]Cl**, exhibit moderate or high PLQYs (9–63%) and very long excited-state lifetimes (1012–2066 ns). On the contrary, the nonalkylated compound, **[Ir1]Cl**, features an excellent PLQY (78%) but a very short excited-state lifetime (59 ns). This divergent behavior suggests that the N–H group speeds up the radiative deactivation of the excited state for **[Ir1]Cl**, by stabilization of the ground state through hydrogen bonds with counterion/solvent molecules, whereas the replacement of the N–H with apolar *N*-alkyl groups prevents this effect on the ground state and lengthens the lifetime of the respective excited states in acetonitrile. Regarding their electrochemical properties, all the Ir complexes display a similar redox behavior, with electrochemical band-gaps higher than that determined for the standard photosensitizer [Ir(ppy)_2_(bpy)]PF_6_, **[1]PF**_**6**_. This is due to the strong stabilization of the HOMO, associated with the presence of electro-withdrawing −F atoms in the C^N ligands of our PS, as revealed by theoretical calculations. Nevertheless, **[Ir1]Cl** features an irreversible *E*_1/2_^red1^ in contrast to the reversible *E*_1/2_^red1^ of its derivatives.

Upon excitation with blue light, these compounds exhibit highly efficient and selective photocatalytic activities in the preparation of a wide variety of aromatic N-heterocyclic products through oxidative dehydrogenation of partially saturated substrates such as different indolines, 1,2,3,4-tetrahydroquinolines, 1,2,3,4-tetrahydroisoquinoline, 9,10-dihydroacridine, and 1,2,3,4-tetrahydroquinoxalines. More specifically, the performance of the N-alkylated derivatives is better than that of **[Ir1]Cl** in these transformations, which seems to be linked to either the irreversible *E*_1/2_^red1^ of **[Ir1]Cl** compared to the reversible *E*_1/2_^red1^ of **[Ir2]Cl**-**[Ir5]Cl** or the low excited-state lifetime of **[Ir1]Cl**. We have proved the efficiency of this methodology on a gram scale for the synthesis of **2a** and **4a**. It is worth mentioning that this protocol satisfies most of the requirements of green chemistry, since it makes use of O_2_ as a green oxidant, acetonitrile as a low boiling point solvent, visible light as the energy source, and very low PC loadings.^66^ Furthermore, we propose that these Ir-photosensitized transformations occur through a dual mechanism based on both a reductive quenching cycle (pathway A) and an oxidative quenching cycle (pathway B) which operate simultaneously and are mediated by the radical anion superoxide (O_2_^•–^).

To summarize, we have shown that the easy N-alkylation of 2-(2′-pyridyl)benzimidazole affords ligands suitable for the assembly of Ir(III) photosensitizers, **[Ir2]Cl**–**[Ir5]Cl**, which feature ideal properties to be used in photoredox catalysis. Indeed, these PSs exhibit highly efficient and selective photocatalytic activities in the preparation of a wide variety of N-heterocyclic products through oxidative dehydrogenation of partially saturated substrates. The above-mentioned results provide insights and tools for the rational design of efficient photocatalysts.

## Experimental Section

### General Information and Procedures

All synthetic manipulations were carried out under an atmosphere of dry, oxygen-free nitrogen using standard Schlenk techniques. The solvents were dried and distilled under nitrogen atmosphere before use. Elemental analyses were performed with a Thermo Fisher Scientific EA Flash 2000 elemental microanalyzer. IR spectra were recorded on a Jasco FT/IR-4200 spectrophotometer (4000–400 cm^–1^ range) with single reflection ATR measuring attachment. UV–vis absorption was measured in an Evolution 300 UV–vis double beam spectrophotometer (Thermo Scientific). Fluorescence steady-state and lifetime measurements were performed in a FLS980 (Edinburg Instruments) fluorimeter with xenon arc lamp 450W and TCSPC laser, respectively. Quantum yield was determined by using in a FLS980 (Edinburg Instruments) with xenon arc lamp 450W and Red PMT Sphere as detector. HR-ESI(+) mass spectra (position of the peaks in Da) were recorded with an Agilent LC-MS system (1260 Infinity LC/6545 Q-TOF MS spectrometer) using DCM/DMSO (4:1) as the sample solvent and (0.1%) aqueous HCOOH/MeOH as the mobile phase. The experimental *m*/*z* values are expressed in Da compared with the *m*/*z* values for monoisotopic fragments. NMR samples were prepared by dissolving the suitable amount of compound in 0.5 mL of the respective deuterated solvent, and the spectra were recorded at 298 K on a Varian Unity Inova-400 (399.94 MHz for ^1^H; 376.29 MHz for ^19^F; 100.6 MHz for ^13^C). Typically, ^1^H NMR spectra were acquired with 32 scans into 32,000 data points over a spectral width of 16 ppm. ^1^H and ^13^C{^1^H} chemical shifts were internally referenced to TMS via the residual ^1^H and ^13^C signals of DMSO-*d*_6_ (δ = 2.50 ppm and δ = 39.52 ppm), CD_3_CN (δ = 1.94 ppm and δ = 118.69 (−CN) and 1.39 (−CD_3_) ppm) and CDCl_3_ (δ = 7.26 ppm and δ = 77.16 ppm), according to the values reported by Fulmer et al.^1^ Chemical shift values (δ) are reported in ppm and coupling constants (*J*) in hertz. The splitting of proton resonances in the reported ^1^H NMR data is defined as s = singlet, d = doublet, t = triplet, q = quartet, m = multiplet, bs = broad singlet. 2D NMR spectra such as ^1^H–^1^H gCOSY, ^1^H–^1^H NOESY, ^1^H–^13^C gHSQC, and ^1^H–^13^C gHMBC were recorded using standard pulse sequences. The probe temperature (±1 K) was controlled by a standard unit calibrated with methanol as a reference. All NMR data processing was carried out using MestReNova version 10.0.2.

### Starting Materials

IrCl_3_·*x*H_2_O was purchased from Johnson Matthey and used as received. The starting dimer ([Ir(μ-Cl)(dfppy)_2_]_2_) (dfppy = 2-(2,4-difluorophenyl) pyridinate) was prepared according to the reported procedure.^2^ The reagents 2-(2,4-difluorophenyl)pyridine), iodomethane, and benzyl bromide were purchased from Sigma-Aldrich, 2-(2-pyridyl)benzimidazole and 4-iodobenzyl bromide were purchased from Acros Organics-Fisher Scientific, and 2-(bromomethyl)naphthalene was purchased from Alfa Aesar. All of them were used without further purification. Deuterated solvents (DMSO-*d*_6_, CDCl_3_, CD_3_CN) were obtained from Eurisotop. Conventional solvents such as diethyl ether (Fisher Scientific), acetone (Fisher Scientific), and 2-ethoxyethanol (Across Organics) were degassed and in some cases distilled prior to use. Acetonitrile used in the photocatalytic experiments were acquired from a Fisher Scientific (HPLC quality). Tetrabutylammonium hexafluorophosphate ([^*n*^Bu_4_N][PF_6_]) was purchased from Acros. The synthetic procedures of the ligands were previously described in the literature: L2,^3^ L3,^4^ L4,^3^ and L5.^5^

### X-ray Crystallography

A summary of crystal data collection and refinement parameters for ***rac*-[Ir1]Cl**, ***rac*-[Ir3]PF_6_**, ***rac*-[Ir4]PF_6_**, and ***rac*-[Ir5]PF_6_** are given in Table S1. Single crystals of compounds were coated in high-vacuum grease, mounted on a glass fiber, and transferred to a Bruker SMART APEX CCD-based diffractometer equipped with a graphite monochromated Cu–Kα radiation source (λ = 1.54178 Å) for ***rac*-[Ir1]Cl**, ***rac*-[Ir3]PF_6_**, and ***rac*-[Ir4]PF_6_** and MoKα (λ = 0.71073 Å) for ***rac*-[Ir5]PF_6_**. The highly redundant data sets were integrated using SAINT^6^ and corrected for Lorentz and polarization effects. The absorption correction was based on the function fitting to the empirical transmission surface as sampled by multiple equivalent measurements with the program SADABS.^7^

The software package WINGX^8,9^ was used for space group determination, structure solution, and refinement by full-matrix least-squares methods based on *F*^2^. A successful solution by direct methods provided most nonhydrogen atoms from the E-map. The remaining nonhydrogen atoms were located in an alternating series of least-squares cycles and difference Fourier maps. All nonhydrogen atoms were refined with anisotropic displacement coefficients. Hydrogen atoms were placed using a “riding model” and included in the refinement at calculated positions. CCDC reference numbers for ***rac*-[Ir1]Cl**, ***rac*-[Ir3]PF_6_**, ***rac*-[Ir4]PF_6_**, and ***rac*-[Ir5]PF_6_** are 2096987, 2096990, 2096988, and 2096989.

### Measurements of UV–vis Absorption and Photoluminescence Spectra

UV–vis absorption spectra were recorded in the 200–1100 nm spectral range by a Shimadzu UV-2450 spectrophotometer, using 10 mm quartz cells, while excitation and emission spectra were recorded on a FLS980 spectrofluorometer (from Edinburgh Instruments) equipped with triple grating turret monochromators and a Red PMT Sphere detector. The F980 spectrometer operating software was used to collect and process fluorescence data. Samples of 1 × 10^–5^ M solutions in CH_3_CN were prepared and deoxygenated in a Schlenk using freeze–pump–thaw technique. Then, the solutions were kept under inert atmosphere in quartz cuvettes equipped with Teflon septum screw caps for all the luminescence measurements. All optical measurements were made at room temperature.

The luminescence emission spectra were recorded by exciting at 405 nm with a xenon arc lamp, and the maximum emission wavelength was measured from 420 to 800 nm. The photoluminescence quantum yields (PLQY or Φ) were calculated by detecting all sample emission through the use of an integrating sphere. For the determination of the luminescence lifetime of compounds **[Ir1]Cl**–**[Ir5]Cl**, the fluorescence decay was measured on a FLS980 spectrofluorometer equipped with a TSCPC laser and a REDPMT detector. The F980 spectrometer operating software was used to collect and process luminescence lifetime data. The instrumental parameters used were as follows: λ_ex_ = 405 nm, Δλ_ex_ = 0.2 nm, λ_em_ = 648 nm, Δλ_em_ = 4 nm, 2000 channels, integration time = 1 μs, iris setting = 100.

### Electrochemical Measurements

Electrochemical measurements were performed using a portable potentiostat/galvanostat PalmSens3 (PalmSens) equipment controlled by the software PSTrace4 Version 4.4.2. All experiments were carried out using a three-electrode cell with a glassy carbon disc (diameter = 3 mm) as the working electrode, a platinum wire as the auxiliary electrode, and a Ag/AgCl (MF-2052 BASi) reference electrode separated from the bulk solution by a Vycor frit. Oxygen was removed from the solution by bubbling argon for 10 min and keeping the current of argon along the whole experiment. The measurements were recorded for acetonitrile solutions of the complexes (5 × 10^–4^ M) in the presence of [*^n^*Bu_4_N][PF_6_] (0.1 M) as the supporting electrolyte by CV at a scan rate of 100 mV·s^–1^ in a clockwise direction. Ferrocene was added at the end of all the experiments as the internal reference. The potential experimentally determined for the redox couple Fc^+^/Fc was *E*_1/2_° = 0.455 ± 0.002 V vs Ag/AgCl. Therefore, the experimental redox potentials were calculated from the corresponding voltammograms as

- *E*° (vs AgCl/Ag) = (*E*_ap_ + *E*_cp_)/2, for reversible peaks where *E*_ap_ and *E*_cp_ stand for anodic and cathodic peak potentials, respectively. However, for irreversible peaks, the potentials were calculated as either the *E*_ap_ maximum or *E*_cp_ minimum.
- *E*° (vs Fc^+^/Fc) = *E*° (vs AgCl/Ag) – 0.443, for potential values reported in reference to the (Fc^+^/Fc) redox couple.

## Supporting Information Available

General Information, synthesis and characterization of the Ir(III) complexes, X-ray diffraction, photostability, theoretical calculations and electrochemical measurements of the complexes, procedure for the photocatalytic oxidation of indolines and tetrahydroquinolines, ^1^H spectra and characterization of the crudes and isolated products, detection of H_2_O_2_ in photocatalytic experiments, Stern–Volmer experiments, pathway B for the reaction mechanism and photophysical properties (PDF)

## Accession Codes

CCDC 2096987–2096990 contain the supplementary crystallographic data for this paper. These data can be obtained free of charge via www.ccdc.cam.ac.uk/data_request/cif, or by emailing data_request@ccdc.cam.ac.uk, or by contacting The Cambridge Crystallographic Data Centre, 12 Union Road, Cambridge CB2 1EZ, UK; fax: +44 1223 336033.
