# Supplementary material for: Photocatalytic Aerobic Dehydrogenation of N-Heterocycles with Ir(III) Photosensitizers Bearing the 2(2′-Pyridyl)benzimidazole Scaffold
Source: Inorg Chem. 2022 Apr 8;61(16):6193–208. doi: 10.1021/acs.inorgchem.2c00358 (PMC9044454; doi:10.1021/acs.inorgchem.2c00358)
Supplement: Supplementary file 1 — ic2c00358_si_001.pdf [file ic2c00358_si_001.pdf]

Supporting Information

Photocatalytic Aerobic Dehydrogenation of N-Heterocycles with Ir(III) photosensitizers bearing the 2(2'-pyridyl)benzimidazole scaffold

Igor Echevarría,*[a] Mónica Vaquero,[a] Blanca R. Manzano,[b] Félix A. Jalón,[b] Roberto Quesada*[a] and Gustavo Espino*[a]

iepoza@ubu.es; mvaquero@ubu.es; blanca.manzano@uclm.es; felix.jalon@uclm.es; rquesada@ubu.e; gespino@ubu.es

[a]*Universidad de Burgos, Departamento de Química, Facultad de Ciencias, Plaza Misael Bañuelos s/n, 09001, Burgos, Spain.*

[b]*Universidad de Castilla-La Mancha, Departamento de Química Inorgánica, Orgánica y Bioquímica. Facultad de Químicas, Avda. Camilo J. Cela 10, 13071 Ciudad Real, Spain.*

Table of Contents

1.- General Information S-2
2.- Synthesis and characterization of the Ir(III)-complexes. S-4
3.- X-Ray diffraction: Crystallographic parameters. S-13
4.- Photostability of the Iridium complexes in CD_3CN. S-14
5.- Theoretical Calculations. S-18
6.- Electrochemical measurements. S-24
7.- Procedure for the photocatalytic oxidation of indolines and tetrahydroquinolines. S-25
8.- ^{1}H spectra and characterization of the crudes and isolated products. S-28
9.- Detection of H_2O_2 in photocatalytic experiments. S-39
10.- Stern-Volmer Experiments. S-41
11. Pathway B for the reaction mechanism. S-42
12. Photophysical properties. S-43
13. References. S-44

1.- General Information and procedures

All synthetic manipulations were carried out under an atmosphere of dry, oxygen-free nitrogen using standard Schlenk techniques. The solvents were dried and distilled under nitrogen atmosphere before use. Elemental analyses were performed with a Thermo Fisher Scientific EA Flash 2000 Elemental Microanalyzer. IR spectra were recorded on a Jasco FT/IR-4200 spectrophotometer (4000–400 cm^{-1} range) with Single Reflection ATR Measuring Attachment. UV-Vis absorption was measured in an Evolution 300 UV-Vis double beam spectrophotometer (Thermo Scientific). Fluorescence steady-state and lifetime measurements were performed in a FLS980 (Edinburg Instruments) Fluorimeter with Xenon Arc Lamp 450W and TCSPC laser, respectively. Quantum Yield was determined by using in a FLS980 (Edinburg Instruments) with Xenon Arc Lamp 450W and Red PMT Sphere as detector. HR-ESI(+) Mass spectra (position of the peaks in Da) were recorded with an Agilent LC-MS system (1260 Infinity LC / 6545 Q-TOF MS spectrometer) using DCM/DMSO (4:1) as the sample solvent and (0.1%) aqueous HCOOH/MeOH as the mobile phase. The experimental *m/z* values are expressed in Da compared with the *m/z* values for monoisotopic fragments. NMR samples were prepared by dissolving the suitable amount of compound in 0.5 mL of the respective deuterated solvent and the spectra were recorded at 298 K on a Varian Unity Inova-400 (399.94 MHz for ^{1}H; 376.29 MHz for ^{19}F; 100.6 MHz for ^{13}C). Typically, ^{1}H NMR spectra were acquired with 32 scans into 32 k data points over a spectral width of 16 ppm. ^{1}H and $^{13}C\{^{1}H\}$ chemical shifts were internally referenced to TMS via the residual ^{1}H and ^{13}C signals of DMSO-d_6 (δ = 2.50 ppm and δ = 39.52 ppm), CD_3CN (δ = 1.94 ppm and δ = 118.69 (-CN) and 1.39 (-CD_3) ppm) and $CDCl_3$ (δ = 7.26 ppm and δ = 77.16 ppm), according to the values reported by Fulmer et al.[1] Chemical shift values (δ) are reported in ppm and coupling constants (*J*) in Hertz. The splitting of proton resonances in the reported ^{1}H NMR data is defined as s = singlet, d = doublet, t = triplet, q = quartet, m = multiplet, bs = broad singlet. 2D NMR spectra such as ^{1}H-^{1}H gCOSY, ^{1}H-^{1}H NOESY, ^{1}H-^{13}C gHSQC and ^{1}H-^{13}C gHMBC were recorded using standard pulse sequences. The probe temperature ($\pm$1 K) was controlled by a standard unit calibrated with methanol as a reference. All NMR data processing was carried out using MestReNova version 10.0.2.

Starting materials. $IrCl_3 \cdot xH_2O$ was purchased from Johnson Matthey and used as received. The starting dimer ([Ir(μ-Cl)(dfppy)$_2$]$_2$) (dfppy = 2-(2,4-difluorophenyl) pyridinate) was prepared according to the reported procedure.[2] The reagents 2-(2,4-Difluorophenyl)pyridine), iodomethane and benzyl bromide were purchased from Sigma-Aldrich; 2-(2-pyridyl)benzimidazole and 4-iodobenzyl bromide were purchased from Acros Organics-Fisher Scientific, and 2-(bromomethyl)naphthalene was purchased from Alfa Aesar. All of them were used without further purification. Deuterated solvents (DMSO-d_6, $CDCl_3$, CD_3CN) were obtained from Eurisotop. Conventional solvents such as diethyl ether (Fisher Scientific), acetone (Fisher Scientific) and 2-ethoxyethanol (Across Organics) were degassed and in some cases distilled prior to use. Acetonitrile used in the photocatalytic experiments were acquired from a Fisher Scientific (HPLC quality). Tetrabutylammonium hexafluorophosphate ([$^{n}Bu_4N$][PF_6]) was purchased from Acros. Synthetic procedure of the ligands were previously described in the literature: L2,[3] L3,[4] L4[3] and L5.[5]

X-ray Crystallography. A summary of crystal data collection and refinement parameters for **rac-[Ir1]Cl, rac-[Ir3]PF$_6$**, **rac-[Ir4]PF$_6$** and **rac-[Ir5]PF$_6$** are given in Table SI-1. Single crystals of compounds were coated in high-vacuum grease, mounted on a glass fiber, and transferred to a Bruker SMART APEX CCD-based diffractometer equipped with a graphite monochromated Cu-Kα radiation source (λ = 1.54178 Å) for **rac-[Ir1]Cl**, **rac-[Ir3]PF$_6$** and **rac-[Ir4]PF$_6$** and MoKα (λ = 0.71073 Å) for **rac-[Ir5]PF$_6$**. The highly redundant datasets were integrated using SAINT[6] and corrected for Lorentz and polarization effects. The absorption correction was based on the function fitting to the empirical transmission surface as sampled by multiple equivalent measurements with the program SADABS.[7]

The software package WINGX[8,9] was used for space group determination, structure solution, and refinement by full-matrix least-squares methods based on F^2. A successful solution by direct methods

provided most non-hydrogen atoms from the E-map. The remaining non-hydrogen atoms were located in an alternating series of least-squares cycles and difference Fourier maps. All non-hydrogen atoms were refined with anisotropic displacement coefficients. Hydrogen atoms were placed using a “riding model” and included in the refinement at calculated positions. CCDC reference numbers for **rac-[Ir1]Cl, rac-[Ir3]PF_6**, **rac-[Ir4]PF_6** and **rac-[Ir5]PF_6** are **2096987**, **2096990**, **2096988** and **2096989**.

Measurements of UV-Vis Absorption and photoluminescence Spectra. UV-Vis absorption spectra were recorded in the 200-1100 nm spectral range by a Shimadzu UV-2450 spectrophotometer, using 10 mm quartz cells, while excitation and emission spectra were recorded on a FLS980 spectrofluorometer (from Edinburgh Instruments) equipped with triple grating turret monochromators and a Red PMT Sphere detector. The F980 spectrometer operating software was used to collect and process fluorescence data. Samples of $1{\cdot}10^{-5}$ M solutions in CH_3CN were prepared and deoxygenated in a Schlenk using Freeze-Pump-Thaw technique. Then, the solutions were kept under inert atmosphere in quartz cuvettes equipped with Teflon septum screw caps for all the luminescence measurements. All optical measurements were made at room temperature.

The luminescence emission spectra were recorded by exciting at 405 nm with a Xenon Arc lamp and the maximum emission wavelength was measured from 420 to 800 nm. The photoluminescence quantum yields (PLQY or Φ) were calculated by detecting all sample emission through the use of an integrating sphere. For the determination of the luminescence lifetime of compounds **[Ir1]Cl-[Ir5]Cl**, the fluorescence decay was measured on a FLS980 spectrofluorometer equipped with a TSCPC laser and a REDPMT detector. The F980 spectrometer operating software was used to collect and process luminescence lifetime data. The instrumental parameters used were as follows: λ_{ex} = 405 nm, $\Delta\lambda_{ex}$ = 0.2 nm, λ_{em} = 648 nm, $\Delta\lambda_{em}$ = 4 nm, 2000 channels, integration time = 1 µs, iris setting = 100.

Electrochemical measurements. Electrochemical measurements were performed using a portable potentiostat/galvanostat PalmSens3 (PalmSens) equipment controlled by the software PSTrace4 Version 4.4.2. All experiments were carried out using a three-electrode cell with a glassy carbon-disc (diameter = 3 mm) as the working electrode, a platinum-wire as the auxiliary electrode, and a Ag/AgCl (MF-2052 BASi) reference electrode separated from the bulk solution by a Vycor™ frit. Oxygen was removed from the solution by bubbling argon for 10 minutes and keeping the current of argon along the whole experiment. The measurements were recorded for acetonitrile solutions of the complexes (5×10^{-4} M) in the presence of [$^nBu_4N$][PF_6] (0.1 M) as the supporting electrolyte by cyclic voltammetry (CV) at a scan rate of 100 $mV{\cdot}s^{-1}$ in a clockwise direction. Ferrocene was added at the end of all the experiments as the internal reference. The potential experimentally determined for the redox couple Fc^+/Fc was $E°_{1/2}$ = 0.455±0.002 V vs. Ag/AgCl. Therefore, the experimental redox potentials were calculated from the corresponding voltammograms as:

E° (vs AgCl/Ag) = (E_{ap} + E_{cp})/2, for reversible peaks where E_{ap} and E_{cp} stand for anodic and cathodic peak potentials, respectively. However, for irreversible peaks, the potentials were calculated as either the E_{ap} maximum or E_{cp} minimum.

E° (vs Fc^+/Fc) = E° (vs AgCl/Ag) – 0.443, for potential values reported in reference to the (Fc^+/Fc) redox couple.

2.- Synthesis and characterization of the Ir(III)-complexes

Synthesis of [Ir(dfppy)$_2$(L1)]Cl: [Ir1]Cl

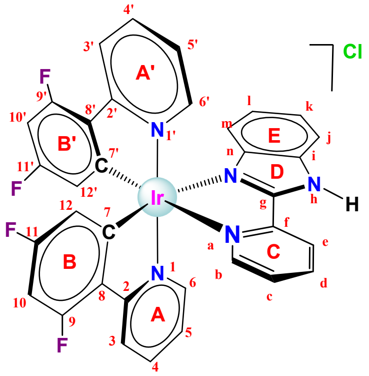

In a 100 mL Schlenk flask, previously purged with nitrogen, the ancillary ligand **L1** (0.0323 g, 0.165 mmol) was added to a solution of [Ir(μ-Cl)(dfppy)$_2$]$_2$ (0.1003 g, 0.082 mmol) in a mixture of dichloromethane (8 mL) / methanol (10 mL), and the mixture was stirred at 60 °C for 24 hours under a N_2 atmosphere. The resulting solution was concentrated to half the volume under vacuum and diethyl ether (15 mL) was added to precipitate a crude solid that was isolated by filtration and washed with diethyl ether (2×5 mL). The product was dried under vacuum to produce a yellow powder. Yield: 0.0940 g (0.117 mmol, 71%). **M_r ($C_{34}H_{21}ClF_4IrN_5$)** = 803.23 g/mol. **Anal. Calcd for $C_{34}H_{21}ClF_4IrN_5(CH_2Cl_2)_{0.15}$:** C 50.27; H 2.63; N 8.58; **Found:** C 50.55; H 2.59; N 8.24. **^{1}H NMR (400 MHz, DMSO-d$_6$, 25 °C)** δ 15.91 (bs, 1H, NH), 9.06 (d, J = 7.4 Hz, 1H, H^e), 8.38 (t, J = 7.8 Hz, 1H, H^d), 8.25 (dd, J = 21.6, 8.3 Hz, 2H, H^3, $H^{3'}$), 8.07 – 7.85 (m, 3H, H^4, $H^{4'}$, H^b), 7.83 – 7.64 (m, 4H, H^j, H^6, $H^{6'}$, H^c), 7.39 (t, J = 7.7 Hz, 1H, H^k), 7.22 (q, J = 5.9 Hz, 2H, H^5, $H^{5'}$), 7.13 (t, J = 7.7 Hz, 1H, H^l), 7.08 – 6.87 (m, 2H, H^{10}, $H^{10'}$), 6.21 (d, J = 8.3 Hz, 1H, H^m), 5.76 (d, J = 7.1 Hz, 1H, H^{12}), 5.67 (d, J = 7.4 Hz, 1H, $H^{12'}$) ppm. **^{19}F NMR (376 MHz, DMSO-d$_6$, 25 °C)** δ -106.68 (q, J = 9.7 Hz, 1F, F^{11}), -107.11 (q, J = 9.2 Hz, 1F, $F^{11'}$), -108.83 (t, J = 11.5 Hz, 1F, F^9), -109.31 (t, J = 11.8 Hz, 1F, $F^{9'}$) ppm. **^{13}C{^{1}H} NMR (101 MHz, DMSO-d$_6$, 25 °C)** δ 163.38, 162.69, 161.53, 160.84, 158.94, 155.06, 153.29, 151.27, 150.54, 149.93, 149.84, 149.73, 149.64, 146.94, 140.20, 139.61, 139.38, 134.51, 128.80, 128.16, 127.78, 125.36, 124.71, 124.30, 123.09, 122.90, 122.60, 115.56, 114.04, 113.97, 113.47, 112.90, 98.71, 98.55 ppm. **FT-IR (KBr, cm^{-1}) selected bands:** 3333 (w, $\nu_{N\text{-}H}$), 3017 (w, $\nu_{C=CH}$), 1602-1571 (m, $\nu_{C=C + C\text{-}N}$), 1429 (w, $\nu_{C=N}$), 1165 (m, $\nu_{C\text{-}C}$), 1042 (m, $\delta_{C\text{-}Hip}$), 750-739 (vs, $\delta_{C\text{-}Hoop}$). **HR ESI+ MS (DCM/DMSO, 4:1):** m/z_{exp} = 768.1368 (m/z_{calcd} [M^+] = m/z_{calcd} [$C_{34}H_{21}F_4IrN_5$]$^+$ = 768.1362)); 573.0560 (m/z_{calcd} [M^+-L1] = m/z_{calcd} [$C_{22}H_{12}F_4IrN_2$]$^+$ = 573.0566). **Solubility:** soluble in dimethyl sulfoxide, dichloromethane, methanol, acetone, acetonitrile, dimethylformamide, tetrahydrofuran.

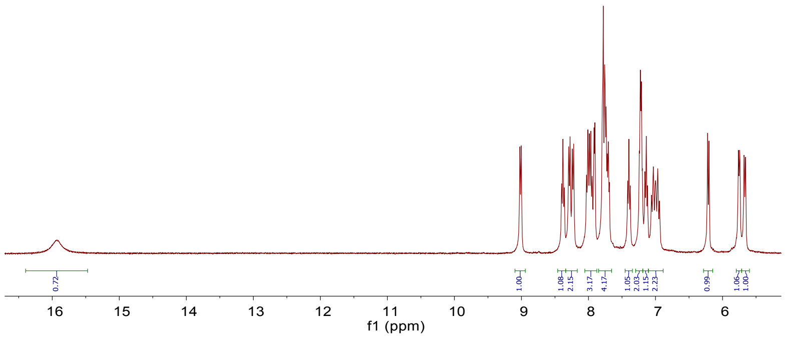

Figure S1. ^{1}H NMR spectrum of **[Ir1]Cl**. (400 MHz) recorded in DMSO-d_6 at 25 °C.

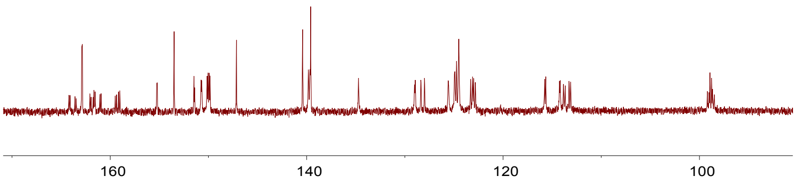

Figure S2. $^{13}C\{^{1}H\}$ NMR (101 MHz, DMSO-d_6, 25 °C) spectrum of **[Ir1]Cl**.

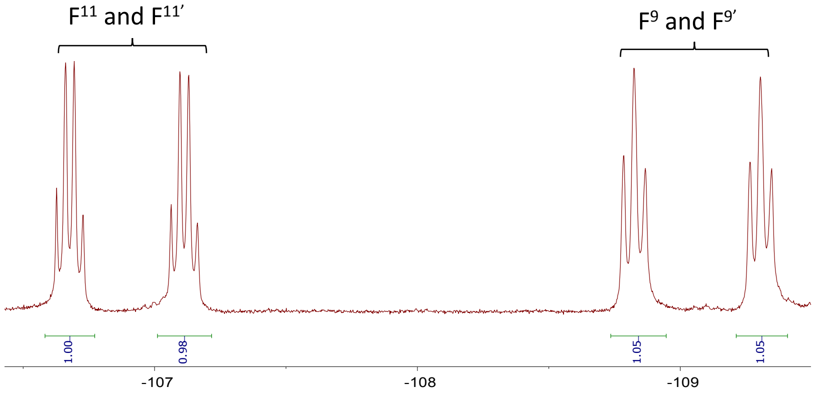

Figure S3. ^{19}F NMR (376 MHz, DMSO-d_6, 25 °C) spectrum of **[Ir1]Cl**.

Synthesis of $[Ir(dfppy)_2(L2)]Cl$: [Ir2]Cl

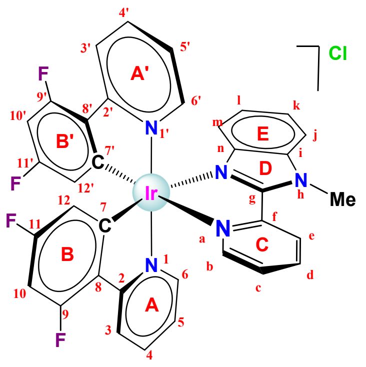

In a 100 mL Schlenk flask, previously purged with nitrogen, the ancillary ligand **L2** (0.0387 g, 0.185 mmol) was added to a solution of $[Ir(\mu\text{-}Cl)(dfppy)_2]_2$ (0.1001 g, 0.082 mmol) in a mixture of dichloromethane (8 mL) / methanol (10 mL), and the mixture was stirred at 60 ºC for 24 hours under a N_2 atmosphere. The resulting solution was concentrated to half the volume under vacuum and diethyl ether (15 mL) was added to precipitate a crude solid that was isolated by filtration and washed with diethyl ether (2×5 mL). The product was dried under vacuum to produce a yellow powder. Yield: 0.0896 g (0.110 mmol, 67%). **M_r ($C_{35}H_{23}ClF_4IrN_5$)** = 817.25 g/mol. **Anal. Calcd for $C_{35}H_{23}ClF_4IrN_5(CH_2Cl_2)_{0.88}$:** C 48.31; H 2.80; N 7.85; **Found:** C 48.35; H 2.85; N 8.19. **^{1}H NMR (400 MHz, DMSO-d_6, 25 °C)** δ 8.85 (d, J = 8.4 Hz, 1H, H^e), 8.36 (t, J = 8.0 Hz, 1H, H^d), 8.30 (d, J = 8.7 Hz, 1H, H^3), 8.23 (d, J = 8.6 Hz, 1H, $H^{3'}$), 8.05 – 7.94 (m, 4H, H^4, $H^{4'}$, H^j, H^b), 7.80 (d, J = 5.9 Hz, 1H, H^6), 7.77 – 7.68 (m, 2H, $H^{6'}$, H^c), 7.48 (t, J = 7.8 Hz, 1H, H^k), 7.27 – 7.14 (m, 3H, $H^{5'}$, H^5, H^l), 7.08 – 6.93 (m, 2H, H^{10}, $H^{10'}$), 6.24 (d, J = 8.3 Hz, 1H, H^m), 5.72 (d, J = 8.4 Hz, 1H, H^{12}), 5.63 (d, J = 8.4 Hz, 1H, $H^{12'}$), 4.48 (s, 3H, $H^{N\text{-}Me}$) ppm. **^{13}C{^{1}H} NMR (101 MHz, DMSO-d_6, 25 °C)** δ 162.74, 159.36, 159.01, 155.65, 155.08, 152.83, 151.90, 151.42, 151.32, 150.19, 149.35, 148.02, 146.67, 145.18, 139.82, 138.32, 136.51, 134.23, 133.13, 130.81, 130.11, 127.82, 126.48, 125.10, 124.37, 124.26, 123.34, 120.46, 120.10, 113.76, 112.93, 98.78, 33.52 ppm. **^{19}F NMR (376 MHz, DMSO-d_6, 25 °C)** δ -106.57 (q, J = 9.6 Hz, 1F), -107.01 (q, J = 8.9 Hz, 1F), -108.75 (t, J = 11.2 Hz, 1F), -109.21 (t, J = 11.2 Hz, 1F) ppm. **FT-IR (KBr, cm^{-1}) selected bands:** 3064 (w, $\nu_{C=CH}$), 1601-1571 (m, $\nu_{C=C + C\text{-}N}$), 1428 (w, $\nu_{C=N}$), 1162 (m, $\nu_{C\text{-}C}$), 1042 (m, $\delta_{C\text{-}Hip}$), 746 (vs, $\delta_{C\text{-}Hoop}$). **HR ESI+ MS (DCM/DMSO, 4:1):** m/z_{exp} = 782.1520 (m/z_{calcd} [M^+] = m/z_{calcd} $[C_{35}H_{23}F_4IrN_5]^+$ = 782.1519); 573.0558 (m/z_{calcd} [M^+-L2] = m/z_{calcd} $[C_{22}H_{12}F_4IrN_2]^+$ = 573.0566). **Solubility:** soluble in dimethyl sulfoxide, dichloromethane, methanol, acetone, acetonitrile, dimethylformamide, tetrahydrofuran.

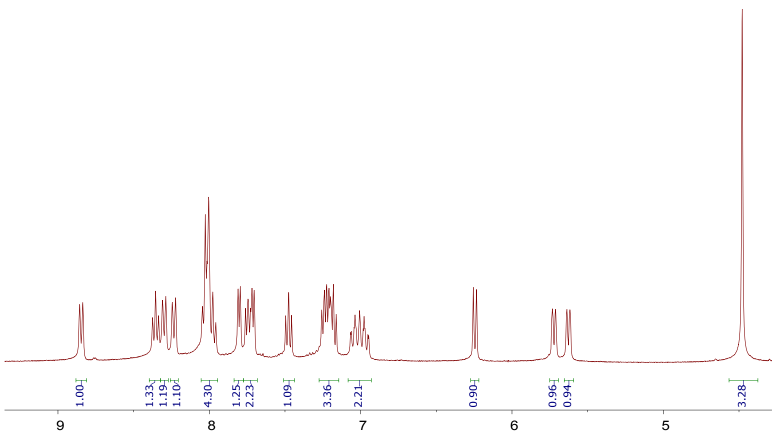

Figure S4. ^{1}H NMR (400 MHz, DMSO-d$_6$, 25 °C) spectrum of **[Ir2]Cl**.

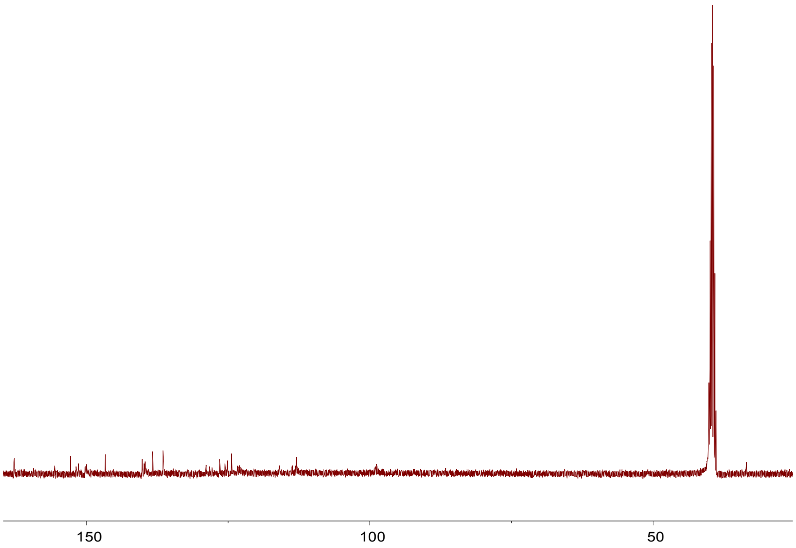

Figure S5. ^{13}C{^{1}H} NMR (101 MHz, DMSO-d$_6$, 25 °C) spectrum of **[Ir2]Cl**.

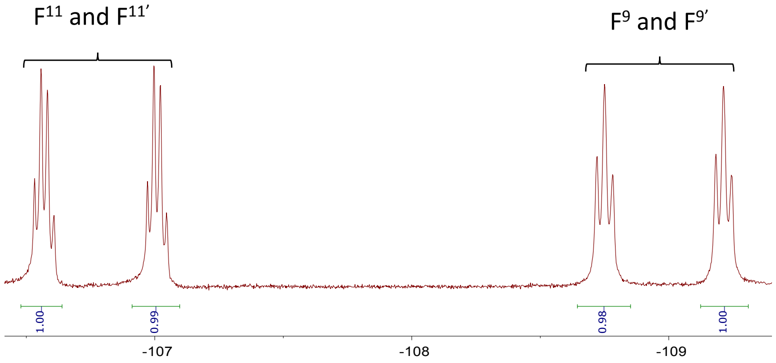

Figure S6. ^{19}F NMR (376 MHz, DMSO-d$_6$, 25 °C) spectrum of **[Ir2]Cl**.

Synthesis of [Ir(dfppy)$_2$(L3)]Cl: [Ir3]Cl

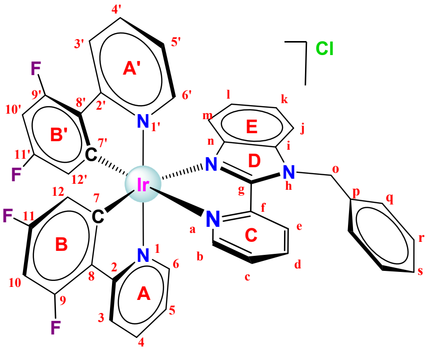

In a 100 mL Schlenk flask, previously purged with nitrogen, the ancillary ligand **L3** (0.0530 g, 0.186 mmol) was added to a solution of [Ir(μ-Cl)(dfppy)$_2$]$_2$ (0.1003 g, 0.082 mmol) in a mixture of dichloromethane (8 mL) / methanol (10 mL), and the mixture was stirred at 60 ºC for 24 hours under a N_2 atmosphere. The resulting solution was concentrated to half the volume under vacuum and diethyl ether (15 mL) was added

to precipitate a crude solid that was isolated by filtration and washed with diethyl ether (2×5 mL). The product was dried under vacuum to produce a yellow powder. Yield: 0.1216 g (0.136 mmol, 83%). **M_r ($C_{41}H_{27}ClF_4IrN_5$)** = 893.35 g/mol. **Anal. Calcd for $C_{41}H_{27}ClF_4IrN_5{\cdot}(CH_2Cl_2)_{1.2}$:** C 50.93; H 2.98; N 7.04; **Found:** C 50.97; H 3.24; N 7.35. **^{1}H NMR (400 MHz, DMSO-d_6, 25 °C)** δ 8.54 (d, J = 8.4 Hz, 1H, H^e), 8.31 (d, J = 8.6 Hz, 1H, H^3), 8.27 – 8.17 (m, 2H, $H^{3'}$, H^d), 8.01 (td, J = 14.6, 14.1, 6.8 Hz, 4H, H^4, $H^{4'}$, H^j, H^b), 7.91 (d, J = 5.9 Hz, 1H, H^6), 7.71 – 7.62 (m, 2H, $H^{6'}$, H^c), 7.53 – 7.44 (m, 1H, H^k), 7.29 (ddt, J = 28.6, 12.8, 6.9 Hz, 6H, H^r, H^r, H^s, H^5, $H^{5'}$, H^l), 7.13 – 6.94 (m, 4H, H^q, H^q, H^{10}, $H^{10'}$), 6.41 – 6.23 (m, 3H, H^o, H^o, H^m), 5.77 (dd, J = 8.4, 2.1 Hz, 1H, H^{12}), 5.64 (dd, J = 8.4, 2.1 Hz, 1H, $H^{12'}$) ppm. **^{19}F NMR (376 MHz, DMSO-d_6, 25 °C)** δ -106.51 (q, J = 9.6 Hz, 1F), -106.89 (q, J = 9.2, 8.6 Hz, 1F), -108.63 (t, J = 11.9 Hz, 1F), -109.13 (t, J = 11.6 Hz, 1F) ppm. **^{13}C{^{1}H} NMR (101 MHz, DMSO-d_6, 25 °C)** δ 162.77, 155.17, 152.52, 152.48, 151.71, 150.20, 149.66, 145.70, 140.30, 139.98, 139.89, 138.44, 136.69, 135.17, 135.13, 129.25, 129.16, 129.12, 128.02, 126.39, 125.87, 124.54, 124.36, 123.19, 116.36, 113.87, 113.65, 113.25, 113.02, 99.07, 98.90, 48.36 ppm. **FT-IR (KBr, cm^{-1}) selected bands:** 3063 (w, $\nu_{C=CH}$), 1602-1569 (m, $\nu_{C=C\,+\,C-N}$), 1429 (w, $\nu_{C=N}$), 1165 (m, ν_{C-C}), 1067-1024-1013 (m, δ_{C-Hip}), 741 (vs, δ_{C-Hoop}). **HR ESI+ MS (DCM/DMSO, 4:1):** m/z_{exp} = 858.1830 (m/z_{calcd} [M^+] = m/z_{calcd} [$C_{41}H_{27}F_4IrN_5$]$^+$ = 858.1831); 573.0554 (m/z_{calcd} [M^+-L^3] = m/z_{calcd} [$C_{22}H_{12}F_4IrN_2$]$^+$ = 573.0566). **Solubility:** soluble in dimethyl sulfoxide, dichloromethane, methanol, acetone, acetonitrile, dimethylformamide, tetrahydrofuran.

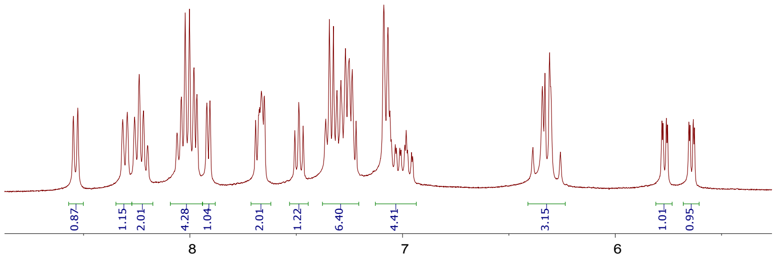

Figure S7. ^{1}H NMR (400 MHz, DMSO-d_6, 25 °C) spectrum of **[Ir3]Cl**.

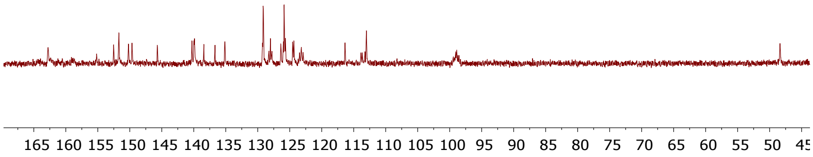

Figure S8. ^{13}C{^{1}H} NMR (101 MHz, DMSO-d_6, 25 °C) spectrum of **[Ir3]Cl**.

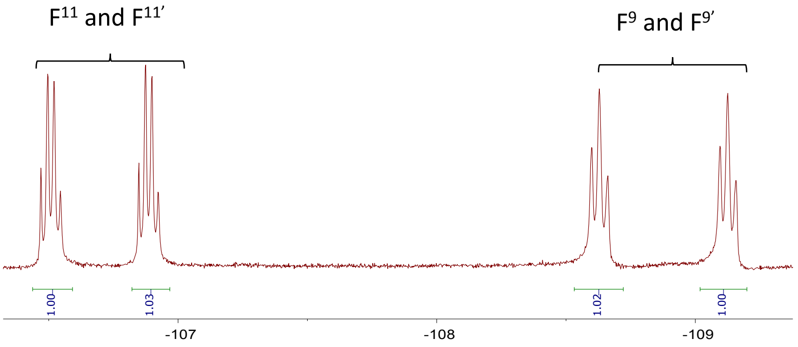

Figure S9. ^{19}F NMR (376 MHz, DMSO-d$_6$, 25 °C) spectrum of **[Ir3]Cl**.

Synthesis of [Ir(dfppy)$_2$(L4)]Cl: [Ir4]Cl

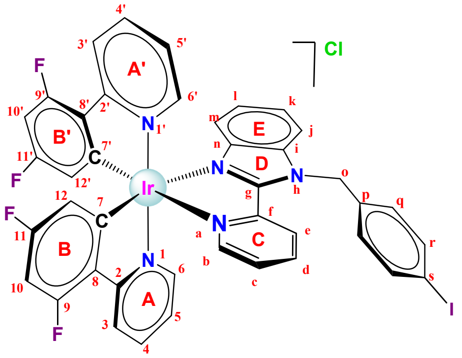

In a 100 mL Schlenk flask, previously purged with nitrogen, the ancillary ligand **L4** (0.0758 g, 0.184 mmol) was added to a solution of [Ir(µ-Cl)(dfppy)$_2$]$_2$ (0.1005 g, 0.083 mmol) in a mixture of dichloromethane (8 mL) / methanol (10 mL), and the mixture was stirred at 60 ºC for 24 hours under a N_2 atmosphere. The resulting solution was concentrated to half the volume under vacuum and diethyl ether (15 mL) was added to precipitate a crude solid tat was isolated by filtration and washed with diethyl ether (2×5 mL). The product was dried under vacuum to produce a yellow powder. Yield: 0.1308 g (0.128 mmol, 78%). **M_r ($C_{41}H_{26}ClF_4IIrN_5$)** = 1019.25 g/mol. **Anal. Calcd for $C_{41}H_{26}ClF_4IIrN_5$:** C 48.32; H 2.57; N 6.87; **Found:** C 48.34; H 2.60; N 6.54. **^{1}H NMR (400 MHz, DMSO-d$_6$, 25 ºC)** δ 8.48 (d, J = 8.2 Hz, 1H, H^e), 8.30 (d, J = 8.8 Hz, 1H, H^3), 8.27 – 8.19 (m, 2H, H$^{3'}$, H^d), 8.01 (dd, J = 16.4, 8.0 Hz, 4H, H^4, H$^{4'}$, H^j, H^b), 7.89 (d, J = 5.9 Hz, 1H, H^6), 7.74 – 7.65 (m, 4H, H$^{6'}$, H^r, H^r, H^c), 7.49 (t, J = 7.7 Hz, 1H, H^k), 7.25 (dt, J = 14.0, 7.3 Hz, 3H, H^5, H$^{5'}$, H^l), 7.10 – 7.02 (m, 1H, H^{10}), 7.02 – 6.95 (m, 1H, H$^{10'}$), 6.92 (d, J = 7.0 Hz, 2H, H^q, H^q), 6.36 – 6.19 (m, 3H, H^o, H^o, H^m), 5.76 (d, J = 8.4 Hz, 1H, H^{12}), 5.63 (d, J = 8.4 Hz, 1H, H$^{12'}$) ppm. **^{19}F NMR (376 MHz, DMSO-d$_6$, 25 ºC)** δ -106.50 (q, J = 9.9, 9.3 Hz, 1F), -106.90 (q, J = 9.6 Hz, 1F), -108.63 (t, J = 11.6 Hz, 1F), -109.13 (t, J = 11.6 Hz, 1F) ppm. **^{13}C{^{1}H} NMR (101 MHz, DMSO-d$_6$, 25 ºC)** δ 164.05, 163.64, 162.75, 161.63, 161.51, 161.10, 159.37, 159.11, 155.14, 153.39, 152.44, 151.72, 151.65, 145.59, 140.37, 139.99, 139.89, 138.46, 137.78, 136.60, 134.96, 129.28, 128.33, 128.19, 127.80, 126.46, 125.92, 125.72, 124.54, 124.40, 123.45, 123.25, 122.92, 116.34, 113.22, 112.96, 112.75, 99.07, 98.65, 94.29, 47.98 ppm. **FT-IR (KBr, cm^{-1}) selected bands:** 3011 (w, $\nu_{C=CH}$), 1600-1571 (m, $\nu_{C=C\ +\ C\text{-}N}$), 1441 (w, $\nu_{C=N}$), 1162 (m, $\nu_{C\text{-}C}$), 1061 (m, $\delta_{C\text{-}Hip}$), 755-745 (vs, $\delta_{C\text{-}Hoop}$). **HR ESI+ MS (DCM/DMSO, 4:1):** m/z$_{exp}$ = 984.0792 (m/z$_{calcd}$ [M$^+$] = m/z$_{calcd}$ [$C_{41}H_{26}F_4IIrN_5$]$^+$ = 984.0798); 573.0553 (m/z$_{calcd}$ [M$^+$-L^4] = m/z$_{calcd}$ [$C_{22}H_{12}F_4IrN_2$]$^+$ = 573.0566). **Solubility:** soluble in dimethyl sulfoxide, dichloromethane, methanol, acetone, acetonitrile, dimethylformamide, tetrahydrofuran.

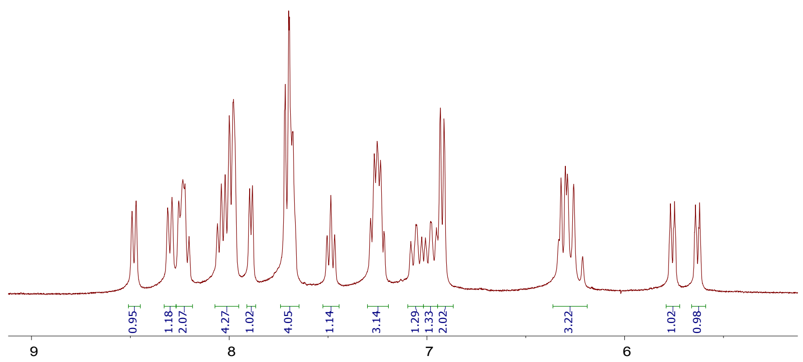

Figure S10. ^{1}H NMR (400 MHz, DMSO-d_6, 25 °C) spectrum of **[Ir4]Cl**.

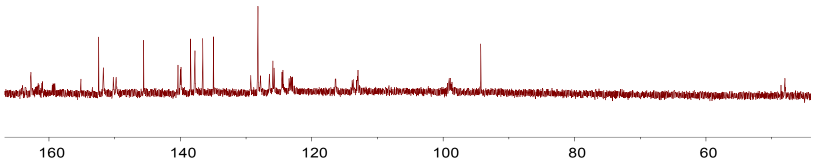

Figure S11. $^{13}C\{^{1}H\}$ NMR (101 MHz, DMSO-d_6, 25 °C) spectrum of **[Ir4]Cl**.

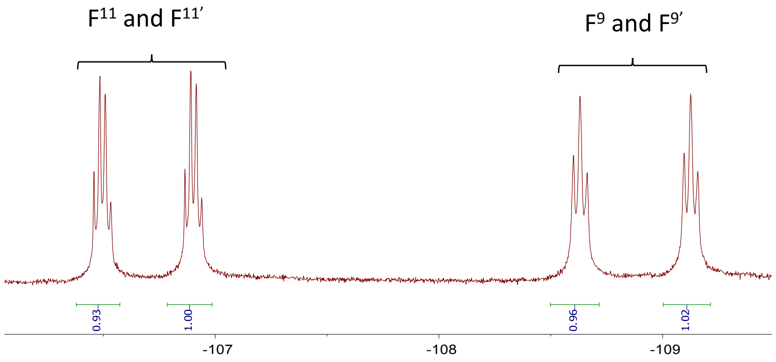

Figure S12. ^{19}F NMR (376 MHz, DMSO-d_6, 25 °C) spectrum of **[Ir4]Cl**.

Synthesis of [Ir(dfppy)$_2$(L5)]Cl: [Ir5]Cl

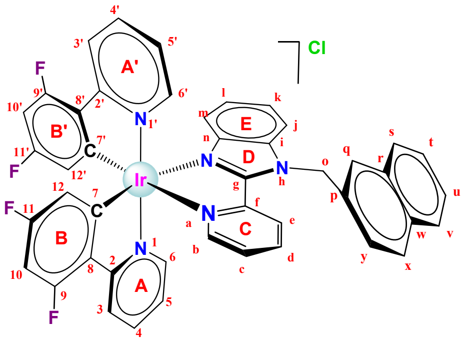

In a 100 mL Schlenk flask, previously purged with nitrogen, the ancillary ligand **L5** (0.0620 g, 0.185 mmol) was added to a solution of [Ir(μ-Cl)(dfppy)$_2$]$_2$ (0.1003 g, 0.082 mmol) in a mixture of dichloromethane (8 mL) / methanol (10 mL), and the mixture was stirred at 60 ºC for 24 hours under a N_2 atmosphere. The resulting solution was concentrated to half the volume under vacuum and diethyl ether (15 mL) was added to precipitate a crude solid that was isolated by filtration and washed with diethyl ether (2×5 mL). The product was dried under vacuum to produce a yellow-orange powder. Yield: 0.1206 g (0.128 mmol, 77%). **M$_r$ ($C_{45}H_{29}ClF_4IrN_5$)** = 943.41g/mol. **Anal. Calcd for $C_{45}H_{29}ClF_4IrN_5$·$(CH_2Cl_2)_{0.85}$:** C 54.22; H 3.05; N 6.90; **Found:** C 54.25; H 2.95; N 7.22. **^{1}H NMR (400 MHz, DMSO-d$_6$, 25 ºC)** δ 8.52 (d, J = 8.2 Hz, 1H, H^e), 8.29 (t, J = 9.1 Hz, 2H, H^3, $H^{3'}$), 8.18 (t, J = 8.0 Hz, 1H, H^d), 8.05 (t, J = 7.6 Hz, 3H, H^4, $H^{4'}$, H^j), 8.00 (d, J = 5.7 Hz, 1H), 7.96 (d, J = 9.0 Hz, 2H, H^b), 7.91 (dd, J = 6.0, 3.3 Hz, 1H), 7.78 – 7.73 (m, 1H), 7.71 (d, J = 5.7 Hz, 1H, $H^{6'}$), 7.68 – 7.61 (m, 1H, H^c), 7.58 – 7.46 (m, 4H, H^k, H^6), 7.35 (d, J = 8.6 Hz, 1H), 7.34 – 7.29 (m, 1H, H^5), 7.29 – 7.20 (m, 2H, $H^{5'}$, H^l), 7.07 (t, J = 10.9 Hz, 1H, H^{10}), 6.98 (t, J = 11.1 Hz, 1H, $H^{10'}$), 6.49 (q, J = 18.1 Hz, 2H, H^o, H^o), 6.36 (d, J = 8.4 Hz, 1H, H^m), 5.79 (d, J = 8.2 Hz, 1H, H^{12}), 5.64 (d, J = 8.3 Hz, 1H, $H^{12'}$) ppm. **^{19}F NMR (376 MHz, DMSO-d$_6$, 25 ºC)** δ -106.49 (q, J = 9.6 Hz, 1F), -106.92 (q, J = 9.6 Hz, 1F), -108.62 (t, J = 11.6 Hz, 1F), -109.11 (t, J = 11.2 Hz, 1F) ppm. **^{13}C{^{1}H} NMR (101 MHz, DMSO-d$_6$, 25 ºC)** δ 162.80, 161.12, 159.14, 153.87, 153.52, 152.53, 151.69, 150.28, 149.82, 145.66, 140.78, 140.29, 139.99, 139.85, 138.57, 137.32, 136.77, 136.23, 132.80, 132.78, 132.38, 131.62, 130.38, 129.22, 129.00, 127.70, 127.65, 126.71, 126.42, 125.96, 125.70, 124.56, 124.39, 124.18, 124.01, 122.95, 122.18, 116.41, 113.84, 113.05, 111.41, 98.91, 48.61 ppm. **FT-IR (KBr, cm^{-1}) selected bands:** 3016 (w, $\nu_{C=CH}$), 1601-1575 (m, $\nu_{C=C\ +\ C-N}$), 1427 (w, $\nu_{C=N}$), 1160 (m, ν_{C-C}), 1065 (m, δ_{C-Hip}), 756 (vs, δ_{C-Hoop}). **HR ESI+ MS (DCM/DMSO, 4:1):** m/zexp = 908.1986 (m/zcalcd [M$^+$] = m/z$_{calcd}$ [$C_{45}H_{29}F_4IrN_5$]$^+$ = 908.1988); 573.0553 (m/z$_{calcd}$ [M$^+$-L^5] = m/z$_{calcd}$ [$C_{22}H_{12}F_4IrN_2$]$^+$ = 573.0566). **Solubility:** soluble in dimethyl sulfoxide, dichloromethane, methanol, acetone, acetonitrile, dimethylformamide, tetrahydrofuran.

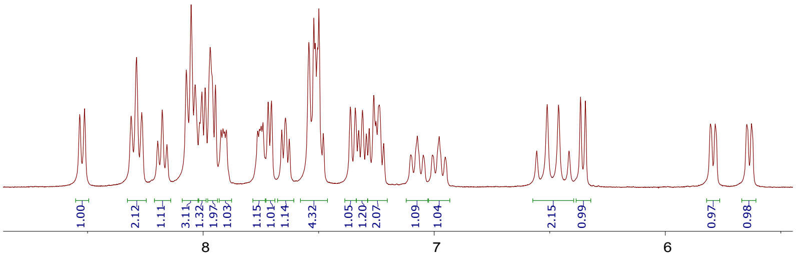

Figure S13. ^{1}H NMR (400 MHz, DMSO-d_6, 25 °C) spectrum of **[Ir5]Cl**.

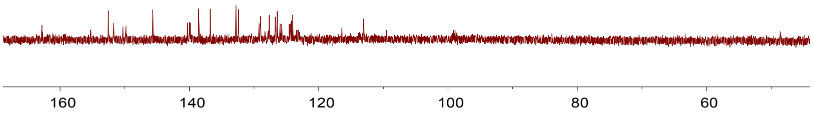

Figure S14. $^{13}C\{^{1}H\}$ NMR (101 MHz, DMSO-d_6, 25 °C) spectrum of **[Ir5]Cl**.

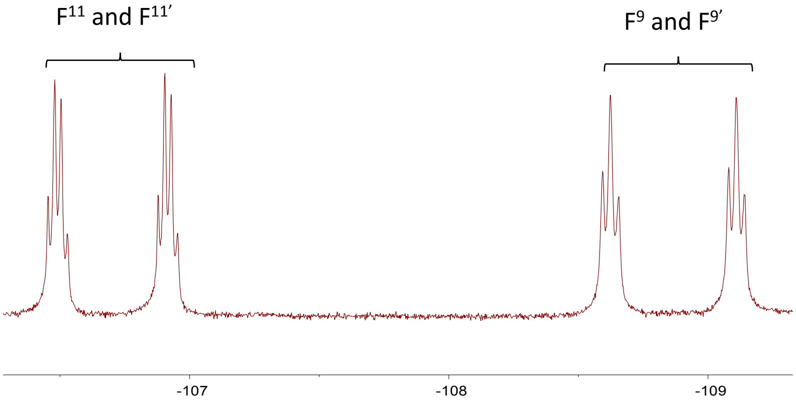

Figure S15. ^{19}F NMR (376 MHz, DMSO-d_6, 25 °C) spectrum of **[Ir5]Cl**.

3.- X-Ray diffraction: Crystallographic parameters

Table S1. Crystallographic Parameters for the crystal structures of **[Ir1]Cl·MeOH**, **[Ir3)]PF$_6$**, **[Ir4)]PF$_6$**, **[Ir5)]PF$_6$**.

Complex	**[Ir1]Cl·MeOH**	**[Ir3)]PF$_6$**	**[Ir4)]PF$_6$**	**[Ir5)]PF$_6$**
Empirical formula	$C_{35}H_{25}ClF_4IrN_5O$	$C_{41}H_{27}F_{10}IrN_5P$	$C_{41}H_{26}F_{10}IIrN_5P$	$C_{45}H_{29}F_{10}IrN_5P$
Formula weight	835.25	1002.84	1128.74	1052.90
Temperature/K	299.0	301.0	250.0	180.0
Crystal system	monoclinic	monoclinic	triclinic	monoclinic
Space group	$P2_1/c$	$P2_1/c$	P-1	$P2_1/n$
a/Å	12.4429(13)	10.3428(6)	9.2856(19)	8.7066(6)
b/Å	16.5636(17)	25.3654(15)	14.962(3)	50.347(3)
c/Å	15.6830(15)	14.7388(9)	15.201(3)	18.4690(12)
α/°	90	90	79.205(9)	90
β/°	105.145(4)	98.063(2)	74.024(9)	94.448(2)
γ/°	90	90	80.187(9)	90
Volume/Å^3	3120.0(5)	3828.5(4)	1978.6(7)	8071.5(9)
Z	4	4	2	8
ρ_{calc}g/cm^3	1.778	1.740	1.895	1.733
μ/mm^{-1}	9.611	7.923	13.800	3.435
F(000)	1632.0	1960.0	1084.0	4128.0
Crystal size/mm^3	0.1 × 0.08 × 0.05	0.7 × 0.05 × 0.05	0.1 × 0.08 × 0.05	0.5 × 0.1 × 0.1
Radiation	CuKα (λ = 1.54178)	CuKα (λ = 1.54178)	CuKα (λ = 1.54178)	MoKα (λ = 0.71073)
2Θ range for data collection/°	7.36 to 144.52	6.97 to 144.356	6.062 to 145.228	4.498 to 49.548
Index ranges	$-15 \le h \le 15, -19 \le k \le 20, -19 \le l \le 18$	$-12 \le h \le 12, -31 \le k \le 29, -18 \le l \le 16$	$-11 \le h \le 10, -18 \le k \le 18, -18 \le l \le 18$	$-9 \le h \le 10, -59 \le k \le 59, -21 \le l \le 21$
Reflections collected	44556	64555	55256	99981
Independent reflections	6152 [R_{int} = 0.0593, R_{sigma} = 0.0330]	7533 [R_{int} = 0.0521, R_{sigma} = 0.0263]	7806 [R_{int} = 0.0752, R_{sigma} = 0.0412]	13818 [R_{int} = 0.0599, R_{sigma} = 0.0338]
Data/restraints/parameters	6152/0/426	7533/0/523	7806/36/540	13818/15/1114
Goodness-of-fit on F^2	1.049	1.141	1.032	1.136
Final R indexes [I>=2σ (I)]	R_1 = 0.0434, wR_2 = 0.1086	R_1 = 0.0358, wR_2 = 0.0815	R_1 = 0.0607, wR_2 = 0.1451	R_1 = 0.0660, wR_2 = 0.1355
Final R indexes [all data]	R_1 = 0.0544, wR_2 = 0.1192	R_1 = 0.0461, wR_2 = 0.0917	R_1 = 0.0737, wR_2 = 0.1595	R_1 = 0.0739, wR_2 = 0.1398
Largest diff. peak/hole / e Å^{-3}	2.70/-1.08	1.60/-1.02	3.37/-2.63	5.20/-4.92

4.- Photostability of the Iridium(III) complexes

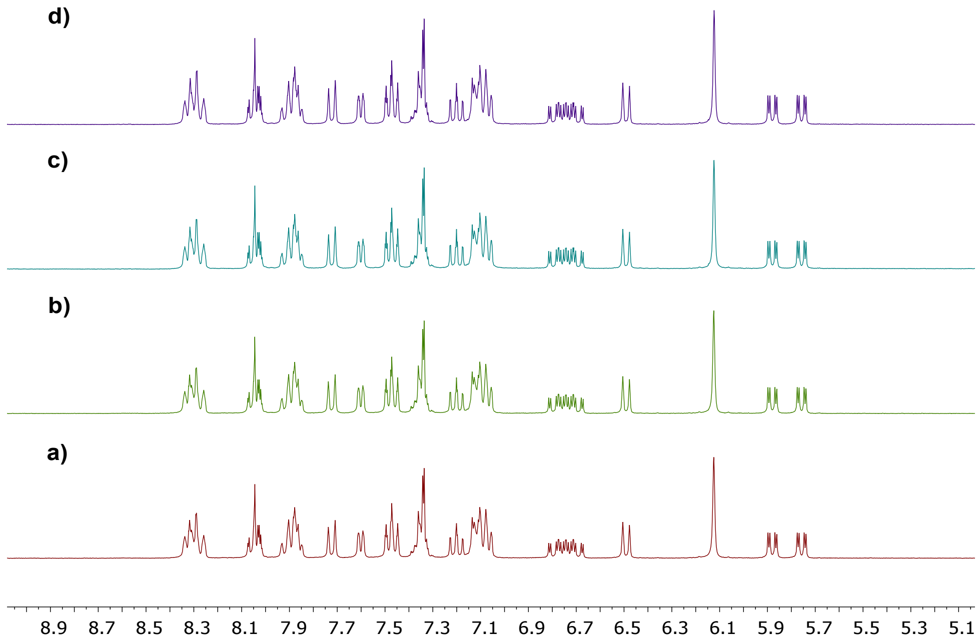

Figure S16. Aromatic Area of the ^{1}H NMR (400 MHz) spectrum of **[Ir3]Cl** in CD_3CN ($1.4\cdot10^{-2}$ M) at 25 °C in the dark after: a) t= 0, b) t= 6 h, c) t= 24 h and d) t= 48 h.

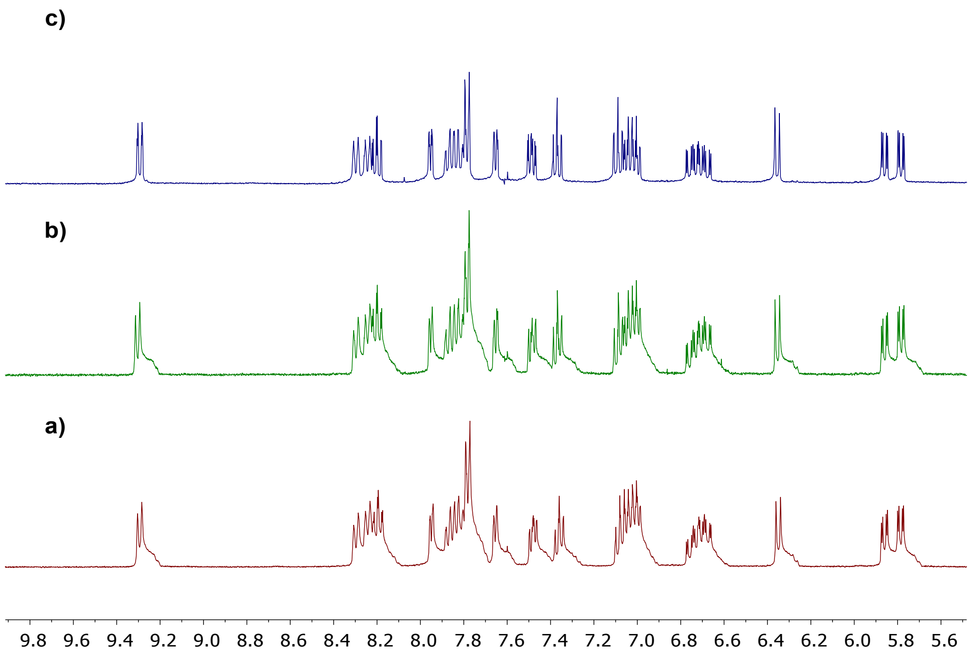

Figure S17. Aromatic Area of ^{1}H NMR (400 MHz) spectra of **[Ir1]Cl** in CD_3CN ($1.4\cdot10^{-2}$ M) at 25 °C after irradiation with Blue LED light (λ_{ir} = 460 nm): a) t= 0, b) t= 6 h and c) t= 24 h.

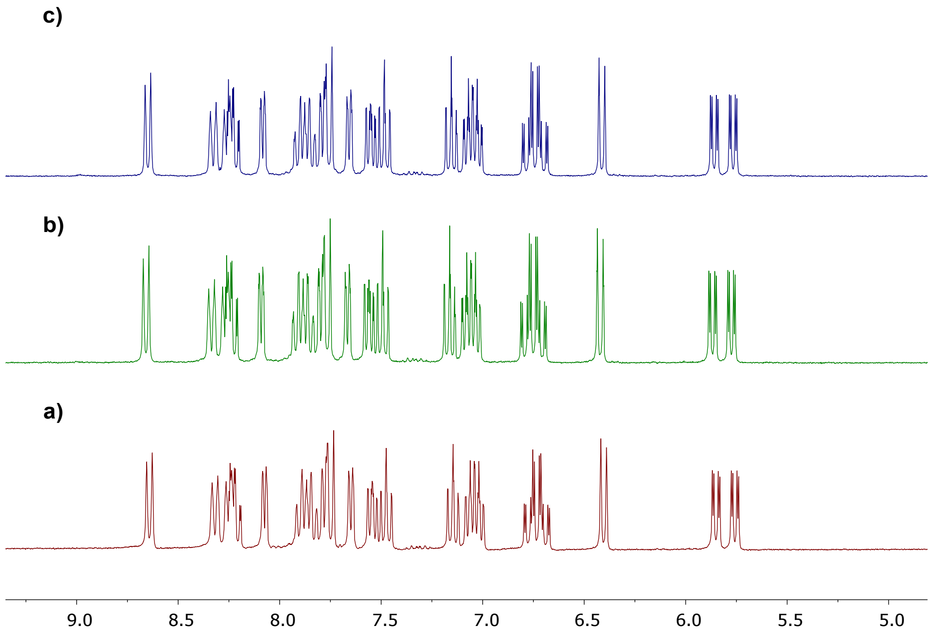

Figure S18. Aromatic Area of ^{1}H NMR (400 MHz) spectra of **[Ir2]Cl** in CD_3CN ($1.4 \cdot 10^{-2}$ M) at 25 °C after irradiation with Blue LED light (λ_{ir} = 460 nm): a) t= 0, b) t= 6 h and c) t= 24 h.

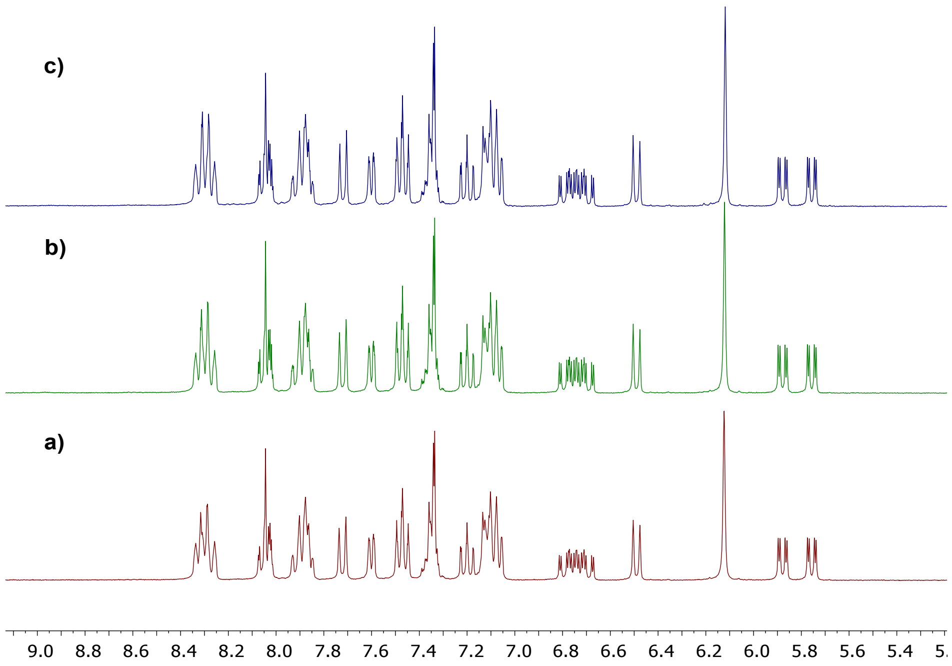

Figure S19. Aromatic Area of ^{1}H NMR (400 MHz) spectra of **[Ir3]Cl** in CD_3CN ($1.4 \cdot 10^{-2}$ M) at 25 °C after irradiation with Blue LED light (λ_{ir} = 460 nm): a) t= 0, b) t= 6 h and c) t= 24 h.

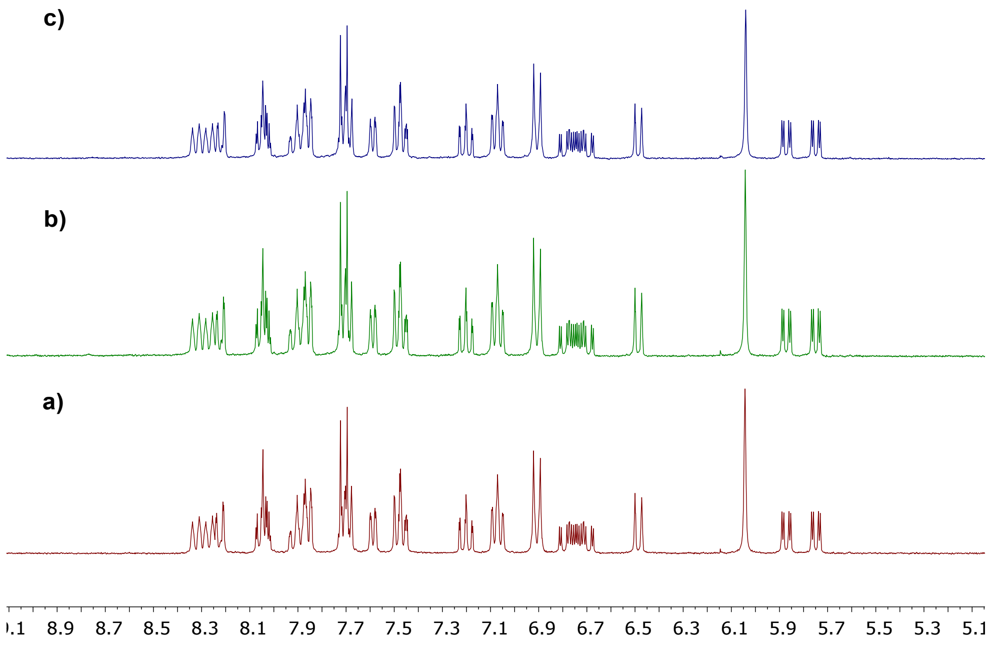

Figure S20. Aromatic Area of ^{1}H NMR (400 MHz) spectra of **[Ir4]Cl** in CD_3CN ($1.4\cdot10^{-2}$ M) at 25 °C after irradiation with Blue LED light (λ_{ir} = 460 nm): a) t= 0, b) t= 6 h and c) t= 24 h.

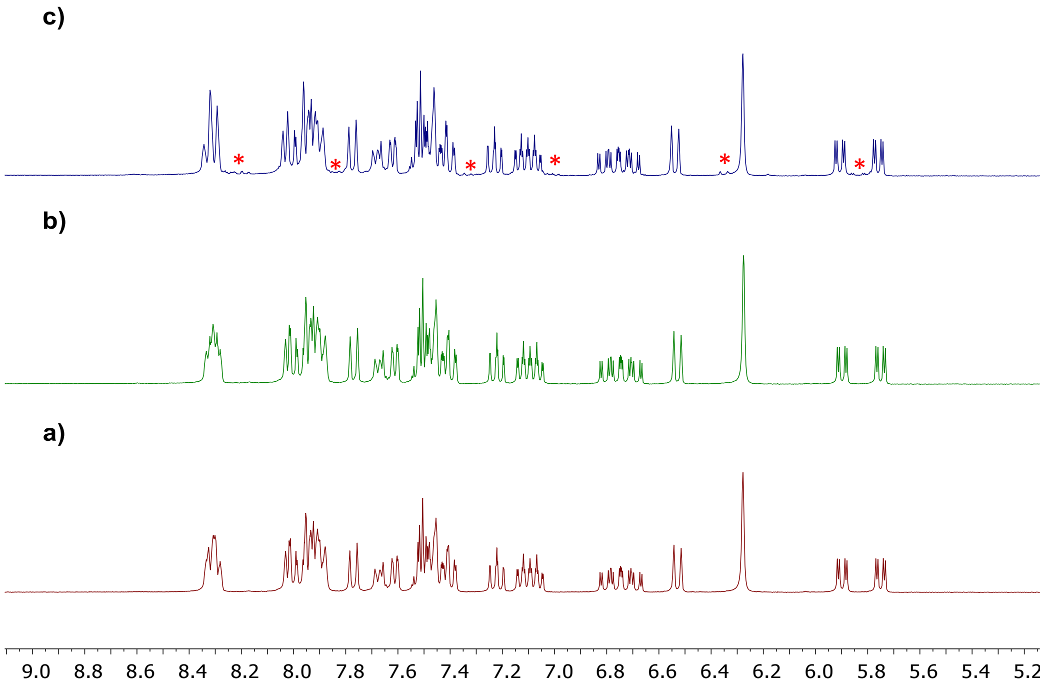

Figure S21. Aromatic Area of ^{1}H NMR (400 MHz) spectra of **[Ir5]Cl** in CD_3CN ($1.4\cdot10^{-2}$ M) at 25 °C after irradiation with Blue LED light (λ_{ir} = 460 nm): a) t= 0, b) t= 6 h and c) t= 24 h. Signals labelled with (*) correspond to the photodegradation product.

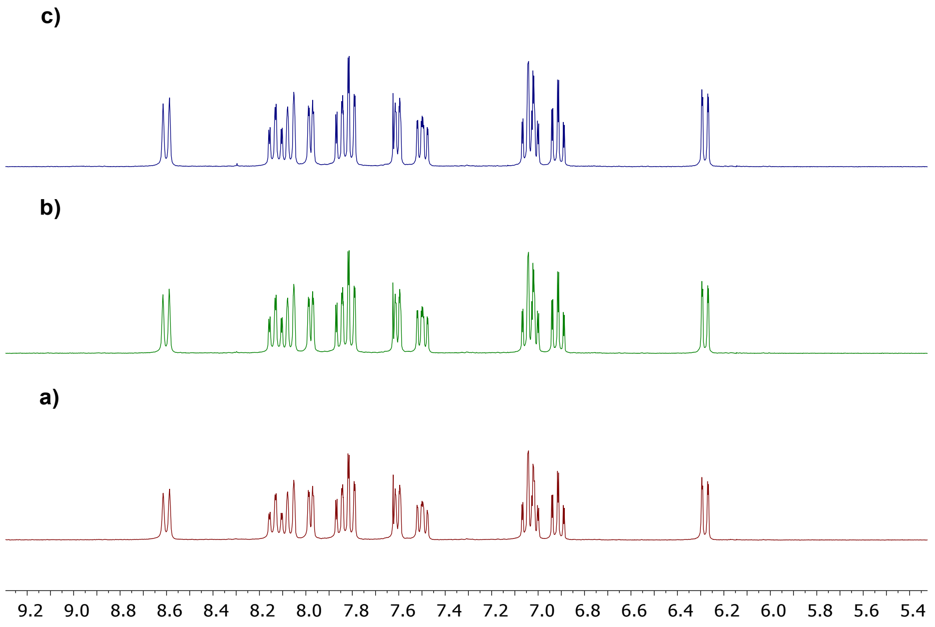

Figure S22a. Aromatic Area of ^{1}H NMR (400 MHz) spectra of **[1]Cl** in CD_3CN ($1.4 \cdot 10^{-2}$ M) at 25 °C after irradiation with Blue LED light (λ_{ir} = 460 nm): a) t= 0, b) t= 6 h and c) t= 24 h.

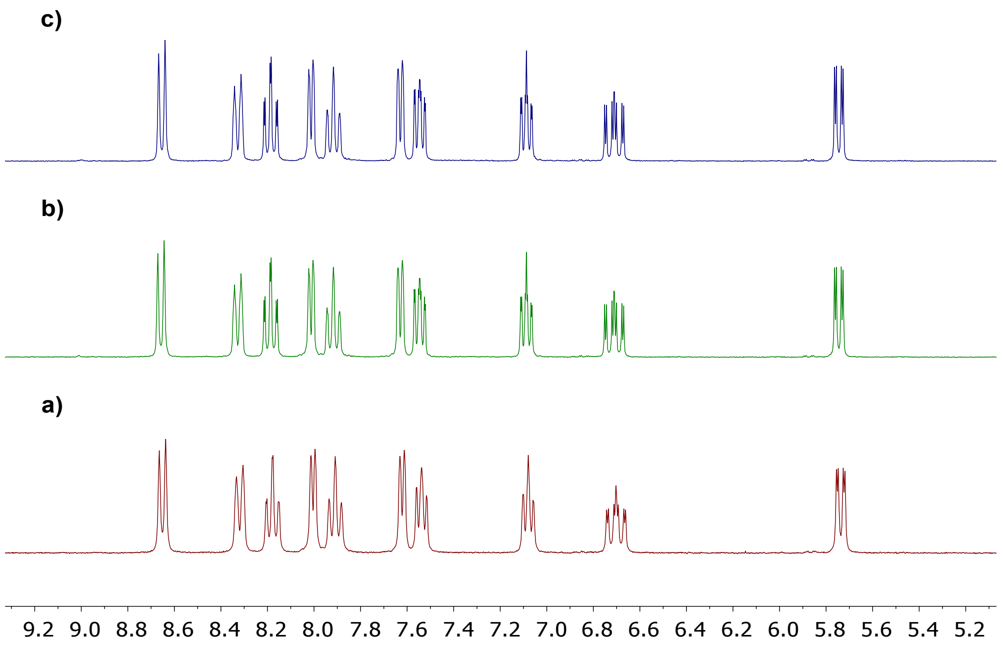

Figure S22b. Aromatic Area of ^{1}H NMR (400 MHz) spectra of **[2]Cl** in CD_3CN ($1.4 \cdot 10^{-2}$ M) at 25 °C after irradiation with Blue LED light (λ_{ir} = 460 nm): a) t= 0, b) t= 6 h and c) t= 24 h.

5.- Theoretical Calculations

Density functional theory (DFT) calculations were carried out with the D.01 revision of the Gaussian 09 package,[10] using the Becke's three-parameter B3LYP exchange-correlation functional,[11,12] together with the 6-31G(d,p) basis set for H, C, N, O, F, and S,[13,14] and the "double-zeta" quality LANL2DZ basis set for the Ru element.[15] The geometries of the singlet ground state (S0) and the lowest-energy triplet state (T1) were fully optimized without imposing any symmetry restriction. The geometries of the triplet states were calculated at the spin-unrestricted UB3LYP level with a spin multiplicity of 3. All the calculations were performed in the presence of the solvent (acetonitrile). Solvent effects were considered within the self-consistent reaction field (SCRF) theory using the SMD keyword that performs a polarized continuum model (PCM) calculation using the solvatation model of Thrular et al.[16] Time-dependent DFT (TD-DFT) calculations of the lowest-lying 15 singlets and triplets were performed in the presence of the solvent at the minimum-energy geometry optimized for the ground state (S_0).

Table S2a.- Topologies and energies (eV) of the MOs of complexes **[Ir1]$^+$** - **[Ir3]$^+$**.

	**[Ir1]$^+$**	**[Ir2]$^+$**	**[Ir3]$^+$**
LUMO +2	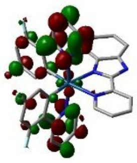 -1.75704003	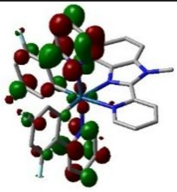 -1.7524141	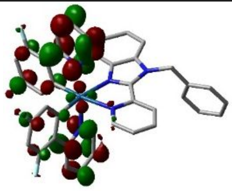 -1.75676792
LUMO +1	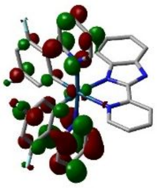 -1.83894634	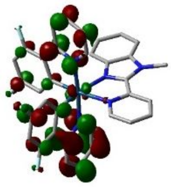 -1.83268772	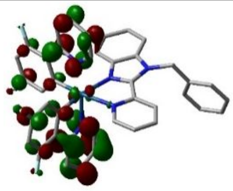 -1.84030691
LUMO	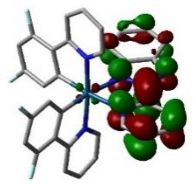 -2.4585499	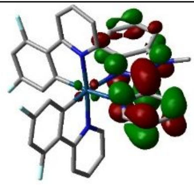 -2.4449442	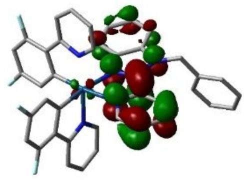 -2.46943446
HOMO	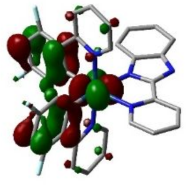 -5.93725515	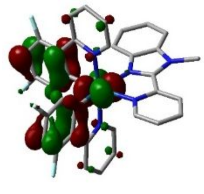 -5.93398978	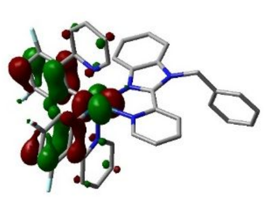 -5.9402484

HOMO -1	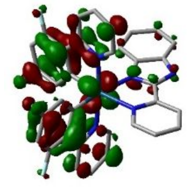<br>-6.39658356	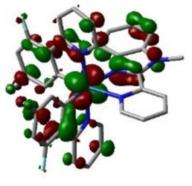<br>-6.3778077	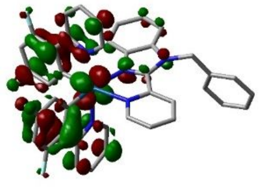<br>-6.39631145
HOMO -2	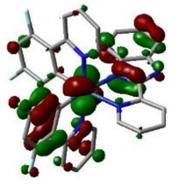<br>-6.4847485	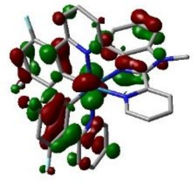<br>-6.45481596	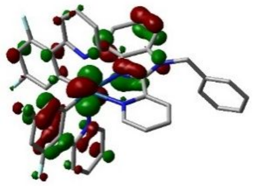<br>-6.47386394

Table S2b.- Topologies and energies (eV) of the MOs of complexes **[Ir4]**$^+$ - **[Ir5]**$^+$, **[Ir(ppy)$_2$(bpy)]**$^+$, **[1]**$^+$, and **[Ir(dfppy)$_2$(bpy)]**$^+$, **[2]**$^+$.

	**[Ir4]**$^+$	**[Ir5]**$^+$	**[1]**$^+$	**[2]**$^+$
LUMO +4	-	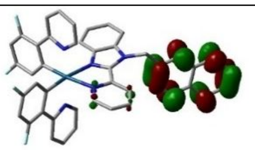<br>-1.35431133	-	
LUMO +2	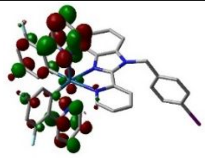<br>-1.76302654	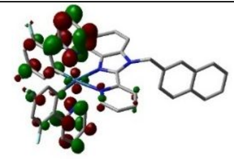<br>-1.764115	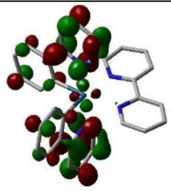<br>-1.67186835	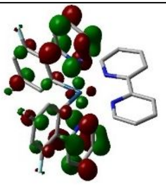<br>-1.77418321
LUMO +1	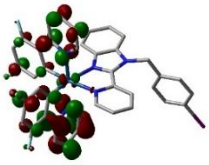<br>-1.84520497	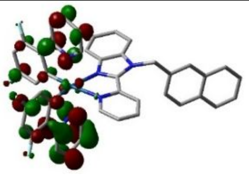<br>-1.84302805	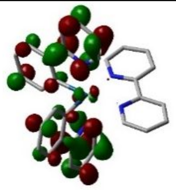<br>-1.75948906	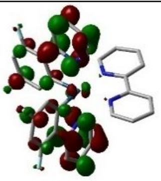<br>-1.85608953

LUMO	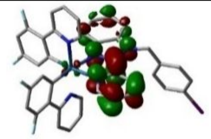 -2.49392472	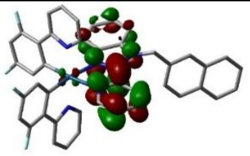 -2.48521707	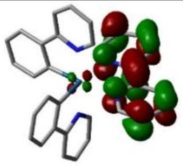 -2.41419532	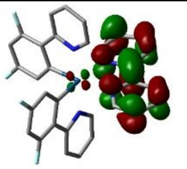 -2.49229203
HOMO	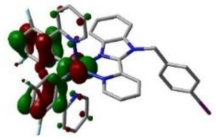 -5.9459628	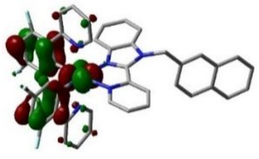 -5.93997629	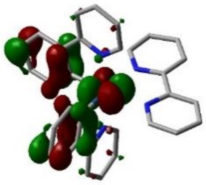 -5.64609318	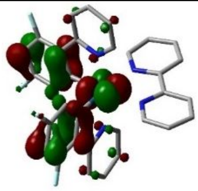 -5.97045305
HOMO -1	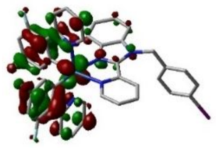 -6.40175373	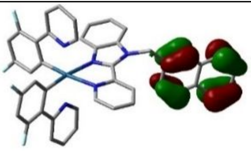 -6.11657827	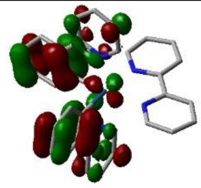 -6.28991488	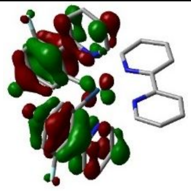 -6.42569976
HOMO -2	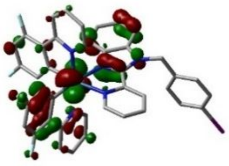 -6.48583695	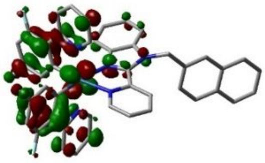 -6.39794413	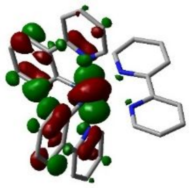 -6.42950936	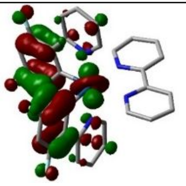 -6.53291267
HOMO -3	-	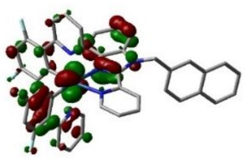 -6.47794565	-	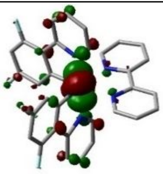 -6.75958363

Table S3. Lowest triplet excited states calculated at the TD-DFT B3LYP/(6-31G(d,p)+LANL2DZ) level for complexes **[Ir1]Cl - [Ir5]Cl**, **[Ir(phpy)$_2$(bpy)]$^+$**, **[1]$^+$**, and **[Ir(dfphpy)$_2$(bpy)]$^+$**, **[2]$^+$**, in acetonitrile solution. Vertical excitation energies (E), dominant monoexcitations with contributions (within parentheses) greater than 15%, nature of the electronic transition and description of the excited state are summarized. H and L denote HOMO and LUMO, respectively.

Compound	State	E (eV; nm) / *f*	Monoexcitations	Nature	Description
**[Ir1]$^+$**	S_1	2.80; 442 / 0.0002	H→L (100)	$d_\pi(Ir) + \pi_{dfppy} \rightarrow \pi^*_{L1}$	^{1}MLCT/^{1}LLCT
	S_2	3.32; 374 / 0.0339	H-2→L (46)<br>H-1→L (47)	$d_\pi(Ir) + \pi_{dfppy} + \pi_{L1} \rightarrow \pi^*_{L1}$<br>$d_\pi(Ir) + \pi_{dfppy} + \pi_{L1} \rightarrow \pi^*_{L1}$	^{1}MLCT/^{1}LLCT/^{1}LC<br>^{1}MLCT/^{1}LLCT/^{1}LC
	S_3	3.33; 372 / 0.0504	H→L+1 (100)	$d_\pi(Ir) + \pi_{dfppy} \rightarrow \pi^*_{dfbpy}$	^{1}MLCT/^{1}LC
	T_1	2.69; 461 / ----	H-2→L (32)<br>H-1→L (15)<br>H→L (34)	$d_\pi(Ir) + \pi_{dfppy} + \pi_{L1} \rightarrow \pi^*_{L1}$<br>$d_\pi(Ir) + \pi_{dfppy} + \pi_{L1} \rightarrow \pi^*_{L1}$<br>$d_\pi(Ir) + \pi_{dfppy} \rightarrow \pi^*_{L1}$	^{3}MLCT/^{3}LLCT/^{3}LC<br>^{3}MLCT/^{3}LLCT/^{3}LC<br>^{3}MLCT/^{3}LLCT
	T_2	2.82; 439 / ----	H-2→L (15)<br>H→L (72)	$d_\pi(Ir) + \pi_{dfppy} + \pi_{L1} \rightarrow \pi^*_{L1}$<br>$d_\pi(Ir) + \pi_{dfppy} \rightarrow \pi^*_{L1}$	^{3}MLCT/^{3}LLCT/^{3}LC<br>^{3}MLCT/^{3}LLCT
	T_3	2.89; 429 / ----	H-1→L+2 (21)<br>H→L+1 (60)	$d_\pi(Ir) + \pi_{dfppy} + \pi_{L1} \rightarrow \pi^*_{dfbpy}$<br>$d_\pi(Ir) + \pi_{dfppy} \rightarrow \pi^*_{dfbpy}$	^{3}MLCT/^{3}LLCT/^{3}LC<br>^{3}MLCT/^{3}LC
**[Ir2]$^+$**	S_1	2.81; 441 /0.0003	H→L (100)	$d_\pi(Ir) + \pi_{dfppy} \rightarrow \pi^*_{L2}$	^{1}MLCT/^{1}LLCT
	S_2	3.30; 376 /0.0374	H-2→L (28)<br>H-1→L (65)	$d_\pi(Ir) + \pi_{dfppy} + \pi_{L2} \rightarrow \pi^*_{L2}$<br>$d_\pi(Ir) + \pi_{dfppy} + \pi_{L2} \rightarrow \pi^*_{L2}$	^{1}MLCT/^{1}LLCT/^{1}LC<br>^{1}MLCT/^{1}LLCT/^{1}LC
	S_3	3.34; 372 /0.0496	H→L+1 (100)	$d_\pi(Ir) + \pi_{dfppy} \rightarrow \pi^*_{dfbpy}$	^{1}MLCT/^{1}LC
	T_1	2.67; 464 / ----	H-2→L (28)<br>H-1→L (32)<br>H→L (28)	$d_\pi(Ir) + \pi_{dfppy} + \pi_{L2} \rightarrow \pi^*_{L2}$<br>$d_\pi(Ir) + \pi_{dfppy} + \pi_{L2} \rightarrow \pi^*_{L2}$<br>$d_\pi(Ir) + \pi_{dfppy} \rightarrow \pi^*_{L2}$	^{3}MLCT/^{3}LLCT/^{3}LC<br>^{3}MLCT/^{3}LLCT/^{3}LC<br>^{3}MLCT/^{3}LLCT
	T_2	2.82; 439 / ----	H→L (77)	$d_\pi(Ir) + \pi_{dfppy} \rightarrow \pi^*_{L2}$	^{3}MLCT/^{3}LLCT
	T_3	2.90; 428 / ----	H-1→L+2 (16)<br>H→L+1 (58)	$d_\pi(Ir) + \pi_{dfppy} + \pi_{L2} \rightarrow \pi^*_{dfbpy}$<br>$d_\pi(Ir) + \pi_{dfppy} \rightarrow \pi^*_{dfbpy}$	^{3}MLCT/^{3}LLCT/^{3}LC<br>^{3}MLCT/^{3}LC
**[Ir3]$^+$**	S_1	2.79;444 / 0.0006	H→L (100)	$d_\pi(Ir) + \pi_{dfppy} \rightarrow \pi^*_{L3}$	^{1}MLCT/^{1}LLCT
	S_2	3.30; 376 / 0.0362	H-2→L (48)<br>H-1→L (48)	$d_\pi(Ir) + \pi_{dfppy} + \pi_{L3} \rightarrow \pi^*_{L3}$<br>$d_\pi(Ir) + \pi_{dfppy} + \pi_{L3} \rightarrow \pi^*_{L3}$	^{1}MLCT/^{1}LLCT/^{1}LC<br>^{1}MLCT/^{1}LLCT/^{1}LC
	S_3	3.34; 372 / 0.0474	H→L+1 (100)	$d_\pi(Ir) + \pi_{dfppy} \rightarrow \pi^*_{dfbpy}$	^{1}MLCT/^{1}LC
	T_1	2.66; 465 / ----	H-2→L (32)<br>H-1→L (16)<br>H→L (41)	$d_\pi(Ir) + \pi_{dfppy} + \pi_{L3} \rightarrow \pi^*_{L3}$<br>$d_\pi(Ir) + \pi_{dfppy} + \pi_{L3} \rightarrow \pi^*_{L3}$<br>$d_\pi(Ir) + \pi_{dfppy} \rightarrow \pi^*_{L3}$	^{3}MLCT/^{3}LLCT/^{3}LC<br>^{3}MLCT/^{3}LLCT/^{3}LC<br>^{3}MLCT/^{3}LLCT
	T_2	2.81; 440 / ----	H-2→L (19)<br>H→L (65)	$d_\pi(Ir) + \pi_{dfppy} + \pi_{L3} \rightarrow \pi^*_{L3}$<br>$d_\pi(Ir) + \pi_{dfppy} \rightarrow \pi^*_{L3}$	^{3}MLCT/^{3}LLCT/^{3}LC<br>^{3}MLCT/^{3}LLCT
	T_3	2.90; 428 / ----	H-1→L+2 (20)<br>H→L+1 (56)	$d_\pi(Ir) + \pi_{dfppy} + \pi_{L3} \rightarrow \pi^*_{dfbpy}$<br>$d_\pi(Ir) + \pi_{dfppy} \rightarrow \pi^*_{dfbpy}$	^{3}MLCT/^{3}LLCT/^{3}LC<br>^{3}MLCT/^{3}LC
**[Ir4]$^+$**	S_1	2.77; 447 / 0.0004	H→L (100)	$d_\pi(Ir) + \pi_{dfppy} \rightarrow \pi^*_{L4}$	^{3}MLCT/^{3}LLCT
	S_2	3.28; 378 / 0.0364	H-2→L (49)<br>H-1→L (46)	$d_\pi(Ir) + \pi_{dfppy} + \pi_{L4} \rightarrow \pi^*_{L4}$<br>$d_\pi(Ir) + \pi_{dfppy} + \pi_{L4} \rightarrow \pi^*_{L4}$	^{3}MLCT/^{3}LLCT/^{3}LC<br>^{3}MLCT/^{3}LLCT/^{3}LC
	S_3	3.34; 372 / 0.0472	H→L+1 (100)	$d_\pi(Ir) + \pi_{dfppy} \rightarrow \pi^*_{dfbpy}$	^{3}MLCT/^{3}LC
	T_1	2.65; 467 / ----	H-2→L (30)<br>H→L (42)	$d_\pi(Ir) + \pi_{dfppy} + \pi_{L4} \rightarrow \pi^*_{L4}$<br>$d_\pi(Ir) + \pi_{dfppy} \rightarrow \pi^*_{L4}$	^{3}MLCT/^{3}LLCT/^{3}LC<br>^{3}MLCT/^{3}LLCT

	T_2	2.80; 443 / ----	H-2→L (21)<br>H→L (67)	d_π(Ir) + π_{dfppy} + π_{L4}→ π^*_{L4}<br>d_π(Ir) + π_{dfppy} → π^*_{L4}	^{3}MLCT/^{3}LLCT/^{3}LC<br>^{3}MLCT/^{3}LLCT
	T_3	2.90; 428 / ----	H-1→L+2 (20)<br>H→L+1 (58)	d_π(Ir) + π_{dfppy} + π_{L4}→ π^*_{dfbpy}<br>d_π(Ir) + π_{dfppy} → π^*_{dfbpy}	^{3}MLCT/^{3}LLCT/^{3}LC<br>^{3}MLCT/^{3}LC
**[Ir5]$^+$**	S_1	2.77; 447 / 0.0007	H→L (100)	d_π(Ir) + π_{dfppy} → π^*_{L5}	^{1}MLCT/^{1}LLCT
	S_2	3.18; 390 / 0.0011	H-1→L (100)	π_{L5} → π^*_{L5}	^{1}LC
	S_3	3.28; 378 / 0.0390	H-3→L (44)<br>H-2→L (49)	d_π(Ir) + π_{dfppy} + π_{L5} → π^*_{L5}<br>d_π(Ir) + π_{dfppy} + π_{L5} → π^*_{L5}	^{1}MLCT/^{1}LLCT/^{1}LC<br>^{1}MLCT/^{1}LLCT/^{1}LC
	T_1	2.65; 468 / ----	H-3→L (30)<br>H-2→L (16)<br>H→L (43)	d_π(Ir) + π_{dfppy} + π_{L5} →π^*_{L5}<br>d_π(Ir) + π_{dfppy} + π_{L5} → π^*_{L5}<br>d_π(Ir) + π_{dfppy} → π^*_{L5}	^{3}MLCT/^{3}LLCT/^{3}LC<br>^{3}MLCT/^{3}LLCT/^{3}LC<br>^{3}MLCT/^{3}LLCT
	T_2	2.70; 460 / ----	H-1→L+4 (81)	π_{L5} → π^*_{L5}	^{3}LC
	T_3	2.80; 443 / ----	H-3→L (20)<br>H→L (61)	d_π(Ir) + π_{dfppy} + π_{L5} → π^*_{L5}<br>d_π(Ir) + π_{dfppy} → π^*_{L5}	^{3}MLCT/^{3}LLCT/^{3}LC<br>^{3}MLCT/^{3}LLCT
**[Ir(ppy)$_2$(bpy)]$^+$, [1]$^+$**	S_1	2.54; 487 / 0.0002	H→L (100)	d_π(Ir) + π_{ppy} → π^*_{bpy}	^{1}MLCT/^{1}LLCT
	S_2	3.14; 395 / 0.0595	H→L+1 (100)	d_π(Ir) + π_{ppy} → π^*_{ppy}	^{1}MLCT/^{1}LC
	S_3	3.25; 382 / 0.0009	H→L+2 (97)	d_π(Ir) + π_{ppy} → π^*_{ppy}	^{1}MLCT/^{1}LC
	T_1	2.52; 493 / ----	H→L (100)	d_π(Ir) + π_{ppy} → π^*_{bpy}	^{3}MLCT/^{3}LLCT
	T_2	2.76; 449 / ----	H-1→L+2 (19)<br>H→L+1 (74)	d_π(Ir) + π_{ppy} → π^*_{ppy}<br>d_π(Ir) + π_{ppy} → π^*_{ppy}	^{3}MLCT/^{3}LC<br>^{3}MLCT/^{3}LC
	T_3	2.81; 441 / ----	H-1→L+1 (31)<br>H→L+2 (62)	d_π(Ir) + π_{ppy} → π^*_{ppy}<br>d_π(Ir) + π_{ppy} → π^*_{ppy}	^{3}MLCT/^{3}LC<br>^{3}MLCT/^{3}LC
**[Ir(dfppy)$_2$(bpy)]$^+$, [2]$^+$**	S_1	2.79; 444 / 0.0002	H→L (100)	d_π(Ir) + π_{dfppy} → π^*_{bpy}	^{1}MLCT/^{1}LLCT
	S_2	3.35; 370 / 0.0549	H→L+1 (100)	d_π(Ir) + π_{dfppy} → π^*_{dfppy}	^{1}MLCT/^{1}LC
	S_3	3.39; 366 / 0.0008	H-3→L (45)<br>H-2→L (55)	d_π(Ir) → π^*_{bpy}<br>d_π(Ir) + π_{dfppy} → π^*_{bpy}	^{1}MLCT<br>^{1}MLCT/^{1}LLCT
	T_1	2.76; 449 / ----	H→L (98)	d_π(Ir) + π_{dfppy} → π^*_{bpy}	^{3}MLCT/^{3}LLCT
	T_2	2.90; 428 / ----	H-1→L+2 (28)<br>H→L+1 (56)	d_π(Ir) + π_{dfppy} → π^*_{dfppy}<br>d_π(Ir) + π_{dfppy} → π^*_{dfppy}	^{3}MLCT/^{3}LC<br>^{3}MLCT/^{3}LC
	T_3	2.93; 423 / ----	H-1→L+1 (39)<br>H→L+2 (45)	d_π(Ir) + π_{dfppy} → π^*_{dfppy}<br>d_π(Ir) + π_{dfppy} → π^*_{dfppy}	^{3}MLCT/^{3}LC<br>^{3}MLCT/^{3}LC

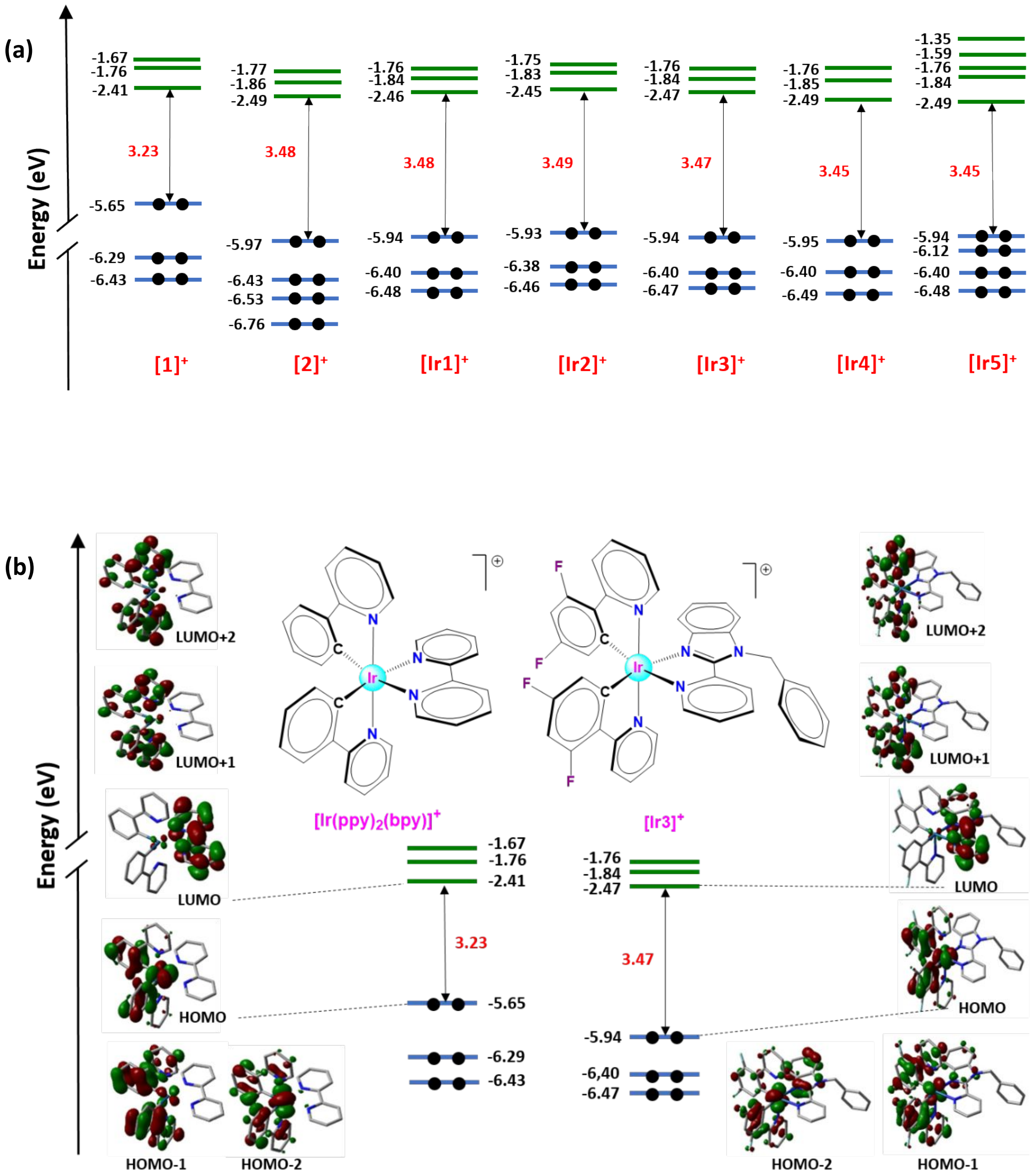

Figure S23. (a) Representation showing the energies calculated for the frontier molecular orbitals of **[Ir1]**$^{+}$ - **[Ir5]**$^{+}$, **[1]**$^{+}$ and **[2]**$^{+}$. **(b)** Schematic representation showing the energies and the isovalue contour plots calculated for the frontier molecular orbitals of **[1]**$^{+}$ and **[Ir3]**$^{+}$.

6.-Electrochemical measurements.

Electrochemical measurements were performed using a portable potentiostat/galvanostat PalmSens[3] (PalmSens) equipment controlled by the software PSTrace4 Version 4.4.2. All experiments were carried out using a three-electrode cell with a glassy carbon-disc (diameter = 3 mm) as the working electrode, a platinum-wire as the auxiliary electrode, and a Ag/AgCl (MF-2052 BASi) reference electrode separated from the bulk solution by a Vycor™ frit. Oxygen was removed from the solution by bubbling argon for 5 minutes and keeping the current of argon along the whole experiment. The measurements were recorded for acetonitrile solutions of the Ru(II) complexes ($5{\cdot}\times 10^{-4}$ M) in the presence of $[n\mathrm{Bu_4N}][\mathrm{PF_6}]$ (0.1 M) as the supporting electrolyte by cyclic voltammetry (CV) at a scan rate of 100 mV s^{-1} in a clockwise direction. Ferrocene was added at the end of all the experiments as the internal reference in order to refer the potentials to the redox pair ferrocenium/ferrocene (Fc^+/Fc) under the conditions of our experiments. The potential experimentally determined for the redox couple Fc^+/Fc was $E°_{1/2} = 0.443\pm0.005$ V vs. Ag/AgCl. Therefore, the experimental redox potentials were calculated from the corresponding voltammograms as:

E° (vs AgCl/Ag) = (E_{ap} + E_{cp})/2, for reversible peaks where E_{ap} and E_{cp} stand for anodic and cathodic peak potentials, respectively. However, for irreversible peaks, the potentials were calculated as either the E_{ap} maximum or E_{cp} minimum.

E° (vs Fc^+/Fc) = E° (vs AgCl/Ag) – 0.443, for potential values reported in reference to the (Fc^+/Fc) redox couple.

E° (vs SCE) = E° (vs Fc^+/Fc) + 0.404, for potential values reported in reference to the saturated calomel electrode (SCE) and having into account that $Eº_{1/2}$ (Fc^+/Fc) = 0.404 V vs SCE according to the literature.[17]

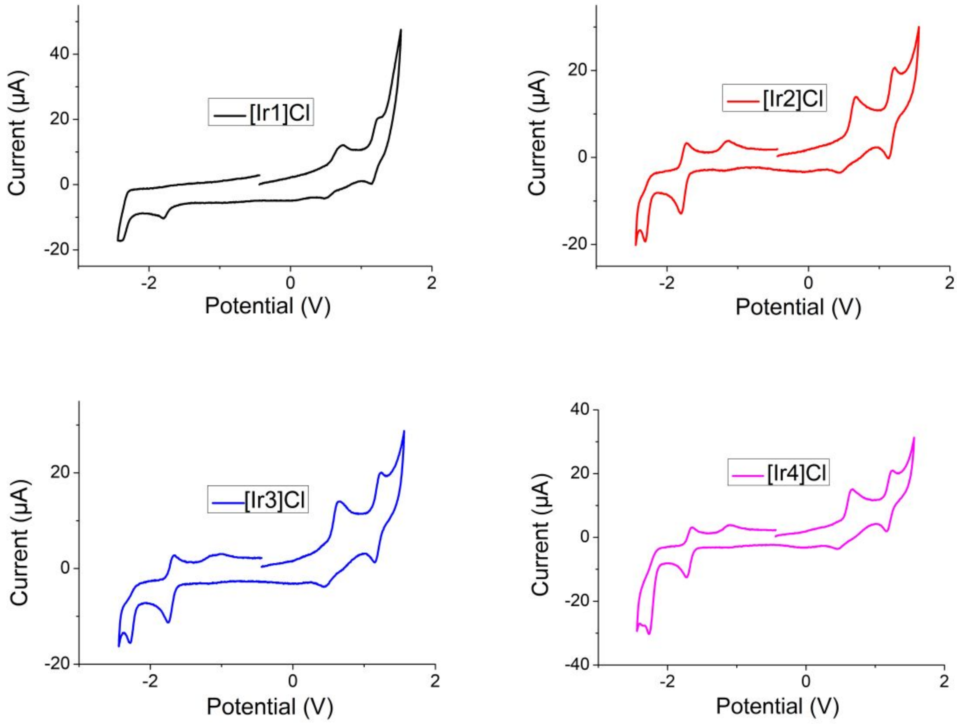

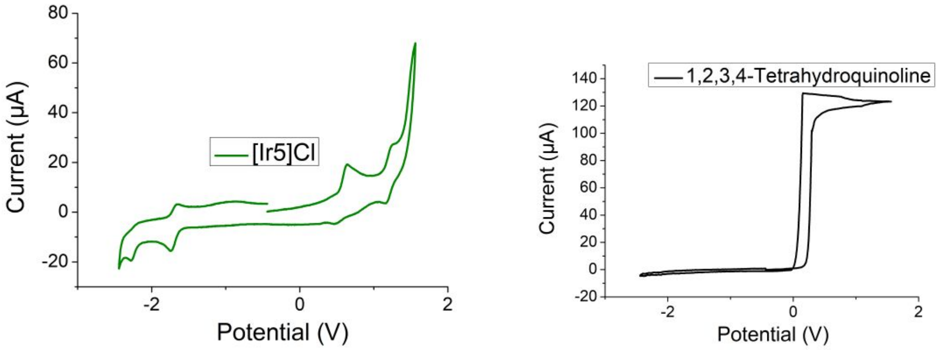

Figure S24. Cyclic voltammograms of the iridium complexes and 1,2,3,4-tetrahydroquinoline versus Fc^+/Fc.

Table S4. Redox Potentials for the excited states versus Fc^+/Fc. Data for **[1]PF$_6$** reported by E. Zysman-Colman et al.[47,49] Data for **[2]PF$_6$** reported by De Cola.[48]

Complex	$E_{1/2}(Ir^{IV}/Ir^{III})$	$E_{1/2}(Ir^{III}/Ir^{II})$	$E_{1/2}(Ir^{III*}/Ir^{III})$	$E_{1/2}(Ir^{IV}/Ir^{III*})$	$E_{1/2}(Ir^{III*}/Ir^{II})$
**[1](PF$_6$)**	+0.87	-1.78	2.06 eV (602 nm)	-1.19	+0.28
**[2](PF$_6$)**	+1.22	-1.65	2.32 eV (534 nm)	-1.10	+0.67
**[Ir1]Cl**	+1.22	-1.79	2.38 eV (522 nm)	-1.16	+0.59
**[Ir2]Cl**	+1.19	-1.76	2.30 eV (539 nm)	-1.11	+0.54
**[Ir3]Cl**	+1.21	-1.69	2.28 eV (544 nm)	-1.07	+0.59
**[Ir4]Cl**	+1.22	-1.67	2.28 eV (544 nm)	-1.06	+0.61
**[Ir5]Cl**	+1.22	-1.69	2.27 eV (546 nm)	-1.05	+0.58

[a]All potential are given in volts versus Fc^+/Fc. $E_{1/2}(Ir^{IV}/Ir^{III*}) = E_{1/2}(Ir^{IV}/Ir^{III}) - E_{1/2}(Ir^{III*}/Ir^{III})$ and $E_{1/2}(Ir^{III*}/Ir^{II}) = E_{1/2}(Ir^{III}/Ir^{II}) + E_{1/2}(Ir^{III*}/Ir^{III})$. Data for **[1]PF$_6$** and **[2]PF$_6$** are given in acetonitrile vs Fc^+/Fc (calculated from the original work through: V(Fc^+/Fc) = V(SCE) – 0.404) (Ref: Ko, C. *Catal. Sci. Technol.* **2021**, *11* (2), 556–562).

7.- Procedure for the photocatalytic oxidation of indolines and tetrahydroquinolines

In a septum-capped test tube the substrate (5 μmol in solution of CH_3CN), the PC (Photocatalyst = in solution of CH_3CN and the required concentration depending on the experiment), and additional CH_3CN to provide the desired final concentration of substrate (10 mM in 0.5 mL), were added. The system was purged with O_2 or N_2 until atmosphere saturation and irradiated with Blue LED light (λ= 460 nm, 24W) at room temperature during the required time (24-48 h). Then, an aliquot (100 μL) of the reaction mixture was diluted in CD_3CN (400 μL) and the mixture was analysed by ^1H NMR to determine the conversion.

For the isolation of indoline and tetrahydroquinoline the procedure was adapted:

Scale up synthesis of Indole 2a:

In a septum-capped round bottom flask the indoline **1a** (8.4 mmol in solution of CH_3CN), the PC (**[Ir3]Cl;** 0.0252 mmol in solution of CH_3CN), and additional CH_3CN to provide the desired final concentration of substrate (168 mM in 50 mL) were placed. The system was purged with O_2 until atmosphere saturation

and stirred under irradiation with Blue LED light (λ= 460 nm, 24W) at room temperature during 75 hours The product was isolated filtering the crude solution through a silica pad, using acetonitrile like mobile phase. Then the product was dried under vacuum to constant weight. The ^{1}H NMR was recorded in $CDCl_3$. **2a** 0.9325 g, 0.0080 mol. Yield: 95%.

Scale up synthesis of quinolone 4a:

In a septum-capped round bottom flask the tetrahydroquinoline **3a** (7.5 mmol in solution of CH_3CN), the PC (**[Ir4]Cl;** 0.0525 mmol in solution of CH_3CN), and additional CH_3CN to provide the desired final concentration of substrate (150 mM in 50 mL) were placed. The system was purged with O_2 until atmosphere saturation and stirred under irradiation with Blue LED light (λ= 460 nm, 24W) at room temperature during 75 hours. The product was isolated filtering the crude solution through a silica pad, using acetonitrile like mobile phase. Then the product was dried under vacuum to constant weight. The ^{1}H NMR was recorded in $CDCl_3$. **4a** 0.8534 g, 0.0066 mol. Yield: 88%.

Table S5. Substrate Scope for the photooxidation of indolines[a]

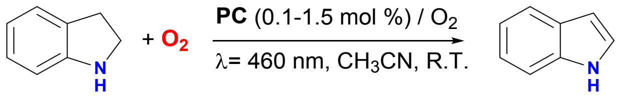

1a 2a

Entry	Substrate	Product	Yield (%) / [Photocatalyst (mol%)]
1	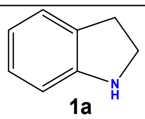 1a	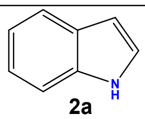 2a	62 [0.1]<br>83 [0.2]<br>**100 [0.3]**
2	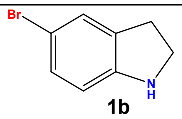 1b	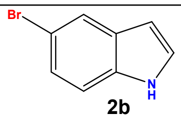 2b	36 [0.3]<br>**62 [1]**
3	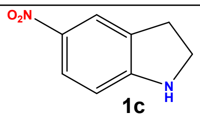 1c	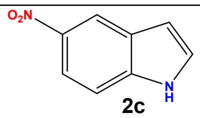 2c	0 [0.3]<br>**0 [1.5]**
4	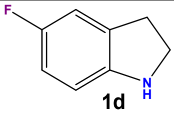 1d	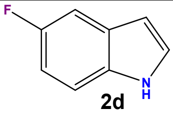 2d	56 [0.3]<br>63 [0.4]<br>**94 [1]**
5	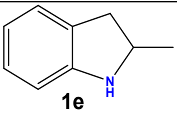 1e	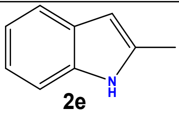 2e	**100 [0.3]**
6	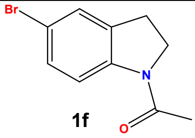 1f	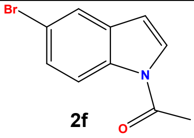 2f	0 [0.3]<br>**0 [1.5]**

7	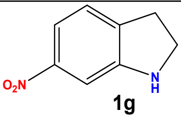 1g	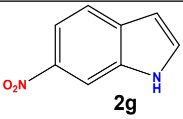 2g	<5 [0.3]<br>**20 [1.5]**

[a]<u>Reaction Conditions:</u> indoline (10 mM), acetonitrile (0.5 mL), O_2 (balloon, 1 atm), blue LED light (460 nm), room temperature for 24 hours. The yields (red) were experimentally determined from ^{1}H NMR integration of the corresponding reaction crudes.

Table S1. Substrate Scope for the photooxidation of quinolines[a]

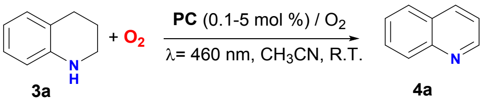

Entry	Substrate	Product	Yield (%) / [Photocatalyst (mol%)]
1	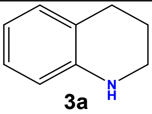 3a	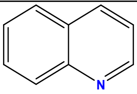 4a	70 [0.1]<br>77 [0.3]<br>86 [0.5]<br>**100 [0.7]**
2	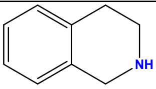 3b	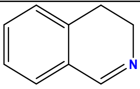 4b	**100 [0.7]**
3	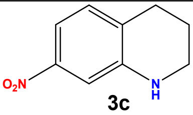 3c	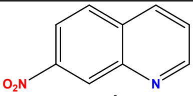 4c	0 [0.7]<br>40 / **>99[b] [5]**
4	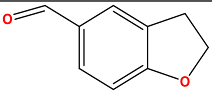 3d	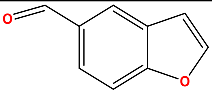 4d	0 [0.7]<br>**0 [5]**
5	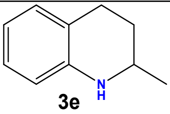 3e	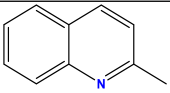 4e	**100 [0.7]**
6	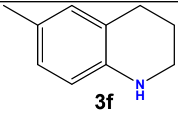 3f	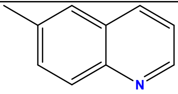 4f	29 [0.7]<br>**61 [1.5]**
7	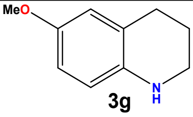 3g	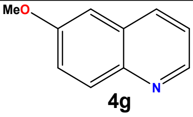 4g	43 [0.7]<br>**94 [1.5]**
8	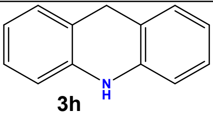 3h	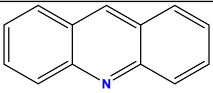 4h	**100 [0.7]**
9	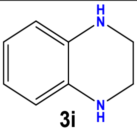 3i	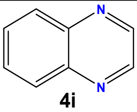 4i	46 [0.7]<br>**52 [1.5]**

10	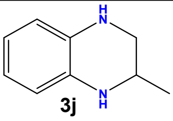 3j	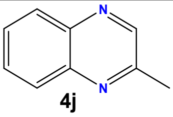 4j	35 [0.7]<br>43 [1.5]<br>**54 [3]**
11	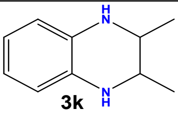 3k	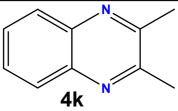 4k	33 [0.7]<br>44 [1.5]<br>51 [3]<br>**62 [5]**

[a]Reaction Conditions: indoline (10 mM), acetonitrile (0.5 mL), O_2 (balloon, 1 atm), blue LED light (460 nm), room temperature for 24 hours. The yields (red) were experimentally determined from ^1H NMR integration of the corresponding reaction crudes. [b]Reaction time of 48 hours.

8.- ^1H NMR spectra and characterization of the crudes and isolated products

^1H NMR spectra and characterization of the crude indoles

2a: Indole

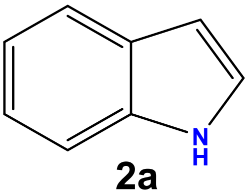

^1H NMR (400 MHz, Acetonitrile-d_3) δ 7.55 (d, *J* = 7.9 Hz, 1H), 7.41 (dd, *J* = 8.2, 0.9 Hz, 1H), 7.24 – 7.19 (m, 1H), 7.13 – 7.07 (m, 1H), 7.01 (ddd, *J* = 8.0, 7.0, 1.0 Hz, 1H), 6.46 – 6.41 (m, 1H) ppm. **HR ESI+ MS (CH_2Cl_2):** [M+H$^+$] calcd. for $[C_8H_8N]^+$ 118.0651, found 118.0648.

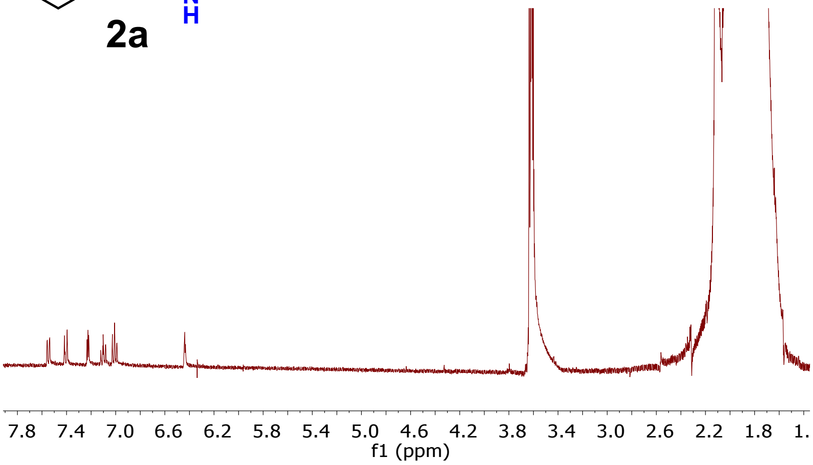

Figure S25. ^1H NMR spectrum in CD_3CN for the crude **2a** of the photocatalytic oxidation of indoline (**1a,** 10 mM) in the presence of the photocatalyst **[Ir3]Cl** (0.3 mol%) in CH_3CN under O_2 and blue LED irradiation (λ_{exc} = 460 nm) at room temperature.

2b: 5-Bromoindole

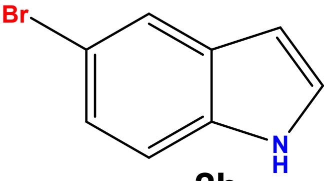

2b

^{1}H NMR (400 MHz, Acetonitrile-*d*$_3$) δ 7.71 (s, 1H), 7.35 (d, *J* = 8.5 Hz, 1H), 7.27 (t, *J* = 2.8 Hz, 1H), 7.21 (d, *J* = 2.0 Hz, 1H), 6.42 (dd, *J* = 2.3, 1.2 Hz, 1H) ppm. **HR ESI+ MS (CH_2Cl_2):** [M+H$^+$] calcd. for $[C_8H_7BrN]^+$ 195.9756, found 195.9747.

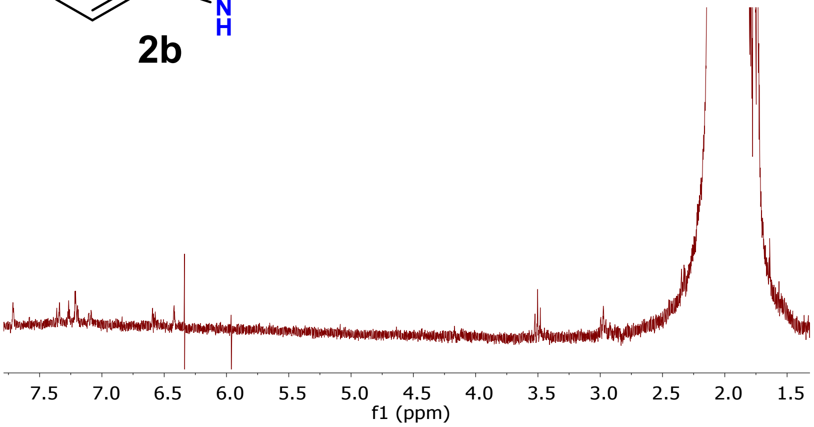

Figure S26. ^{1}H NMR spectrum in CD_3CN for the crude **2b** of the photocatalytic oxidation of indoline (**1b,** 10 mM) in the presence of the photocatalyst **[Ir3]Cl** (1 mol%) in CH_3CN under O_2 and blue LED irradiation (λ_{exc} = 460 nm) at room temperature.

2d: 5-Fluoroindole

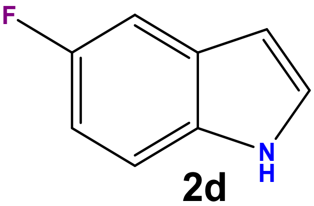

2d

^{1}H NMR (400 MHz, Acetonitrile-*d*$_3$) δ 7.38 (dd, *J* = 8.8, 4.6 Hz, 1H), 7.30 – 7.27 (m, 1H), 7.23 (dd, *J* = 10.0, 2.7 Hz, 1H), 6.92 – 6.86 (m, 1H), 6.43 (d, *J* = 2.1 Hz, 1H) ppm. **HR ESI+ MS (CH_2Cl_2):** [M+H$^+$] calcd. for $[C_8H_7FN]^+$ 136.0557, found 136.0551.

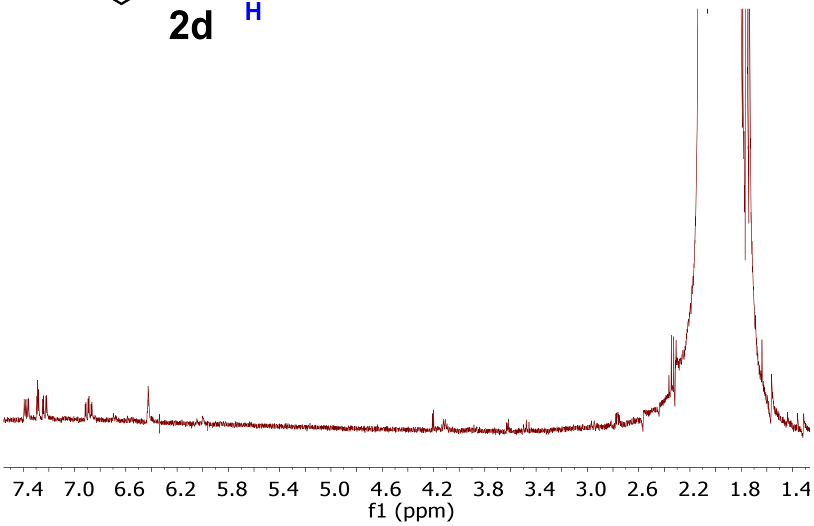

Figure S27. ^{1}H NMR spectrum in CD_3CN for the crude **2d** of the photocatalytic oxidation of indoline (**1d,** 10 mM) in the presence of the photocatalyst **[Ir3]Cl** (1 mol%) in CH_3CN under O_2 and blue LED irradiation (λ_{exc} = 460 nm) at room temperature.

2e: 2-Methylindole

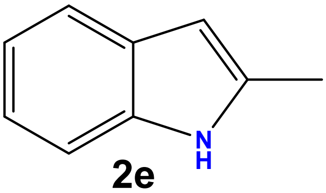

2e

^{1}H NMR (400 MHz, Acetonitrile-*d_3*) δ 7.50 (dd, *J* = 7.4, 5.9 Hz, 1H), 7.30 – 7.20 (m, 1H), 7.11 (s, 1H), 6.95 (dd, *J* = 28.3, 8.1 Hz, 1H), 6.82 (d, *J* = 19.4 Hz, 1H), 3.64 – 3.61 (m, 3H) ppm. **HR ESI+ MS (CH_2Cl_2):** [M+H$^+$] calcd. for $[C_9H_{10}N]^+$ 132.0808, found 132.0812.

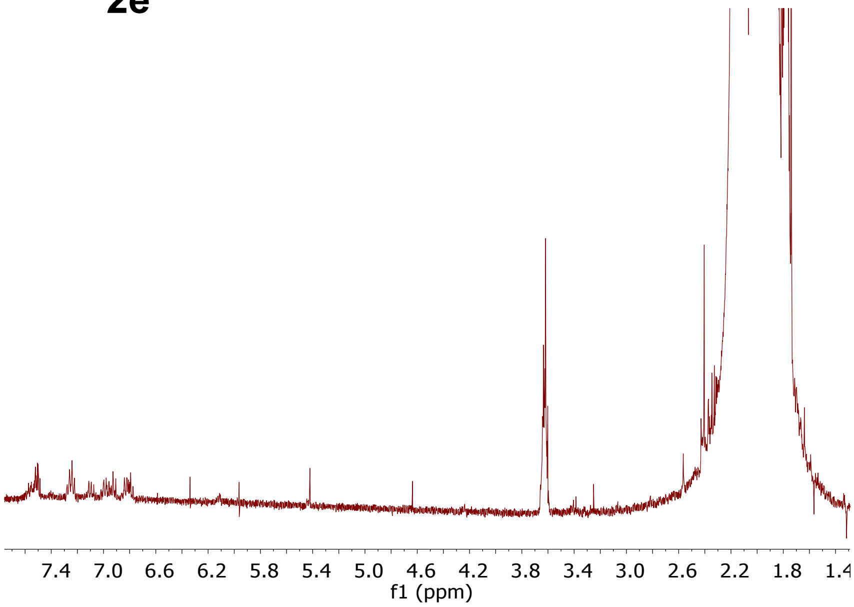

Figure S28. ^{1}H NMR spectrum in CD_3CN for the crude **2e** of the photocatalytic oxidation of indoline (**1e,** 10 mM) in the presence of the photocatalyst **[Ir3]Cl** (0.3 mol%) in CH_3CN under O_2 and blue LED irradiation (λ_{exc} = 460 nm) at room temperature.

2g: 6-Nitroindol

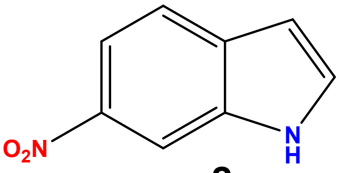

2g

^{1}H NMR (400 MHz, Acetonitrile-*d_3*) δ 8.38 (s, 1H), 7.91 (d, *J* = 8.8 Hz, 1H), 7.67 (s, 1H), 7.59 (d, *J* = 2.8 Hz, 1H), 6.63 (s, 1H) ppm.

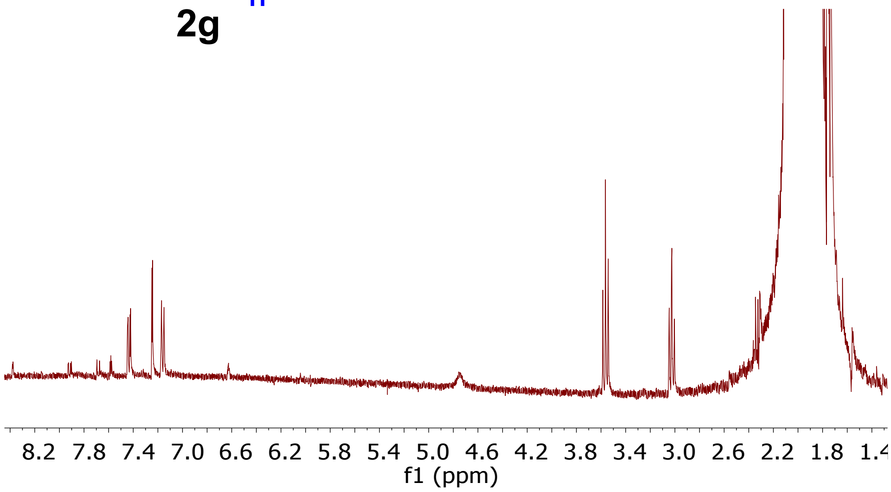

Figure S29. ^{1}H NMR spectrum in CD_3CN for the crude **2g** of the photocatalytic oxidation of indoline (**1g,** 10 mM) in the presence of the photocatalyst **[Ir3]Cl** (1.5 mol%) in CH_3CN under O_2 and blue LED irradiation (λ_{exc} = 460 nm) at room temperature.

^{1}H NMR spectra and characterization of the crude quinolines

4a: Quinoline

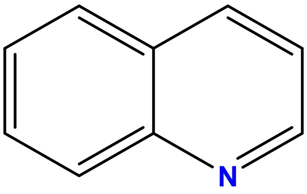

4a

^{1}H NMR (400 MHz, Acetonitrile-*d*$_3$) δ 8.89 (s, 1H), 8.30 (d, *J* = 7.4 Hz, 1H), 8.04 (d, *J* = 7.3 Hz, 1H), 7.92 (d, *J* = 6.8 Hz, 1H), 7.75 (t, *J* = 6.9 Hz, 1H), 7.63 – 7.56 (m, 1H), 7.48 (dd, *J* = 8.3, 4.2 Hz, 1H) ppm. **HR ESI+ MS (CH_2Cl_2):** [M+H$^+$] calcd. for $[C_9H_8N]^+$ 130.0652, found 130.0654.

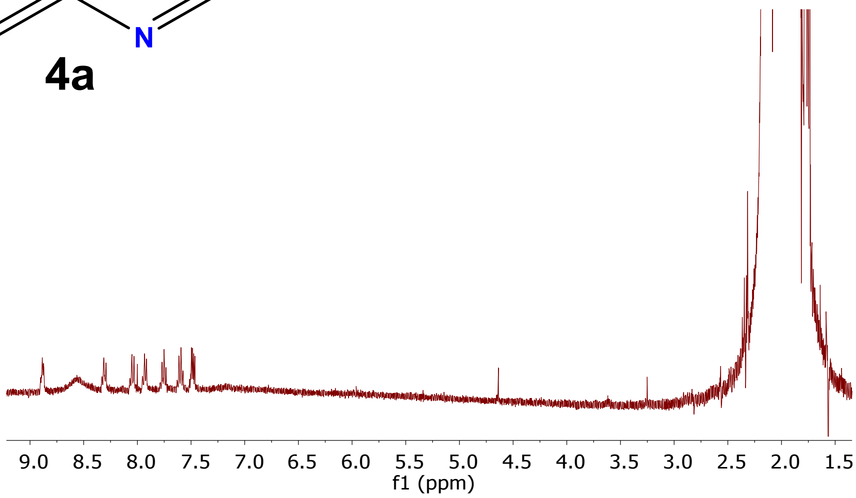

Figure S30. ^{1}H NMR spectrum in CD_3CN for the crude **4a** of the photocatalytic oxidation of tetrahydroquinoline (**3a,** 10 mM) in the presence of the photocatalyst **[Ir4]Cl** (0.7 mol%) in CH_3CN under O_2 and blue LED irradiation (λ_{exc} = 460 nm) at room temperature.

4b: 3,4-Dihydroisoquinoline

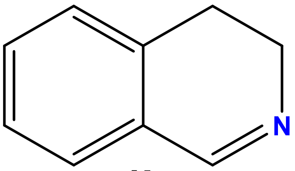

4b

^{1}H NMR (400 MHz, Acetonitrile-*d*$_3$) δ 8.27 (s, 1H), 7.39 – 7.32 (m, 1H), 7.31 – 7.27 (m, 2H), 7.18 (d, *J* = 7.2 Hz, 1H), 3.69 – 3.62 (m, 2H), 2.73 – 2.66 (m, 2H) ppm. **HR ESI+ MS (CH_2Cl_2):** [M+H$^+$] calcd. for $[C_9H_{10}N]^+$ 132.0808, found 132.0806.

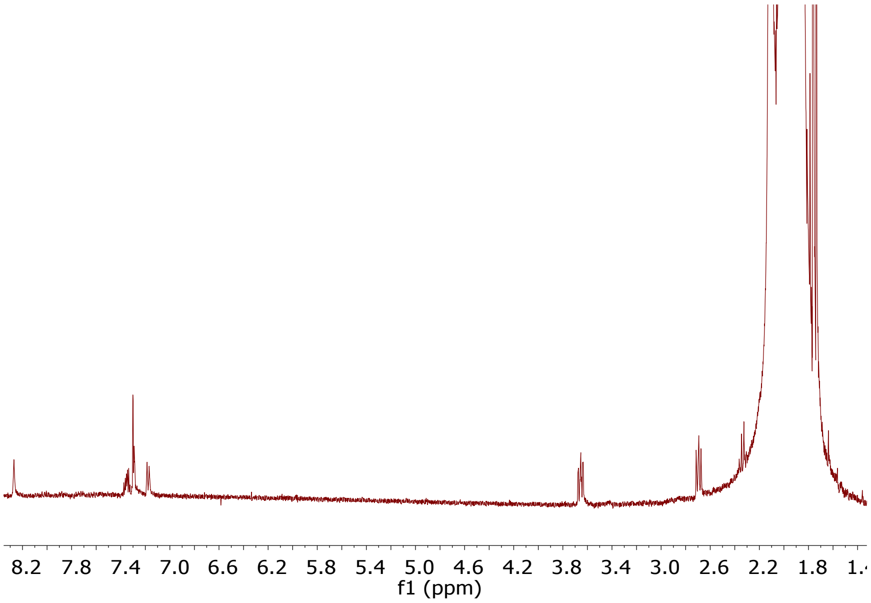

Figure S31. ^{1}H NMR spectrum in CD_3CN for the crude **4b** of the photocatalytic oxidation of tetrahydroquinoline (**3b,** 10 mM) in the presence of the photocatalyst **[Ir4]Cl** (0.7 mol%) in CH_3CN under O_2 and blue LED irradiation (λ_{exc} = 460 nm) at room temperature.

4c: 7-Nitroquinoline

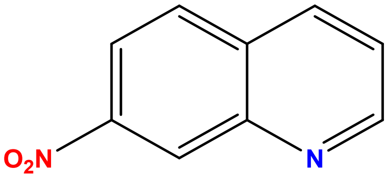

4c

^{1}H NMR (400 MHz, Acetonitrile-*d*$_3$) δ 9.06 (dd, *J* = 4.2, 1.8 Hz, 1H), 8.88 (d, *J* = 2.3 Hz, 1H), 8.41 (dd, *J* = 8.3, 2.0 Hz, 1H), 8.30 (dd, *J* = 9.0, 2.3 Hz, 1H), 8.12 (d, *J* = 9.0 Hz, 1H), 7.66 (dd, *J* = 8.4, 4.2 Hz, 1H) ppm. **HR ESI+ MS (CH_2Cl_2):** [M+H$^+$] calcd. for $[C_9H_7N_2O_2]^+$ 175.0502, found 175.0503.

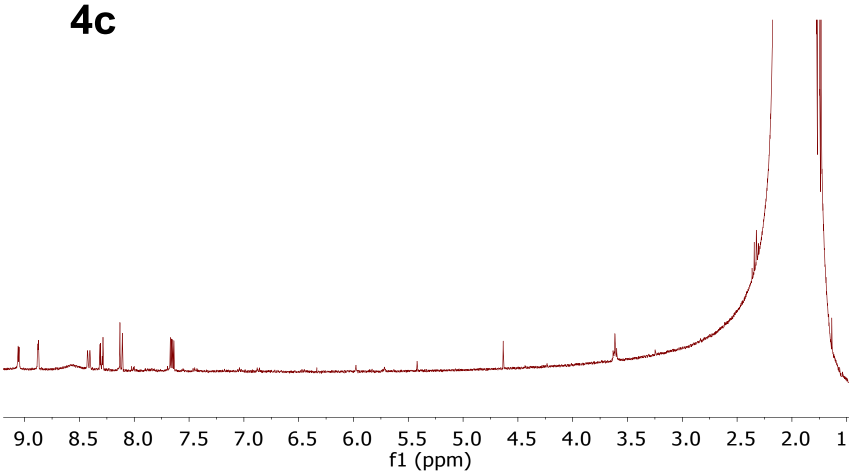

Figure S32. ^{1}H NMR spectrum in CD_3CN for the crude **4c** of the photocatalytic oxidation of tetrahydroquinoline (**3c,** 10 mM) in the presence of the photocatalyst **[Ir4]Cl** (5 mol%) in CH_3CN under O_2 and blue LED irradiation (λ_{exc} = 460 nm) at room temperature.

4e: Quinaldine

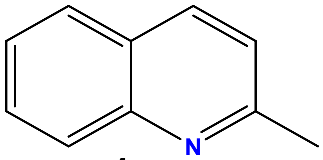

4e

^{1}H NMR (400 MHz, Acetonitrile-*d*$_3$) δ 8.12 (d, *J* = 8.8 Hz, 1H), 7.90 (d, *J* = 8.4 Hz, 1H), 7.84 (d, *J* = 7.4 Hz, 1H), 7.67 (t, *J* = 6.9 Hz, 1H), 7.50 (d, *J* = 6.9 Hz, 1H), 7.34 (d, *J* = 8.3 Hz, 1H), 2.65 (s, 3H) ppm. **HR ESI+ MS (CH_2Cl_2):** [M+H$^+$] calcd. for $[C_{10}H_{10}N]^+$ 144.0808, found 144.0810.

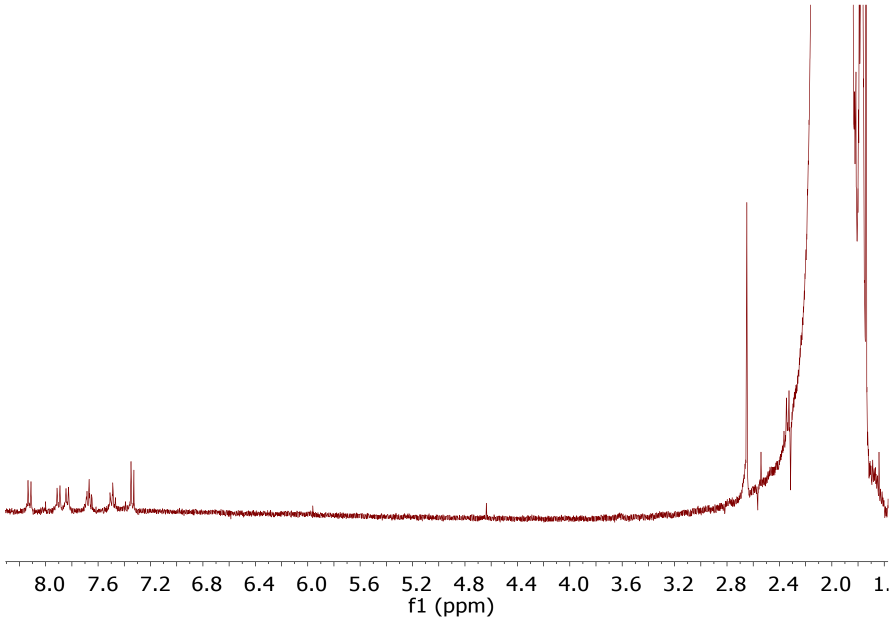

Figure S33. ^{1}H NMR spectrum in CD_3CN for the crude **4e** of the photocatalytic oxidation of tetrahydroquinoline (**3e,** 10 mM) in the presence of the photocatalyst **[Ir4]Cl** (0.7 mol%) in CH_3CN under O_2 and blue LED irradiation (λ_{exc} = 460 nm) at room temperature.

4f: 6-Methylquinoline

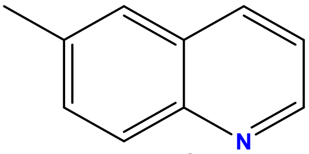

4f

^{1}H NMR (400 MHz, Acetonitrile-*d*$_3$) δ 8.79 (s, 1H), 8.15 (s, 1H), 7.91 (d, *J* = 8.6 Hz, 1H), 7.66 (s, 1H), 7.58 (s, 1H), 7.39 (dd, *J* = 8.3, 4.2 Hz, 1H), 2.51 (d, *J* = 8.2 Hz, 3H) ppm. **HR ESI+ MS (CH_2Cl_2):** [M+H$^+$] calcd. for $[C_{10}H_{10}N]^+$ 144.0808, found 144.0810.

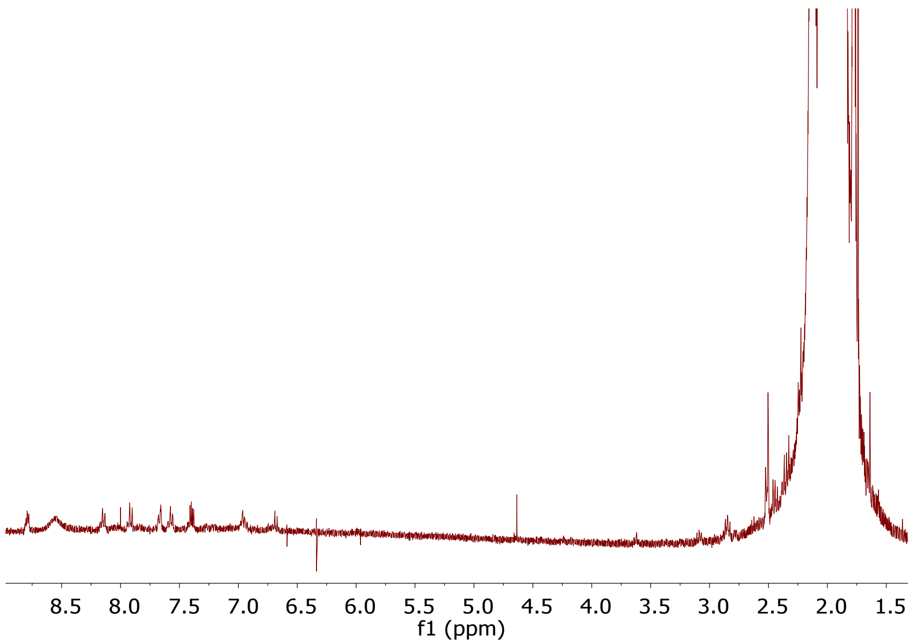

Figure S34. ^{1}H NMR spectrum in CD_3CN for the crude **4f** of the photocatalytic oxidation of tetrahydroquinoline (**3f,** 10 mM) in the presence of the photocatalyst **[Ir4]Cl** (1.5 mol%) in CH_3CN under O_2 and blue LED irradiation (λ_{exc} = 460 nm) at room temperature.

4g: 6-Methoxyquinoline

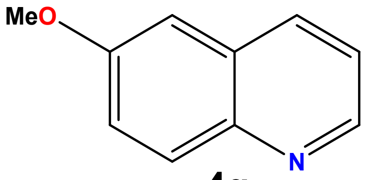

4g

^{1}H NMR (400 MHz, Acetonitrile-*d*$_3$) δ 8.71 – 8.68 (m, 1H), 8.13 (d, *J* = 8.3 Hz, 1H), 7.91 (d, *J* = 9.2 Hz, 1H), 7.41 – 7.37 (m, 1H), 7.37 – 7.32 (m, 1H), 7.24 (d, *J* = 2.8 Hz, 1H), 3.89 (s, 3H) ppm. **HR ESI+ MS (CH_2Cl_2):** [M+H$^+$] calcd. for $[C_{10}H_{10}ON]^+$ 160.0757, found 160.0757.

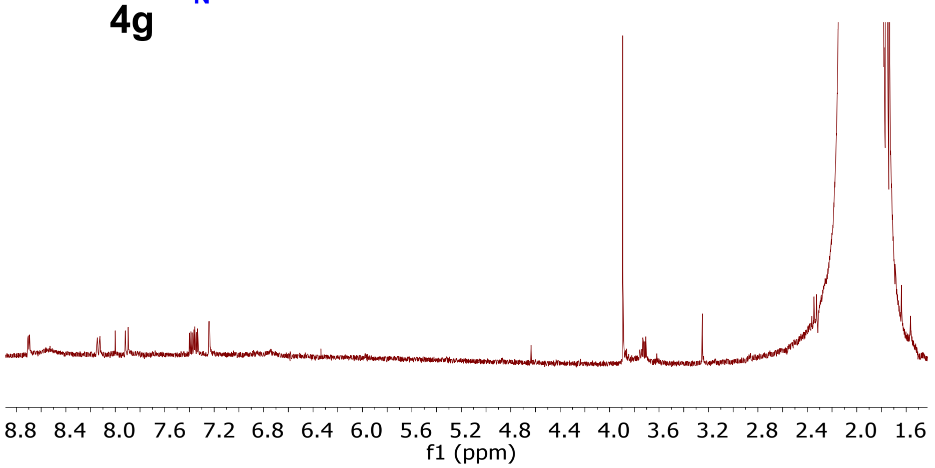

Figure S35. ^{1}H NMR spectrum in CD_3CN for the crude **4g** of the photocatalytic oxidation of tetrahydroquinoline (**3g**, 10 mM) in the presence of the photocatalyst **[Ir4]Cl** (1.5 mol%) in CH_3CN under O_2 and blue LED irradiation (λ_{exc} = 460 nm) at room temperature.

4h: Acridine

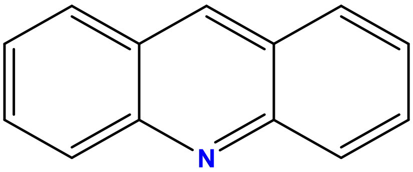

4h

^{1}H NMR (400 MHz, Acetonitrile-*d*$_3$) δ 8.94 (s, 1H), 8.25 – 8.03 (m, 4H), 7.92 – 7.74 (m, 2H), 7.70 – 7.53 (m, 2H) ppm. **HR ESI+ MS (CH_2Cl_2):** [M+H$^+$] calcd. for $[C_{13}H_{10}N]^+$ 180.0808, found 180.0809.

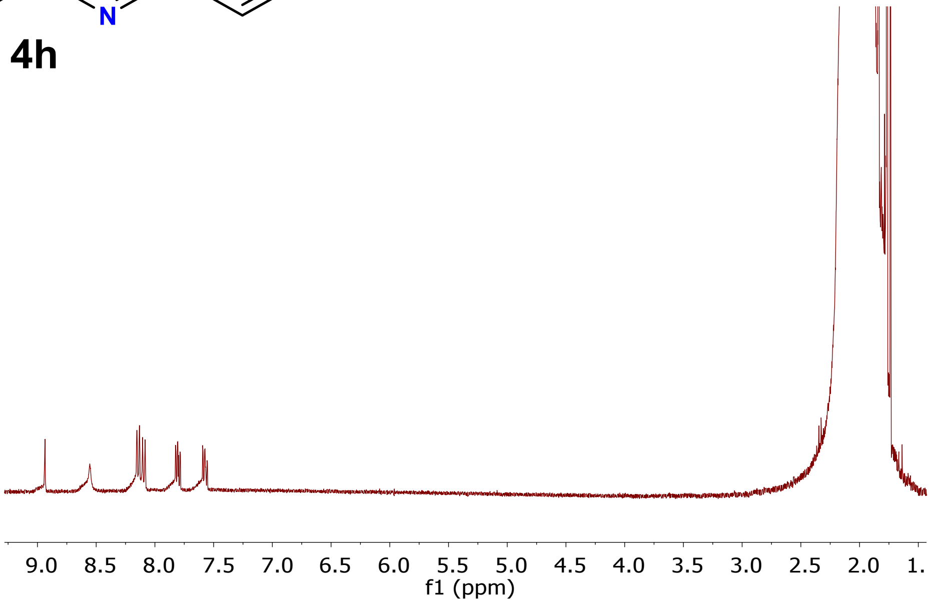

Figure S36. ^{1}H NMR spectrum in CD_3CN for the crude **4h** of the photocatalytic oxidation of tetrahydroquinoline (**3h**, 10 mM) in the presence of the photocatalyst **[Ir4]Cl** (0.7 mol%) in CH_3CN under O_2 and blue LED irradiation (λ_{exc} = 460 nm) at room temperature.

4i: Quinoxaline

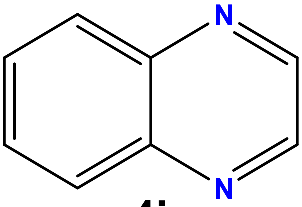

4i

^{1}H NMR (400 MHz, Acetonitrile-*d*$_3$) δ 8.83 (s, 2H), 8.06 (dd, *J* = 6.3, 3.5 Hz, 2H), 7.79 (dd, *J* = 6.4, 3.5 Hz, 2H) ppm. **HR ESI+ MS (CH_2Cl_2):** [M+H$^+$] calcd. for $[C_8H_7N_2]^+$ 131.0604, found 131.0601.

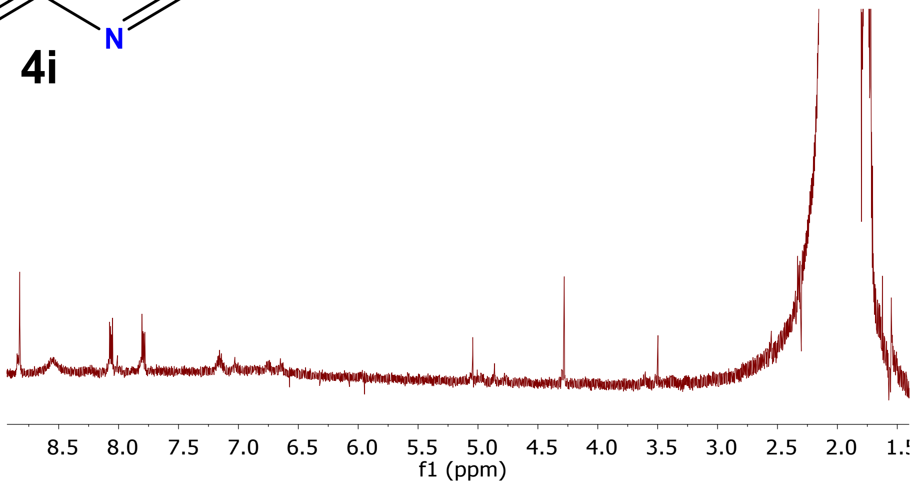

Figure S37. ^{1}H NMR spectrum in CD_3CN for the crude **4i** of the photocatalytic oxidation of tetrahydroquinoline (**3i**, 10 mM) in the presence of the photocatalyst **[Ir4]Cl** (1.5 mol%) in CH_3CN under O_2 and blue LED irradiation (λ_{exc} = 460 nm) at room temperature.

4j: 2-Methylquinoxaline

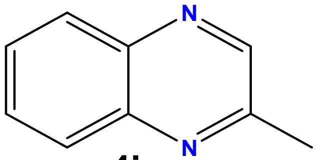

4j

^{1}H NMR (400 MHz, Acetonitrile-d_3) δ 8.75 (d, J = 6.1 Hz, 1H), 8.06 – 7.92 (m, 2H), 7.77 – 7.68 (m, 2H), 2.69 (d, J = 6.3 Hz, 3H) ppm. **HR ESI+ MS (CH_2Cl_2):** [M+H$^+$] calcd. for $[C_9H_9N_2]^+$ 145.0760, found 145.0761.

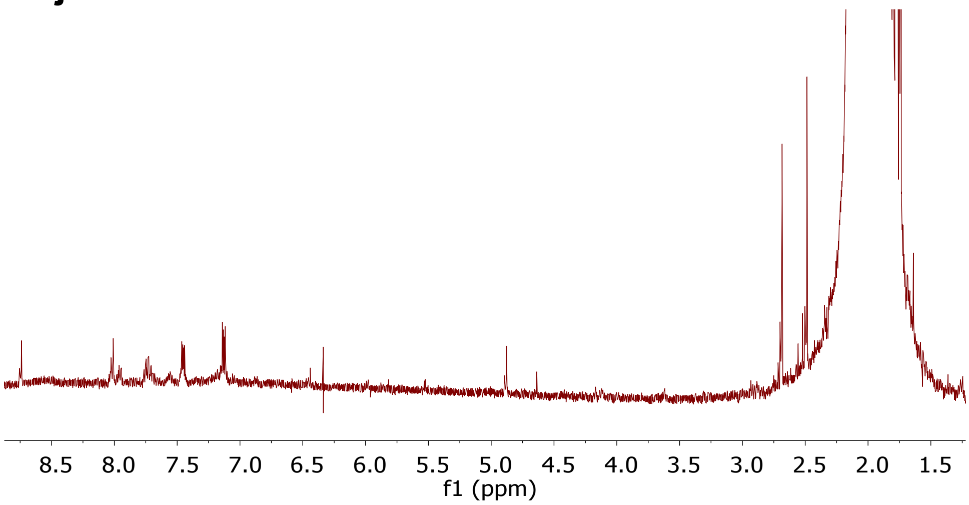

Figure S38. ^{1}H NMR spectrum in CD_3CN for the crude **4j** of the photocatalytic oxidation of tetrahydroquinoline (**3j**, 10 mM) in the presence of the photocatalyst **[Ir4]Cl** (3 mol%) in CH_3CN under O_2 and blue LED irradiation (λ_{exc} = 460 nm) at room temperature.

4k: 2,3-Dimethylquinoxaline

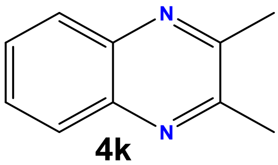

4k

^{1}H NMR (400 MHz, Acetonitrile-d_3) δ 7.91 (dd, J = 6.3, 3.5 Hz, 2H), 7.69 – 7.62 (m, 2H), 2.65 (s, 6H) ppm. **HR ESI+ MS (CH_2Cl_2):** [M+H$^+$] calcd. for $[C_{10}H_{11}N_2]^+$ 159.0917, found 159.0919.

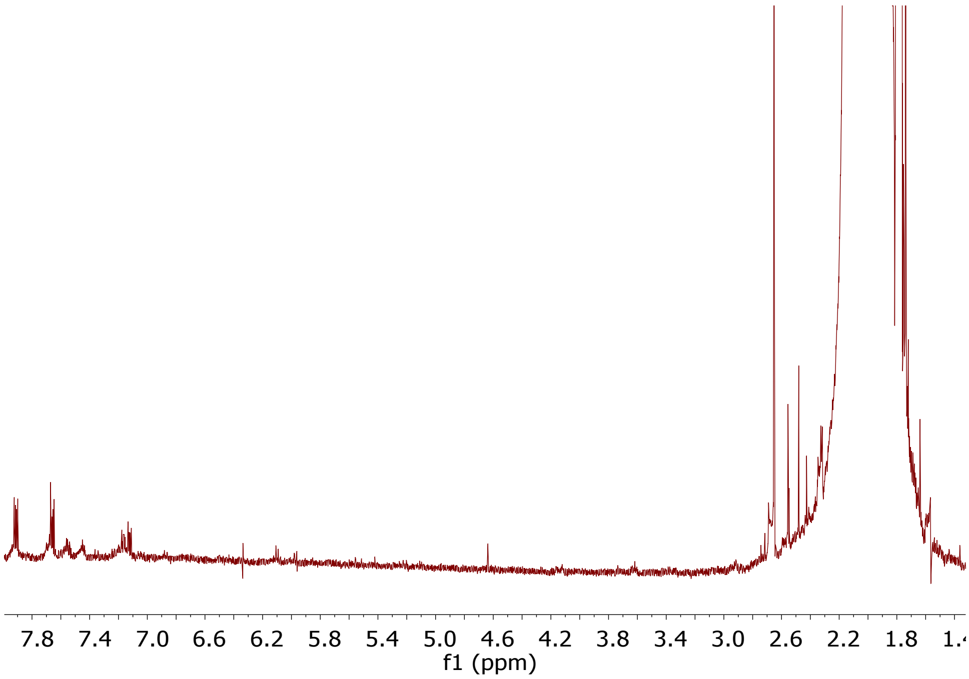

Figure S39. ^{1}H NMR spectrum in CD_3CN for the crude **4k** of the photocatalytic oxidation of tetrahydroquinoline (**3k**, 10 mM) in the presence of the photocatalyst **[Ir4]Cl** (5 mol%) in CH_3CN under O_2 and blue LED irradiation (λ_{exc} = 460 nm) at room temperature.

^{1}H and ^{13}C NMR spectra and characterization of the isolated products

2a: Indole

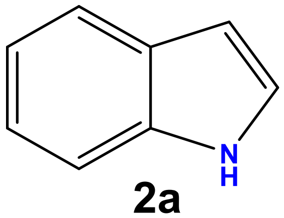

2a

^{1}H NMR (300 MHz, Chloroform-*d*) δ 7.97 (s, 1H), 7.76 (ddt, *J* = 7.6, 1.7, 0.8 Hz, 1H), 7.41 (dq, *J* = 8.1, 1.0 Hz, 1H), 7.30 (ddd, *J* = 8.1, 7.0, 1.4 Hz, 1H), 7.26 (s, 1H), 7.18 (dd, *J* = 3.2, 2.4 Hz, 1H), 6.64 (ddd, *J* = 3.1, 2.0, 1.0 Hz, 1H) ppm. **^{13}C NMR (75 MHz, $CDCl_3$)** δ 135.82, 127.89, 124.30, 122.04, 120.80, 119.89, 111.17, 102.56 ppm. **HR ESI+ MS (CH_2Cl_2):** [$M+H^+$] calcd. for $[C_8H_8N]^+$ 118.0651, found 118.0648.

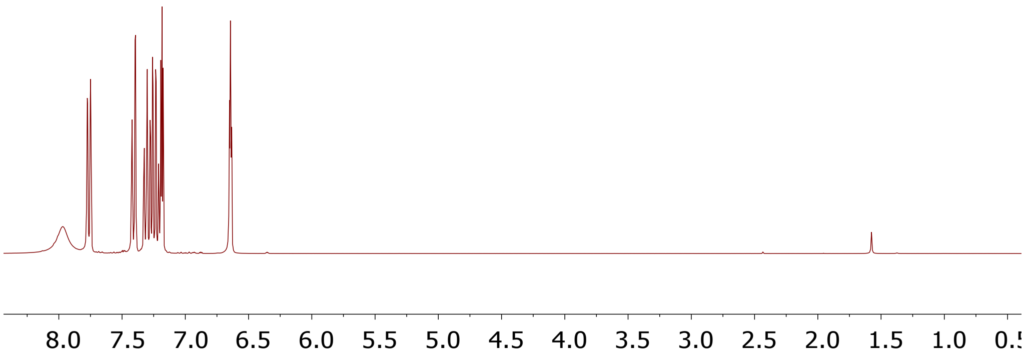

Figure S40. ^{1}H NMR spectrum in $CDCl_3$ for the isolated indole **2a** of the photocatalytic oxidation of tetrahydroquinoline (**1a,** 168 mM) in the presence of the photocatalyst **[Ir3]Cl** (0.3 mol%) in CH_3CN under O_2 and blue LED irradiation (λ_{exc} = 460 nm) at room temperature.

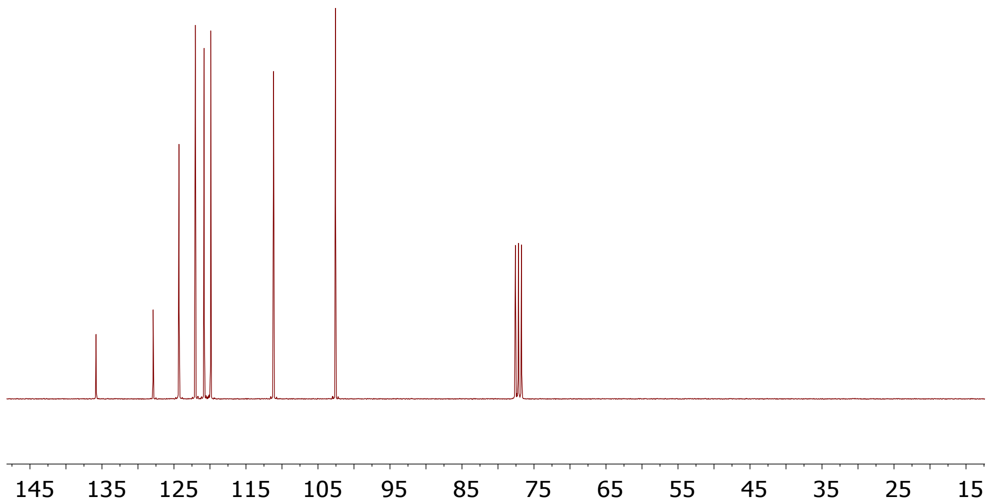

Figure S41. ^{13}C NMR spectrum in $CDCl_3$ for the isolated indole **2a** of the photocatalytic oxidation of tetrahydroquinoline (**1a,** 168 mM) in the presence of the photocatalyst **[Ir3]Cl** (0.3 mol%) in CH_3CN under O_2 and blue LED irradiation (λ_{exc} = 460 nm) at room temperature.

4a: Quinoline

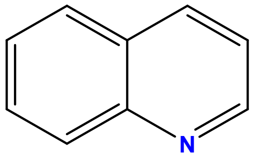

4a

^{1}H NMR (300 MHz, Chloroform-*d*) δ 8.93 (dd, *J* = 4.2, 1.7 Hz, 1H), 8.14 (ddq, *J* = 13.9, 8.4, 0.9 Hz, 2H), 7.83 (dd, *J* = 8.2, 1.4 Hz, 1H), 7.72 (ddd, *J* = 8.5, 6.9, 1.5 Hz, 1H), 7.55 (ddd, *J* = 8.2, 6.9, 1.2 Hz, 1H), 7.41 (ddd, *J* = 8.3, 4.2, 1.0 Hz, 1H) ppm. **^{13}C NMR (75 MHz, $CDCl_3$)** δ 150.55, 148.43, 136.21, 129.60, 128.44, 127.92, 126.69, 121.22 ppm. **HR ESI+ MS (CH_2Cl_2):** [M+H$^+$] calcd. for $[C_9H_8N]^+$ 130.0652, found 130.0654.

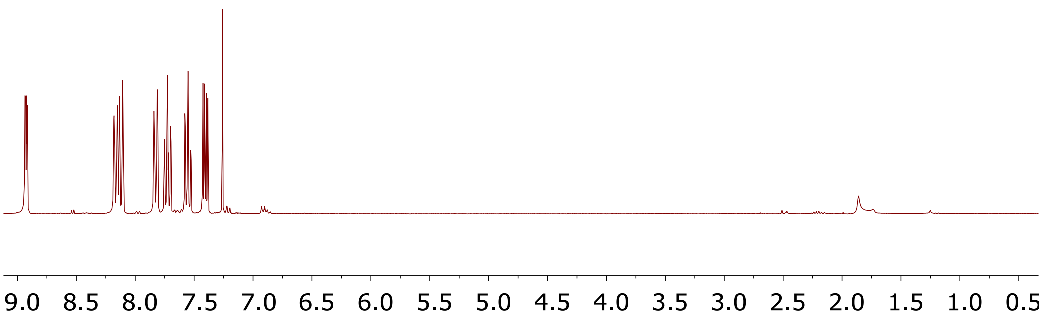

Figure S42. ^{1}H NMR spectrum in $CDCl_3$ for the isolated quinoline **4a** of the photocatalytic oxidation of tetrahydroquinoline (**3a**, 150 mM) in the presence of the photocatalyst **[Ir4]Cl** (0.7 mol%) in CH_3CN under O_2 and blue LED irradiation (λ_{exc} = 460 nm) at room temperature.

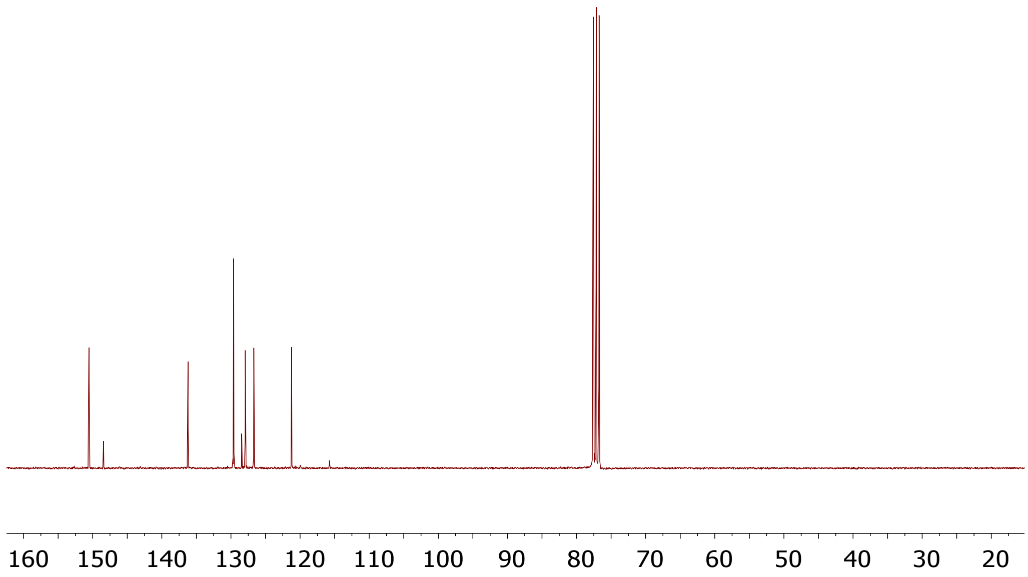

Figure S43. ^{13}C NMR spectrum in $CDCl_3$ for the isolated quinoline **4a** of the photocatalytic oxidation of tetrahydroquinoline (**3a**, 150 mM) in the presence of the photocatalyst **[Ir4]Cl** (0.7 mol%) in CH_3CN under O_2 and blue LED irradiation (λ_{exc} = 460 nm) at room temperature.

9.- Detection of H_2O_2 in photocatalytic experiments

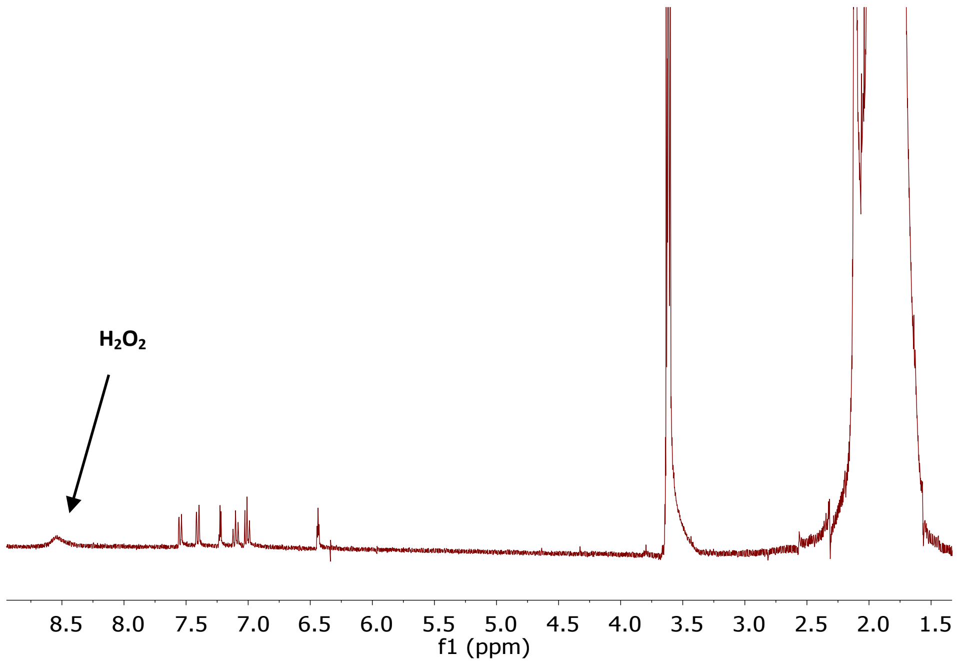

Figure S44. ^{1}H NMR spectrum in CD_3CN for the crude **2a** of the photocatalytic oxidation of indoline (**1a,** 10 mM) in the presence of the photocatalyst **[Ir3]Cl** (0.3 mol%) in CH_3CN under O_2 and blue LED irradiation (λ_{exc} = 460 nm) at room temperature, showing signal attributed to $\mathbf{H_2O_2}$.

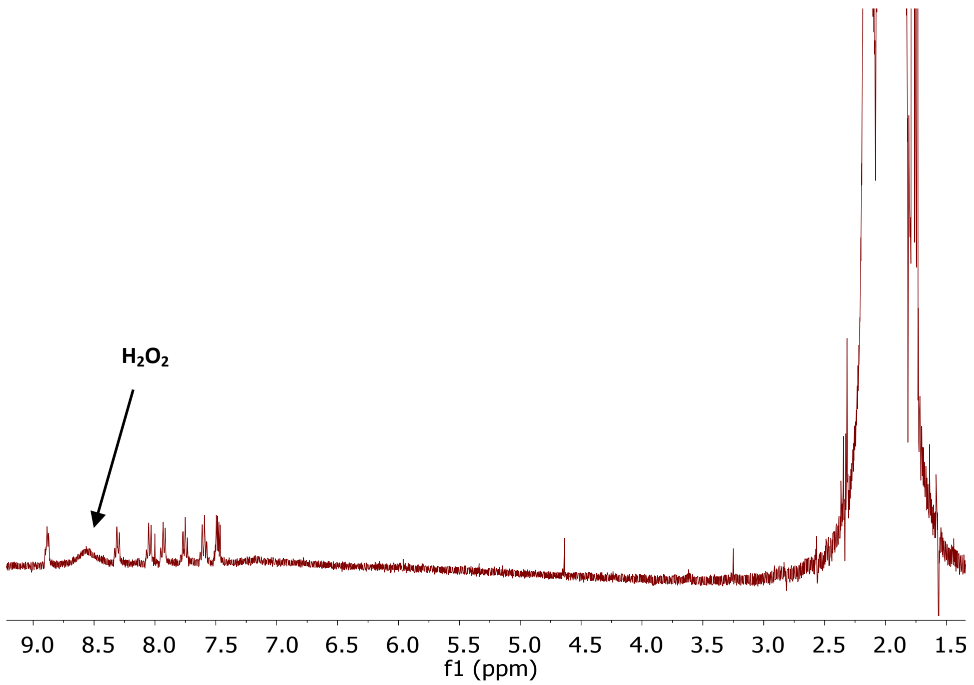

Figure S45. ^{1}H NMR spectrum in CD_3CN for the crude **4a**of the photocatalytic oxidation of tetrahydroquinoline (**3a,** 10 mM) in the presence of the photocatalyst **[Ir4]Cl** (0.7 mol%) in CH_3CN under O_2 and blue LED irradiation (λ_{exc} = 460 nm) at room temperature, showing signal attributed to **H_2O_2**.

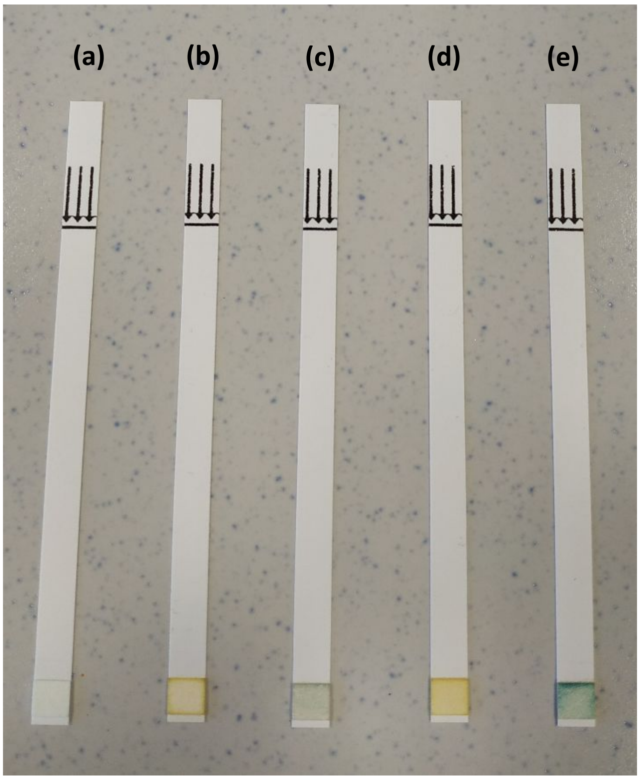

Figure S46. Detection of H_2O_2 using Quantofix® peroxide sticks (range 0-25 mg/L H_2O_2, Sigma-Aldrich) for semiquantitative determination of peroxide. **(a)** Non used stick **(b)** Control experiment **(c)** The stick was introduced in the crude mixture of a photocatalytic experiment (photooxidation of indoline, **1a**, in the presence of **[Ir3]Cl** (0.3 mol %)) after 24 hours of reaction and turned green/blue as a symptom of H_2O_2 presence **(d)** Control experiment **(e)** The stick was introduced in the crude mixture of a photocatalytic experiment (photooxidation of tetrahydroquinoline, **3a**, in the presence of **[Ir4]Cl** (0.7 mol %)) after 24 hours of reaction and turned green/blue as a symptom of H_2O_2 presence.

10. Stern-Volmer Experiments

Emission spectra were recorded using a Fluorescence Spectrometer Edinburgh Instruments FLS980. A solution of **[Ir4]Cl** (0.07 mM in CH_3CN) was deoxygenated through three Freeze-Pump-Thaw Cycles. Then, the photoluminescence spectrum was recorded upon irradiation at λ_{ex} = 405 nm and the emission intensity at 544 nm was collected. Increasing amounts of THQ (**3a**) were added and the solution was deoxygenated (bubbling nitrogen 2-3 minutes) every time. Thus, a decrease of **[Ir4]Cl** photoluminescence was observed upon addition of **3a** (Figure 8) and the ratio I_0/I (λ_{max} = 544 nm) was represented versus the concentration of **3a** and the experimental values were fitted to the Stern-Volmer equation (**$I_0/I = K_{sv} \times [3a] + 1$;** K_{sv} = Stern-Volmer quenching rate constant). The resulting plot proved that **3a** quenches the excited state of photocatalyst **[Ir4]Cl**.

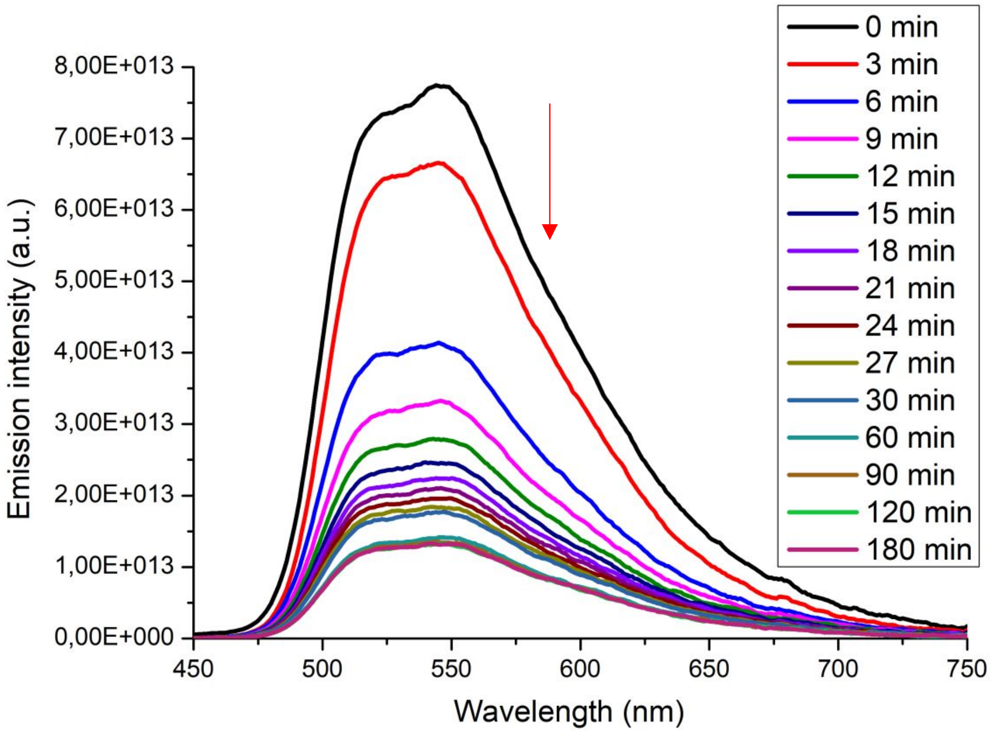

Fig. S47. Quenching Experiments in the presence of O_2. Evolution with time of the emission spectra of complex **[Ir4]Cl** (0.07 mM in CH_3CN at 25 °C) after exposure to open air.

11. Pathways A and B for the reaction mechanism

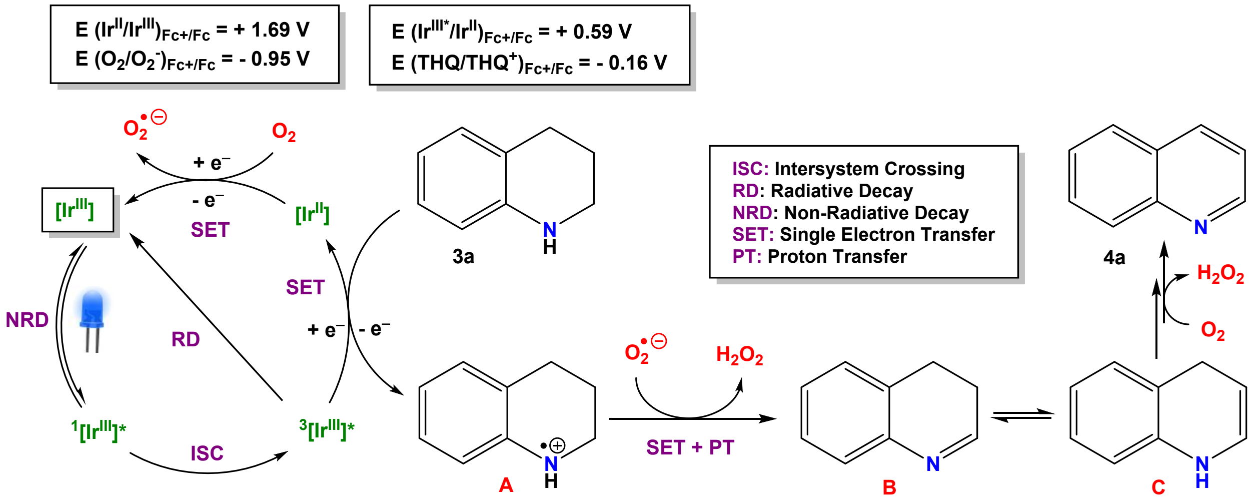

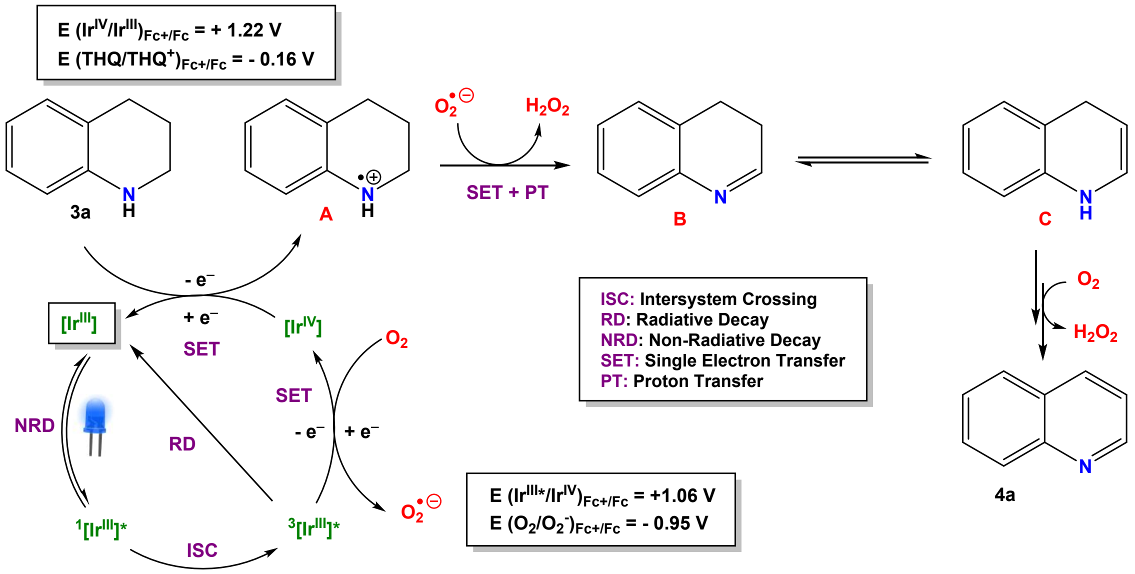

Fig. S48. Pathways A and B for the oxidative dehydrogenation of **3a** in the presence of the new Ir(III) PCs, based on the oxidative quenching of the PC.

12. Photophysical properties.

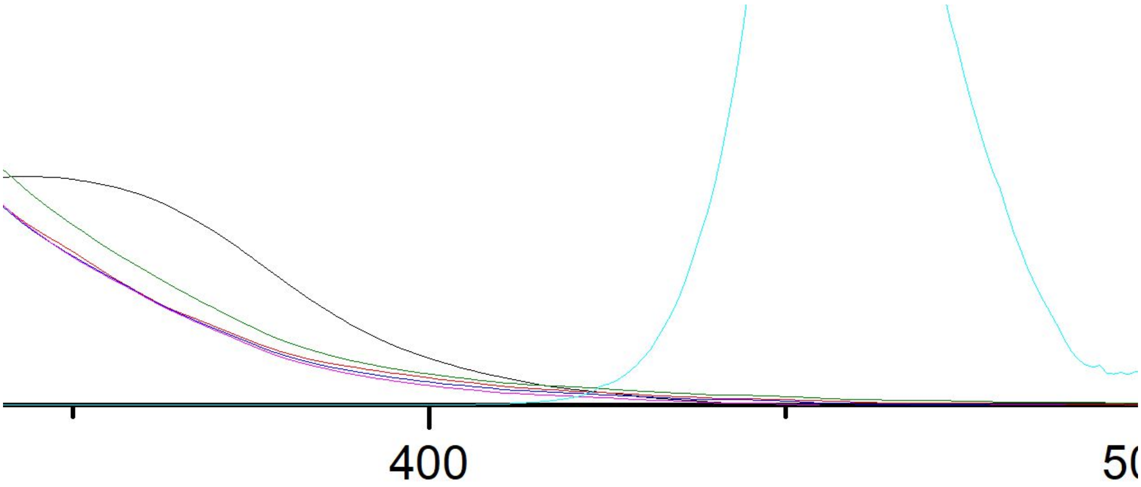

Fig. S49. Zoom of the overlaid UV-Vis absorption spectra of complexes **[Ir1]Cl**–**[Ir5]Cl** (10^{-5} M) in CH_3CN at 25 °C along with the emission spectrum of the blue light used in the photocatalytic assays.

13. References.

(1) Fulmer, G. R.; Miller, A. J. M.; Sherden, N. H.; Gottlieb, H. E.; Nudelman, A.; Stoltz, B. M.; Bercaw, J. E.; Goldberg, K. I. NMR chemical shifts of trace impurities: common laboratory solvents, organics, and gases in deuterated solvents relevant to the organometallic chemist. *Organometallics* **2010**, 29, 2176–2179.

(2) Sprintschnik, G.; Sprintschnik, H. W.; Kirsch, P. P.; Whitten, D. G. Preparation and photochemical reactivity of surfactant ruthenium (II) complexes in monolayer assemblies and at water-solid interfaces. *J. Am. Chem. Soc.* **1977**, 99, 4947–4954.

(3) Maynard, J.A.; Rae, I.D.; Rash, D.; Swan, J.M. Reaction of 2-(4'-Thiazolyl) benzimidazole (thiabendazole) with alkyl halides. *Aust. J. Chem*., **1971***,* 24, 1873-1881.

(4) Huang, W.-K.; Cheng, C.-W.; Chang, S.-M.; Lee, Y.-P.; Diau, E. W.-G. Synthesis and electron-transfer properties of benzimidazole-functionalized ruthenium complexes for highly efficient dye-sensitized solar cells. *Chem. Commun.* **2010**, 46, 8992–8994.

(5) Shavaleev, N. M.; Bell, Z. R.; Easun, T. L.; Rutkaite, R.; Ward, M. D. Complexes of substituted derivatives of 2-(2-pyridyl) benzimidazole with Re (I), Ru (II) and Pt (II): structures, redox and luminescence properties. *Dalton Trans.* **2004**, 21, 3678–3688.

(6) SAINT v8.37. Bruker-AXS, APEX3 v2016.1.0.: Madison, Wisconsin, USA 2016.

(7) Krause, L.; Herbst-Irmer, R.; Sheldrick, G. M.; Stalke, D. Comparison of Silver and Molybdenum Microfocus X-Ray Sources for Single-Crystal Structure Determination. *J. Appl. Crystallogr.* **2015**, 48, 3–10.

(8) Farrugia, L. J. WinGX and ORTEP for Windows : An Update. *J. Appl. Crystallogr.* **2012**, 45, 849–854.

(9) Sheldrick, G. M. SHELX-2014, Program for Crystal Structure Refinement. University of Göttingen: Göttingen, Germany 2014.

(10) Frisch, M. J.; Trucks, G. W.; Schlegel, H. B.; Scuseria, G. E.; Robb, M. A.; Cheeseman, J. R.; Scalmani, G.; Barone, V.; Mennucci, B.; Petersson, G. A.; Nakatsuji, H.; Caricato, M.; Li, X.; Hratchian, H. P.; Izmaylov, A. F.; Bloino, J.; Zheng, G.; Sonnenberg, J. L.; Hada, M.; Ehara, M.; Toyota, K.; Fukuda, R.; Hasegawa, J.; Ishida, M.; Nakajima, T.; Honda, Y.; Kitao, O.; Nakai, H.; Vreven, T.; Montgomery Jr., J. A.; Peralta, J. E.; Ogliaro, F.; Bearpark, M.; Heyd, J. J.; Brothers, E.; Kudin, K. N.; Staroverov, V. N.; Kobayashi, R.; Normand, J.; Raghavachari, K.; Rendell, A.; Burant, J. C.; Iyengar, S. S.; Tomasi, J.; Cossi, M.; Rega, N.; Millam, J. M.; Klene, M.; Knox, J. E.; Cross, J. B.; Bakken, V.; Adamo, C.; Jaramillo, J.; Gomperts, R.; Stratmann, R. E.; Yazyev, O.; Austin, A. J.; Cammi, R.; Pomelli, C.; Ochterski, J. W.; Martin, R. L.; Morokuma, K.; Zakrzewski, V. G.; Voth, G. A.; Salvador, P.; Dannenberg, J. J.; Dapprich, S.; Daniels, A. D.; Farkas, Ö.; Foresman, J. B.; Ortiz, J. V.; Cioslowski, J.; Fox, D. J. *Gaussian 09* (Gaussian, Inc., Wallingford CT, 2009).

(11) Becke, A. D. Density-functional thermochemistry. III. The role of exact exchange. *J. Chem. Phys.* **1993**, 98, 5648–5652.

(12) Lee, C.; Yang, W.; Parr, R. G. Development of the Colle-Salvetti correlation-energy formula into a functional of the electron density. *Phys. Rev. B* **1988**, 37, 785–789.

(13) Francl, M. M.; Pietro, W. J.; Hehre, W. J.; Binkley, J. S.; Gordon, M. S.; DeFrees, D. J.; Pople, J. A. Self-consistent molecular orbital methods. XXIII. A polarization-type basis set for second-row elements. *J. Chem. Phys.* **1982**, 77, 3654–3665.

(14) Hariharan, P. C.; Pople, J. A. The influence of polarization functions on molecular orbital hydrogenation energies. *Theor. Chim. Acta* **1973**, 28, 213–222.

(15) Scalmani, G.; Frisch, M. J. Continuous surface charge polarizable continuum models of solvation. I. General formalism. *J. Chem. Phys.* **2010**, 132, 114110.

(16) Marenich, A. V; Cramer, C. J.; Truhlar, D. G. Generalized born solvation model SM12. *J. Phys. Chem. B* **2009**, 113, 6378–6396.

(17) Connelly, N. G.; Geiger, W. E. Chemical redox agents for organometallic chemistry. *Chem. Rev.* **1996**, 96, 877−910.
